# Petasin potently inhibits mitochondrial complex I–based metabolism that supports tumor growth and metastasis

**DOI:** 10.1172/JCI139933

**Published:** 2021-09-01

**Authors:** Kazuki Heishima, Nobuhiko Sugito, Tomoyoshi Soga, Masashi Nishikawa, Yuko Ito, Ryo Honda, Yuki Kuranaga, Hiroki Sakai, Ryo Ito, Takayuki Nakagawa, Hiroshi Ueda, Yukihiro Akao

**Affiliations:** 1 The United Graduate School of Drug Discovery and Medical Information Sciences, Gifu University, Gifu, Gifu, Japan.; 2 Institute for Advanced Biosciences, Keio University, Tsuruoka, Yamagata, Japan.; 3 Department of Anatomy and Cell Biology, Division of Life Sciences, Osaka Medical College, Takatsuki, Osaka, Japan.; 4 Laboratory of Veterinary Pathology, Joint Department of Veterinary Medicine, Faculty of Applied Biological Sciences, Gifu University, Gifu, Gifu, Japan.; 5 CCI Holdings Inc., Seki, Gifu, Japan.; 6 Laboratory of Veterinary Surgery, Graduate School of Agricultural and Life Sciences, The University of Tokyo, Bunkyo, Tokyo, Japan.

**Keywords:** Metabolism, Oncology, Amino acid metabolism, Cancer, Mitochondria

## Abstract

Mitochondrial electron transport chain complex I (ETCC1) is the essential core of cancer metabolism, yet potent ETCC1 inhibitors capable of safely suppressing tumor growth and metastasis in vivo are limited. From a plant extract screening, we identified petasin (PT) as a highly potent ETCC1 inhibitor with a chemical structure distinct from conventional inhibitors. PT had at least 1700 times higher activity than that of metformin or phenformin and induced cytotoxicity against a broad spectrum of tumor types. PT administration also induced prominent growth inhibition in multiple syngeneic and xenograft mouse models in vivo. Despite its higher potency, it showed no apparent toxicity toward nontumor cells and normal organs. Also, treatment with PT attenuated cellular motility and focal adhesion in vitro as well as lung metastasis in vivo. Metabolome and proteome analyses revealed that PT severely depleted the level of aspartate, disrupted tumor-associated metabolism of nucleotide synthesis and glycosylation, and downregulated major oncoproteins associated with proliferation and metastasis. These findings indicate the promising potential of PT as a potent ETCC1 inhibitor to target the metabolic vulnerability of tumor cells.

## Graphical abstract

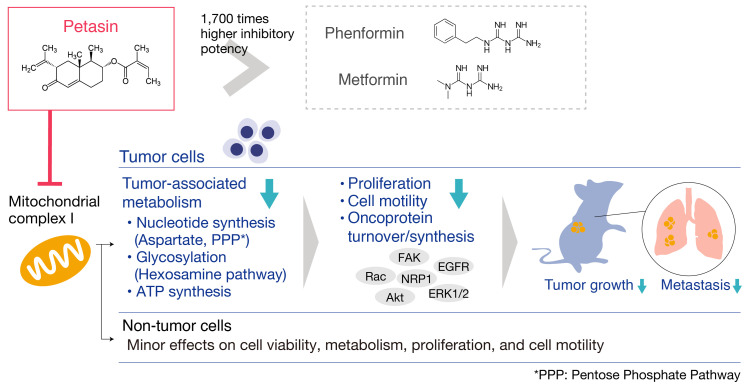

## Introduction

Cancer cells exhibit addiction to a specific metabolism (1, 2). Well-recognized examples are upregulated incorporation of glucose as in aerobic glycolysis or the Warburg effect (3) and that of glutamine (2), as seen in the phenomenon of the accumulation of radioactive fluorine–labeled glucose or glutamine in tumor tissue in a wide variety of cancers (4, 5). The metabolism of these 2 principal nutrients contributes to rapid tumor growth and metastasis by producing an array of metabolic intermediates used for the synthesis of cellular building blocks and numerous oncogenes after entering the glycolytic pathway or TCA cycle (6).

The essential core of these 2 metabolisms resides in mitochondrial electron transport chain complex I (ETCC1), an NADH ubiquinone oxidoreductase. ETCC1 provides the NAD that allows cancer cells to drive the action of NAD-dependent enzymes necessary for the rapid synthesis of various glucose- or glutamine-derived intermediates in the glycolytic pathway and TCA cycle (1, 6, 7). Indeed, pharmacological or genetic impairment of ETCC1 induces a severe metabolic disruption of glycolysis and glutaminolysis and diminishes the tumorigenic and metastatic activities of cancer cells (8–10).

Although ETCC1 is a crucial target to annihilate cancer-specific metabolism, currently available inhibitors of ETCC1 have major limitations for use in cancer treatment because of their lack of adequate potency, e.g., biguanides, such as metformin and phenformin (11, 12), or high toxicity, e.g., rotenone (13, 14) and BAY87-2243, terminated at phase I of a clinical trial, ClinicalTrials.gov NCT01297530 (15). In this context, the strategy of targeting ETCC1 for cancer treatment has lacked suitable modalities to accomplish its objectives, and so there has been considerable interest in the development of ETCC1 inhibitors with high potency and safety.

Here, we report the identification of petasin (PT) from *Petasites japonicus* as a highly potent and specific inhibitor of ETCC1 with 1700 times higher activity than that of metformin or phenformin. We also uncovered its potential mechanism underlying inhibition of tumor cell growth and metastasis, one involving cancer-specific metabolic disruption and subsequent inhibition of oncoprotein expression.

## Results

### Identification of PT.

**Figure 1:**
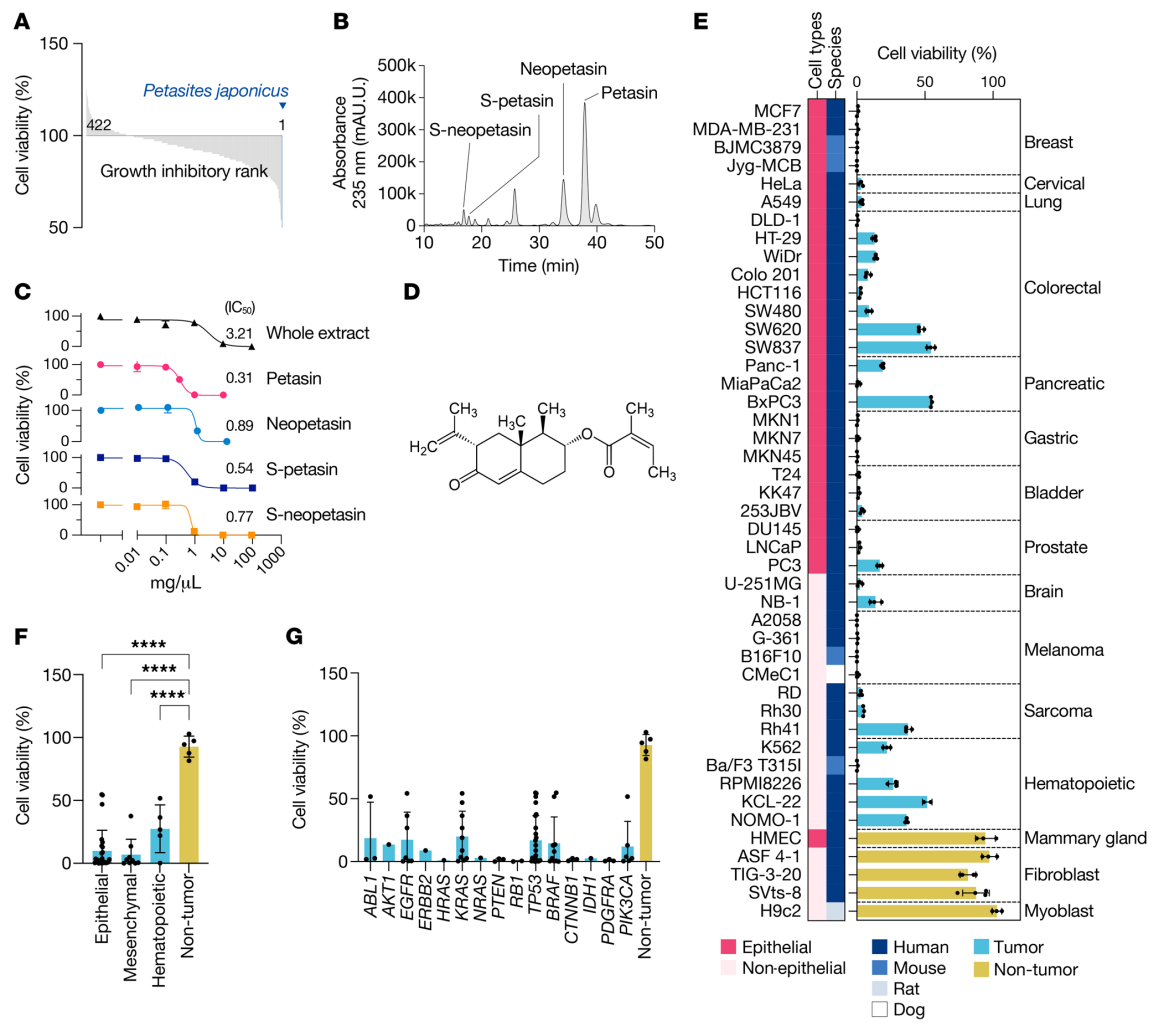
Identification of petasin and its cytotoxicity against tumor and nontumor cell lines. (**A**) Screening results from 422 kinds of plant extracts incubated with K562 cells. (**B**) HPLC analysis for bulk extract from *Petasites japonicus*. Petasin (PT) was the most abundant ingredient of the extract. (**C**) Dose-response curves for viable cell counts of CMeC1 cells treated for 72 hours with 0.01–100 μg/mL *Petasites japonicus* extract (whole extract) or its active ingredients (PT, neopetasin, S-petasin, or S-neopetasin) in DMEM supplemented with 10% FBS (*n =* 3). (**D**) Chemical structure of PT. (**E**) Viable cell percentage for tumor and nontumor cell lines treated with 3 μM PT or DMSO in DMEM supplemented with 10% FBS (*n =* 3). (**F** and **G**) Different sensitivity to PT treatment of tumor cell lines of different cell origin (**F**) or mutation status (**G**). Data are presented as the mean ± SD. ****P < 0.0001, 1-way ANOVA with Dunnett’s post hoc test.

To identify natural compounds having an antiproliferative effect on tumor cells, we designed a unique library that contained 422 kinds of extracts from herbal or edible plants mainly originating from Asia. Through screening for the cytotoxicity of these plant extracts, we found that an ethanol extract from *Petasites japonicus*, a plant native to Japan, showed the most potent cytotoxicity, having an IC_50_ of 3.21 μg/mL (Figure 1A). Fractionation of the extract by HPLC and subsequent cytotoxic screening revealed that the active ingredients of the extract were PT derivatives (Figure 1, B and C), including PT (6.39% w/w of the whole extract), neopetasin (2.07%), S-neopetasin (0.56%), and S-petasin (0.27%). These compounds separated from the extract had similar cytotoxic activity but higher potency than the bulk extract (Figure 1C). Among the PT derivatives, we focused on PT, the most abundant and potent active ingredient in the extract (Figure 1, B–D), for further analysis.

### Growth inhibitory effects of PT on tumor cell lines.

**Figure 2:**
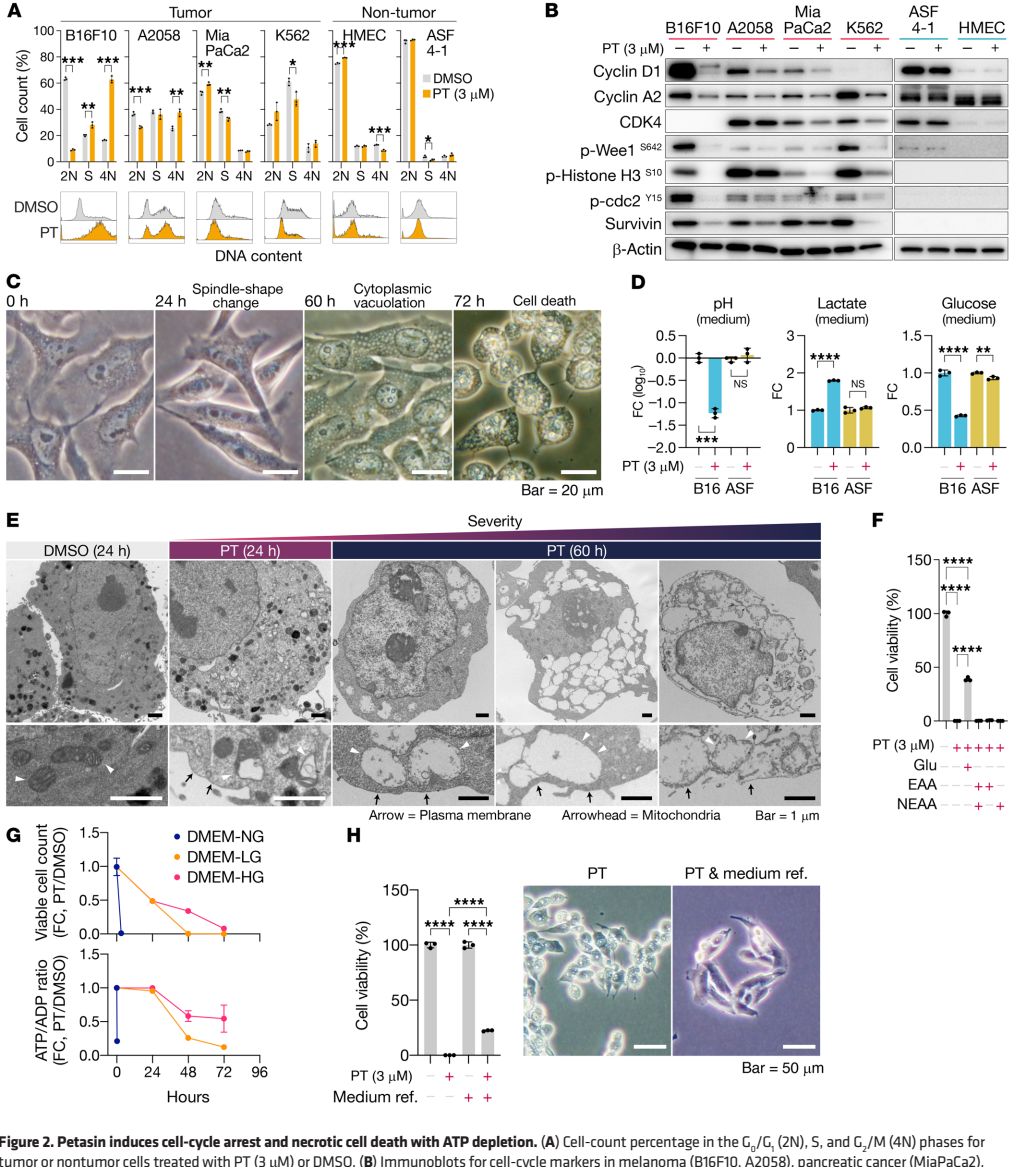
Petasin induces cell-cycle arrest and necrotic cell death with ATP depletion. (**A**) Cell-count percentage in the G_0_/G_1_ (2N), S, and G_2_/M (4N) phases for tumor or nontumor cells treated with PT (3 μM) or DMSO. (**B**) Immunoblots for cell-cycle markers in melanoma (B16F10, A2058), pancreatic cancer (MiaPaCa2), chronic myeloid leukemia (K562), and nontumor (ASF 4-1 and HMEC) cell lines. (**C**) Phase-contrast microscopic images of B16F10 cells treated for 0, 24, 60, or 72 hours with PT (3 μM) or DMSO in high-glucose DMEM. Scale bars: 20 μm. (**D**) Fold-change (FC) in pH and in glucose and lactate concentrations in medium of B16F10 or ASF 4-1 cell cultures treated for 72 hours with PT (3 μM) or DMSO in high-glucose DMEM. (**E**) Transmission electron microscopic images of B16F10 cells treated for 24 or 60 hours with PT (3 μM) or DMSO. Arrow and arrowheads indicate plasma membrane and mitochondria, respectively. Scale bars: 1 μm. (**F**) Viable cell percentage of B16F10 cells treated for 48 hours with PT (3 μM) in EMEM supplemented with or without glucose (Glu, final concentration of 4.5 g/L), essential amino acids (EAA), and nonessential amino acids (NEAA). ****P < 0.0001, 1-way ANOVA with Tukey’s post hoc test. (**G**) Time course of FC in viable cell count and ATP/ADP ratio. B16F10 cells were treated with PT (3 μM) in DMEM containing different concentrations of glucose (DMEM-NG, no glucose, 0 g/L; DMEM-LG, low glucose, 1 g/L; DMEM-HG, high glucose, 4.5 g/L). (**H**) Viable cell percentage and representative images for B16F10 cells treated with PT (3 μM) or DMSO in DMEM-HG with or without medium refreshment every 24 hours. ****P < 0.0001, 1-way ANOVA with Tukey’s post hoc test. Scale bars: 50 μm. *P < 0.05; **P < 0.01; ***P < 0.001; ****P < 0.0001; 2-tailed, unpaired Student’s *t* test unless otherwise indicated. Data are presented as the mean ± SD (*n =* 3). NS, not significant.

Although PT derivatives have been investigated in the past as agents for treating allergic diseases (16), their antitumor properties remained largely unknown. In this regard, we performed a series of experiments to reveal the potency, spectrum, and inhibitory mechanism of PT in tumor cells. As a result, we found that PT induced marked cytotoxicity toward a broad spectrum of tumor cell lines (Figure 1E). The cytotoxicity was independent of the mutated gene(s), cell origin, or animal species and was still evident in three-dimensionally cultured cells (Figure 1, E–G, and Supplemental Figure 1; supplemental material available online with this article; https://doi.org/10.1172/JCI139933DS1). We then proceeded to undertake a more detailed investigation to clarify the characteristics of PT by mainly using B16F10 cells, a well-established model for assessing both tumor growth and metastasis (17). Treatment of these cells with PT primarily induced growth inhibition with morphological changes and a decrease in the pH of the medium, resulting in cell death (Figure 2, A–E). The growth inhibition was due to severe cell-cycle arrest with downregulation of cell cycle–associated proteins, although the degree and detailed profiles were different among the cell lines tested (Figure 2, A and B). During the growth inhibition, PT-treated B16F10 cells showed a morphological change to a spindle or stellate shape (Figure 2C). Also, the medium of these cells was yellow in color, indicating low pH, and contained high lactate and low glucose levels (Figure 2D), suggesting that PT upregulated glucose uptake and lactate production in the cells.

PT treatment of the cells resulted in cell death accompanied by severe cytoplasmic vacuolations (Figure 2C). The treated cells started detaching from the culture dish after 36 to 48 hours (in Eagle’s minimum essential medium [EMEM] or low-glucose DMEM) or 60 to 72 hours had elapsed (in high-glucose DMEM), and nearly all of the cells subsequently resulted in cell death at once within a few hours (Figure 2C). Transmission electron microscopy analysis revealed that the cytoplasmic vacuoles were composed of severely damaged mitochondria (Figure 2, C and E). Also, the dying cells showed loss of plasma membrane integrity (Figure 2E), suggesting that the type of cell death was necrotic in nature. Since PT treatment likely affected glucose metabolism of tumor cells, we next assessed the association between glucose metabolism and necrotic cell death. As a result, we found that supplementation with glucose, but not essential or nonessential amino acids, canceled PT-induced necrotic cell death (Figure 2F) and that the timing was delayed in a glucose-dependent manner (Figure 2G). PT treatment under a glucose-free medium immediately induced necrotic cell death in the B16F10 cells (Figure 2G), whereas sufficient glucose supply by frequent medium refreshment completely prevented it (Figure 2H). Of note, the decrease in the viable cell count was not rescued by the frequent medium refreshment (Figure 2H), suggesting that factors other than glucose were involved in the growth inhibition. Also, the timing and severity of necrotic cell death were well correlated with a drop in the ATP/ADP ratio (Figure 2G), implying that severe energy depletion triggered necrotic cell death. These dying cells were negative for the following markers (caspase 3 cleavage and PARP cleavage for apoptosis; LC3B transition for autophagic cell death; senescence-associated β-galactosidase for cellular senescence; and PAR accumulation for parthanatos; Supplemental Figure 2, A–C). Despite the prominent potency toward tumor cells, PT had negligibly weak cytotoxicity toward EBV-free nontumor cell lines (Figure 1, E–G, and Figure 2, A, B, and D).

Overall, PT induced severe growth inhibition toward broad types of tumor cells. The tumor cells eventually underwent necrotic cell death due to ATP depletion and loss of plasma membrane integrity. At the same time, PT-treated tumor cells showed altered metabolism to upregulate glucose uptake and lactate production, and such cells were nonviable without glucose. Despite these prominent effects on tumor cells, PT had only minor effects on the nontumor cells.

### PT had remarkably higher inhibitory potency against ETCC1 than metformin and phenformin.

**Figure 3:**
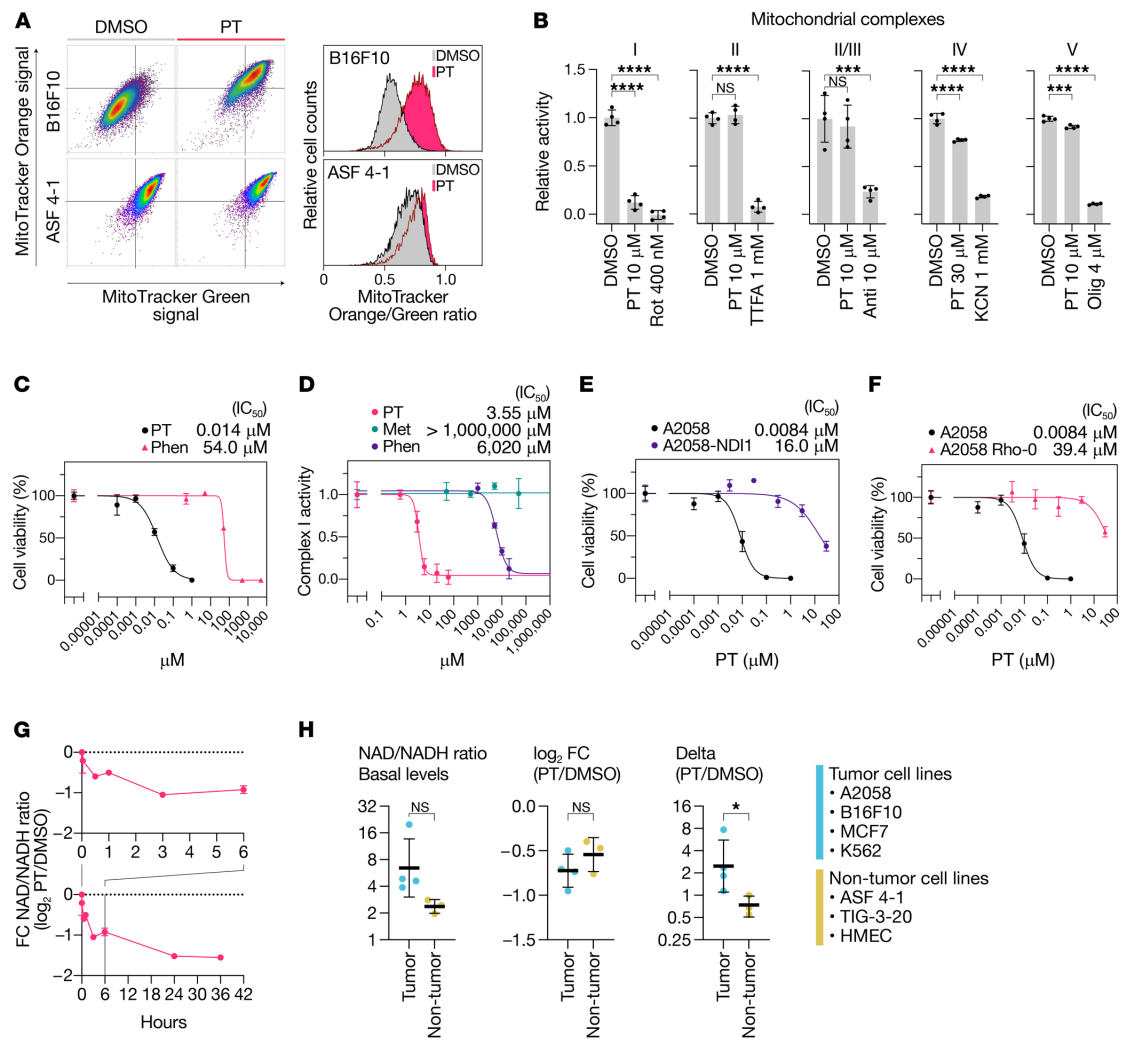
Petasin is a highly potent mitochondrial complex I inhibitor. (**A**) Scatter plots and histogram of B16F10 and ASF 4-1 cells stained with MitoTracker orange/green dyes. The cells were treated for 72 hours with petasin (PT, 3 μM) or DMSO. (**B**) Inhibitory activity of PT against mitochondrial electron transport chain complex I (ETCC1), II, II/III, IV, and V (ATP synthetase). Rot, rotenone; TTFA, thenoyltrifluoroacetone; anti, antimycin A; KCN, potassium cyanide; olig, oligomycin. ***P < 0.001, ****P < 0.0001, NS (not significant), 1-way ANOVA with Dunnett’s post hoc test (*n =* 4). (**C**) Dose-response curves for viable cell counts of B16F10 cells treated with PT or phenformin (Phen). (**D**) Inhibitory potencies of PT, metformin (Met), and Phen against ETCC1 of mitochondria isolated from bovine heart tissues. (**E**) Dose-response curves for viable cell counts of PT-treated A2058 cells with or without overexpression of *NDI1*, a yeast homolog of mammalian ETCC1 core subunit *ND1*. (**F**) Dose-response curves for viable cell counts of PT-treated A2058 cells lacking mitochondrial DNA and functional mitochondrial electron transport chain (A2058 Rho-0) or its WT cells. (**G**) Time-course change in NAD/NADH ratio fold-change (FC) in B16F10 cells treated with PT (3 μM) or DMSO. (**H**) Basal levels, FC, and delta of NAD/NADH ratio in tumor cells (B16F10, A2058, MCF7, and K562) and nontumor cells (ASF 4-1, TIG-3-20, and HMEC) treated with PT (3 μM) or DMSO. *P < 0.05; 2-tailed, unpaired Student’s *t* test unless otherwise indicated. Data are presented as the mean ± SD (*n =* 3) unless otherwise indicated. NS, not significant.

Since PT induced mitochondrial damage (Figure 2E) and ATP depletion (Figure 2G), we further examined the effects of PT on mitochondrial function. As expected, PT treatment affected the mitochondrial membrane potential (Figure 3A) and significantly inhibited ETCC1 activity (Figure 3B), whereas it had little effect on the other ETCs. Since these findings suggested that PT mainly worked as an ETCC1 inhibitor, we next sought to examine possible differences between PT and other conventional ETCC1 inhibitors, such as metformin and phenformin. As a result, we found that PT showed extremely higher cytotoxicity than phenformin, one of the most potent compounds among commercially available biguanides (3800 times lower than the IC_50_ of phenformin, Figure 3C). We further found that PT had at least approximately 1700 times higher ETCC1 inhibitory activity than phenformin and metformin (PT, IC_50_ = 3.55 μM; phenformin, IC_50_ = 6 mM; metformin, IC_50_ > 50 mM; Figure 3D).

To clarify whether high potency of PT toward ETCC1 was responsible for the antitumor activity, we assessed whether *NDI1*-mediated recovery of ETCC1 activity could revert the cytotoxicity against tumor cells. *NDI1* from budding yeast (*Saccharomyces cerevisiae*) is an NAD/NADH oxidoreductase with a different structure from that of the mammalian ETCC1; therefore, enforced expression of *NDI1* allows mammalian cells to recover the NAD/NADH oxidoreductase activity even under inhibition of endogenous ETCC1 activity (18). The result showed that *NDI1*-overexpressed A2058 cells lost sensitivity to PT treatment with approximately 1900 times higher IC_50_ than that of the original A2058 cells (Figure 3E). We also generated A2058 cells with depleted mitochondrial DNA (mtDNA), thus lacking functional ETC activity by using ethidium bromide (A2058 Rho-0), and examined the sensitivity of these cells to PT treatment. As expected, A2058 Rho-0 cells had markedly decreased sensitivity to the treatment (Figure 3F).

Since ETCC1 is an NAD/NADH oxidoreductase, we next sought to determine its effect on the cellular NAD/NADH ratio. Expectedly, PT significantly decreased the NAD/NADH ratio in all cell lines tested (Figure 3, G and H). PT treatment immediately decreased NAD/NADH ratio within 5 to 30 minutes and further kept decreasing it up to 36 hours (Figure 3G). Of note, PT uniformly decreased the NAD/NADH ratio in both tumor and nontumor cell lines (Figure 3H), although it exerted the cytotoxicity specifically to tumor cells. Given that tumor cell lines had higher basal NAD/NADH ratios, these findings suggested that tumor cells had high dependency on NAD metabolism and that PT targeted the NAD dependency to exert its cytotoxicity. Collectively, these findings indicated that PT had markedly higher inhibitory potency against ETCC1 and that this higher potency led to severe cytotoxicity toward tumor cells.

### PT disrupted NAD production and energy metabolism of tumor cells.

ETCC1 is the primary provider of NAD, an essential coenzyme driving 2 fundamental metabolic pathways, glycolysis and the TCA cycle; therefore, PT-mediated inhibition of ETCC1 could have a profound impact on cellular metabolism. Therefore, we further investigated the effects of PT on cancer metabolism.

**Figure 4:**
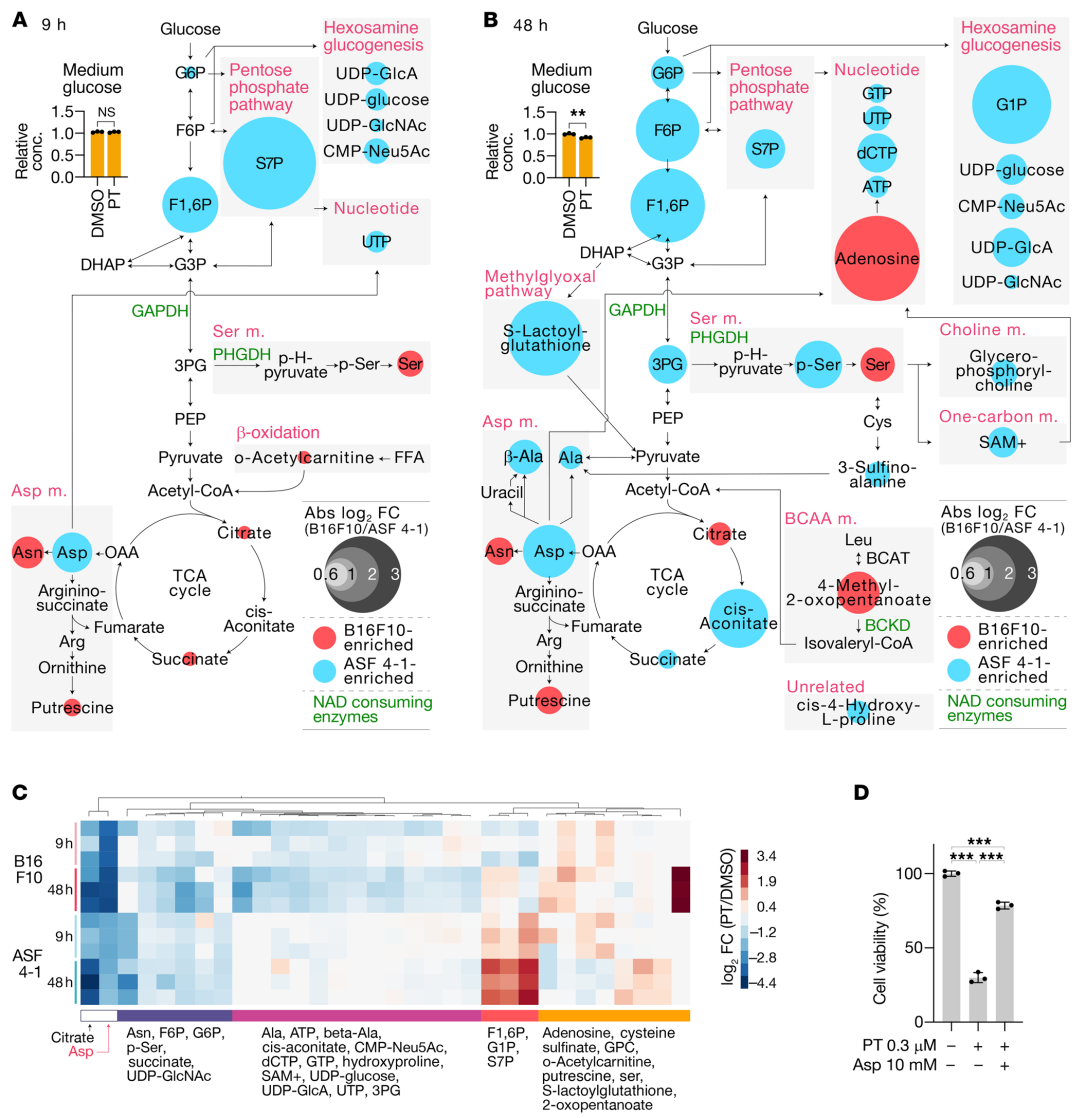
Petasin disrupts tumor-associated metabolism. (**A** and **B**) Pathway maps illustrating significantly different metabolites between tumor (B16F10) and nontumor (ASF 4-1) cells treated for 9 (**A**) or 48 (**B**) hours with petasin (PT, 3 μM) or DMSO. Altered metabolites are illustrated with colors (red, high in tumor cells; blue, low in tumor cells) and size of circles (degree of difference between tumor and nontumor cells; abs log_2_ FC, absolute log_2_ fold changes). NAD-consuming enzymes are marked as green. The bar graph in the map shows the glucose concentration in the medium of B16F10 cultures treated with 3 μM PT or DMSO for 9 or 48 hours. **P < 0.01, NS (not significant); 2-tailed, unpaired Student’s *t* test (*n =* 3). (**C**) Cluster analysis (Ward’s method) and heatmap showing significantly different metabolites in B16F10 and ASF 4-1 cells treated for 9 or 48 hours with PT (3 μM) or DMSO. (**D**) Cell viability percentages of B16F10 cells treated for 48 hours with PT alone (0.3 μM) or PT (0.3 μM) and aspartate (Asp, 10 mM). EMEM was used for the assay. ***P < 0.001, 1-way ANOVA with Tukey’s post hoc test. Metabolites with abs log_2_ FC greater than 0.585 and *P* values of less than 0.05 were included in the pathway map and heatmap. These assays were performed with high-glucose DMEM supplemented with 10% FBS unless otherwise indicated. Data are presented as the mean ± SD (*n =* 3).

Firstly, we assessed the metabolic differences between tumor and nontumor cells under PT treatment (B16F10 cells and ASF 4-1 cells, respectively). As expected, PT treatment prominently decreased the levels of TCA cycle–associated and glycolysis-associated metabolites in B16F10 cells (Figure 4, A–C and Supplemental Figure 3). Aspartate metabolism was one of the most primarily and severely affected pathways (Figure 4A), and the aspartate level was decreased up to approximately 6.8% of its steady-state one (Figure 4C). This depletion was evident at 9 hours and persisted for at least 48 hours. These results agree with several earlier studies reporting that aspartate synthesis is highly dependent on ETCC1 activity and TCA-cycle anaplerosis (19–21). Also, aspartate supplementation recovered the viable cell number in PT-treated tumor cells to near normal (Figure 4D), suggesting that aspartate depletion was responsible for the growth inhibition.

Another pathway primarily affected by PT treatment was glycolysis and its related pathways (Figure 4, A–C). The first phase of glycolysis (the investment phase) was particularly affected (fructose 1,6-bisphosphate [F1,6P], G6P; Figure 4A), which provides glucose-derived intermediates to the pentose phosphate pathway (PPP) and hexosamine pathway. In fact, the levels of both PPP and hexosamine pathway metabolites were also significantly decreased by PT treatment (S7P, UDP-glucose, CMP-Neu5Ac, UDP-GlcA, UDP-GlcNAc; Figure 4, A and C). The affected metabolic pathways were then further extended to their downstream pathways by 48 hours (Figure 4, B and C). These results suggested that PT treatment inhibited the hexosamine pathway–associated metabolism and hindered glycosylation, which is necessary for proper folding, stabilization, and function of proteins. It is noteworthy that these changes were observed under a relatively glucose-rich condition (4.5 g/L, high-glucose DMEM), indicating that these metabolic alterations were not simply caused by glucose deprivation. Rather, given that PT-treated B16F10 cells had accelerated glucose uptake and lactate production (Figure 2D), these metabolic alterations were likely due to altered metabolic flow to discard most of the glucose-derived intermediates as lactate. These data suggested that PT treatment made glycolytic metabolism quite inefficient, thus hampering tumor cells to produce a sufficient amount of cellular components.

Among these altered pathways, aspartate metabolism, PPP, and 1-carbon metabolism eventually flow into nucleotide synthesis; hence, their inhibition could severely hinder cell replication. In fact, the nucleotides (UTP, GTP, dCTP, ATP) and their precursors (S7P, aspartate, citrate, SAM^+^; Figure 4, A–C) were significantly downregulated in PT-treated B16F10 cells. These findings were also consistent with our finding that supplementation with aspartate, the most depleted metabolite in these pathways, rescued the PT-mediated growth inhibition (Figure 4D).

Several metabolites were relatively upregulated in PT-treated B16F10 cells (serine, asparagine, putrescine, and 4-methyl-2-oxopentanoate); however, the levels of their downstream metabolites were not apparently changed, suggesting that these upregulated metabolites were not well utilized in tumor cells under PT treatment (Figure 4, A–C). In spite of the prominent effects on the metabolism of tumor cells, PT-treated nontumor ASF 4-1 cells showed only minor downregulation or even upregulation of the metabolites, indicating that PT targeted the metabolism in a relatively tumor-specific manner. The patterns of the altered metabolites and pathways were consistent with reported metabolic pathways altered specifically in tumor cells (6, 22); thus, these changes were likely a reflection of the metabolic differences between tumor and nontumor cells.

**Figure 5:**
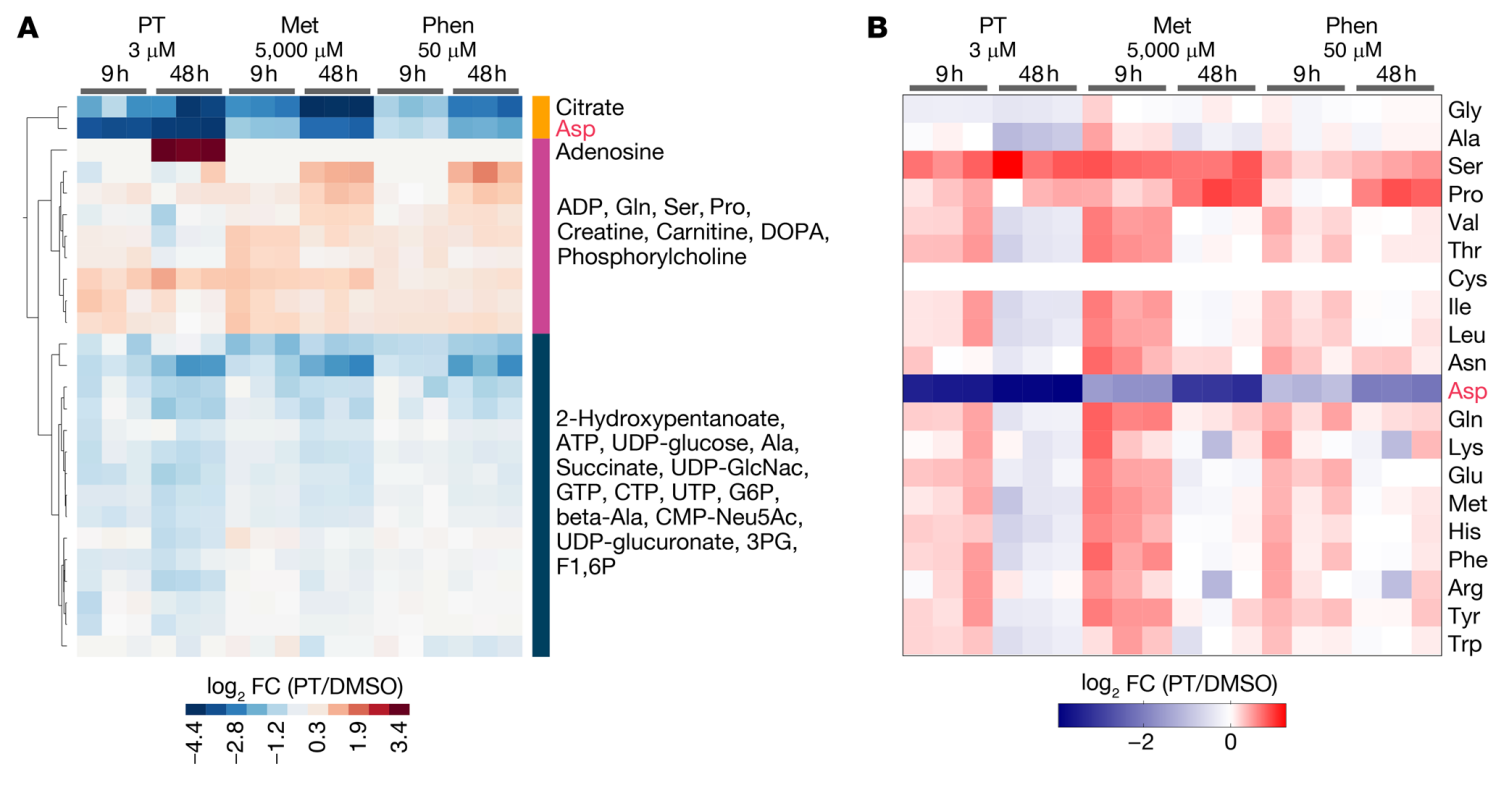
Petasin induces a similar metabolome profile to that of biguanides. (**A**) Cluster analysis (Ward’s method) and heatmap showing significantly different metabolites in B16F10 cells treated for 9 or 48 hours with petasin (PT, 3 μM), metformin (Met, 5000 μM), or phenformin (Phen, 50 μM). Metabolites with absolute log_2_ fold change (FC) greater than 0.585 and *P* values of less than 0.05 were included in the heatmap. (**B**) Heatmap showing FC in amino acid levels in B16F10 cells treated for 9 or 48 hours with PT, Met, or Phen. These assays were performed with high-glucose DMEM supplemented with 10% FBS.

Next, we sought to examine the difference between PT and biguanides regarding their effects on metabolism. PT has a completely different chemical structure from biguanides (Supplemental Figure 4); however, our results showed that PT induced a considerably similar metabolome profile with that of high-dose biguanides (Figure 5, A and B). However, the biguanides required a much higher concentration to induce the same degree of effect as PT (metformin, 5000 μM; phenformin, 50 μM). Only PT could decrease the overall amino acid levels at 48 hours (Figure 5B), likely reflecting its high potency. These findings suggested that PT and biguanides shared similar inhibitory mechanisms on the metabolism of tumor cells, despite their completely different chemical structures and potencies.

### PT upregulated ATF4 signals associated with amino acid depletion and unfolded protein stress in the ER.

**Figure 6:**
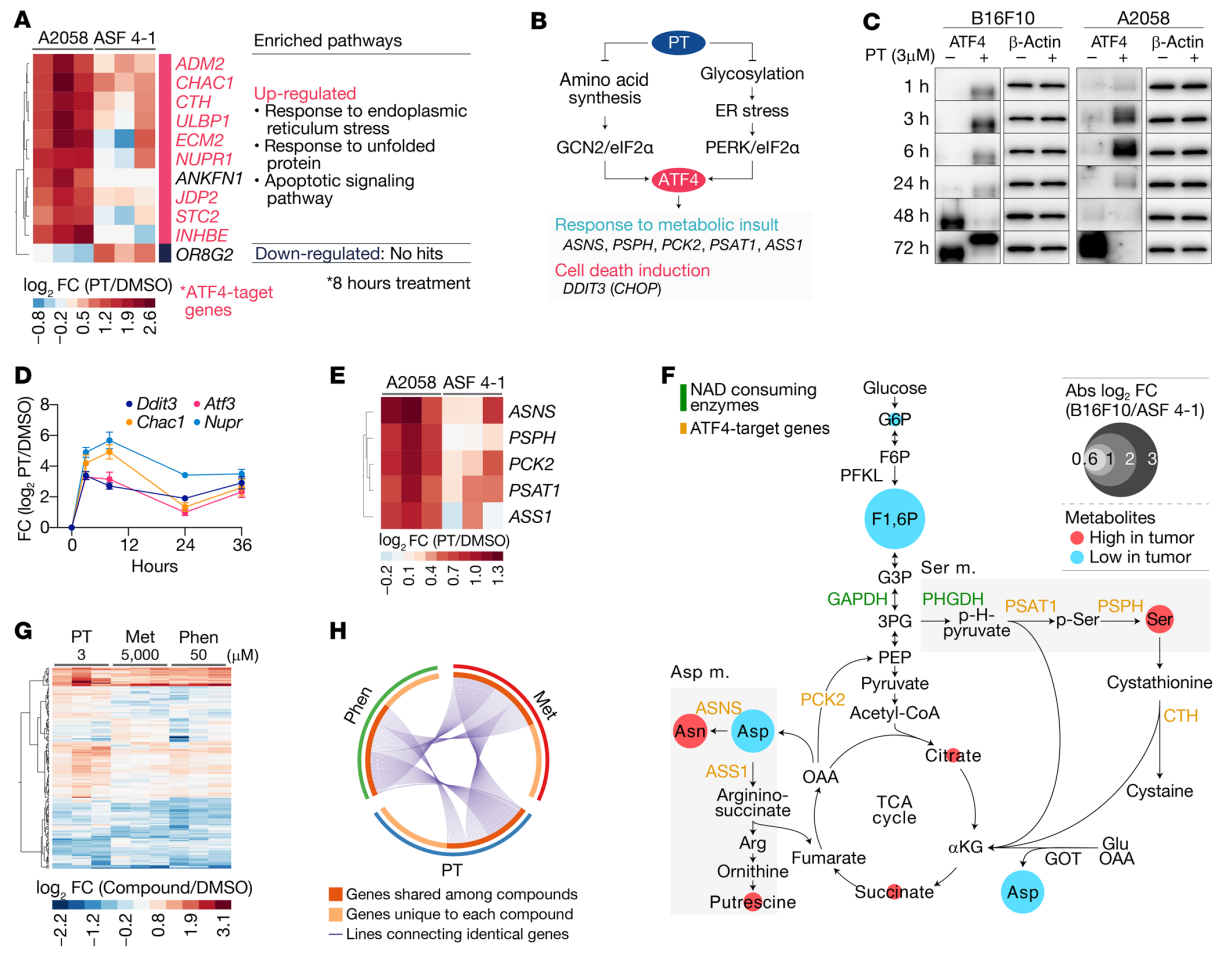
Petasin upregulates ER stress/unfolded protein response signals. (**A**) Genes and pathways significantly different between tumor (A2058) and nontumor (ASF 4-1) cells treated for 8 hours with PT (3 μM). Most of the differentially expressed genes (DEGs) were ATF4-target genes (marked as red). Metascape was used for the enrichment analysis to determine significantly different pathways (KEGG/Reactome pathways; FDR ≤ 0.01). (**B**) Schematic diagram illustrating ATF4-mediated signals in response to the unfolded protein/ER stress and amino acid depletion. (**C**) Time-course change in ATF4 expression in B16F10 or A2058 cells treated with PT (3 μM) or DMSO (loading control, β-actin). The data were obtained from the same membrane for comparison between different durations of treatment (intact images, Supplemental Figure 5). (**D**) Time-course changes in ATF4-regulated genes in B16F10 cells treated with PT (3 μM). (**E**) Differential expression of ATF4-regulated metabolic enzymes in A2058 and ASF 4-1 cells treated with PT (3 μM). (**F**) Integrated pathway map illustrating metabolites and transcripts altered by PT treatment (3 μM). Altered metabolites are illustrated with colors (red, high in tumor cells; blue, low in tumor cells) and size of circles (degree of difference between tumor and nontumor cells). Enzyme names are colored depending on their properties (orange, ATF4-regulated metabolic enzymes; green, NAD-consuming enzymes). (**G** and **H**) DEGs (**G**) and their Circos plot (**H**) for A2058 cells treated for 8 hours with PT (3 μM), metformin (Met, 5000 μM), or phenformin (Phen, 50 μM). The Circos plot illustrates DEG overlap between cells treated with each agent. The heatmaps were illustrated by cluster analysis (Ward’s method). DEGs were defined as follows: absolute log_2_ fold changes ≥ 1 for **A** or 0.585 for **G**, *P* values ≤ 0.05; 2-tailed, paired Student’s *t* test (*n =* 3).

To further investigate the effects of PT on the transcriptome of tumor cells, we performed cDNA microarray analysis. Most of genes altered in PT-treated tumor cells were ATF4-regulated genes (Figure 6A). ATF4 is a transcriptional factor typically upregulated by GCN2 or PERK in response to amino acid depletion or inhibition of protein glycosylation/folding processes in the ER (Figure 6B); therefore, the ATF4 upregulation was likely due to PT-induced metabolic insults. In fact, the timing and intensity were well correlated with the metabolic changes; i.e., ATF4 protein and its regulated genes were immediately upregulated within a few hours, and this upregulation persisted for approximately 36 hours (Figure 6, C and D, and Supplemental Figure 5). Also, PT treatment transcriptionally upregulated a group of ATF4-regulated enzymes associated with serine (*PSPH*, *PSAT1*), asparagine (*ASNS*), and arginine (*ASS1*) metabolism. These transcriptional changes were highly correlated with the increased levels of serine, asparagine, and putrescine in PT-treated tumor cells (Figure 6F). On the other hand, the nontumor cells showed weak ATF4 signals in response to PT treatment, reflecting that these cells had only slight metabolic changes (Figure 6, A and F). Similar to the result of metabolome analysis, PT shared quite similar mRNA profiles with biguanides (metformin and phenformin), although the biguanides required a much higher concentration to provoke similar changes (Figure 6, G and H). Collectively, PT treatment upregulated ATF4 signals, likely reflecting severe amino acid depletion and unfolded protein stress in the ER.

### PT induced downregulation of oncoproteins.

**Figure 7:**
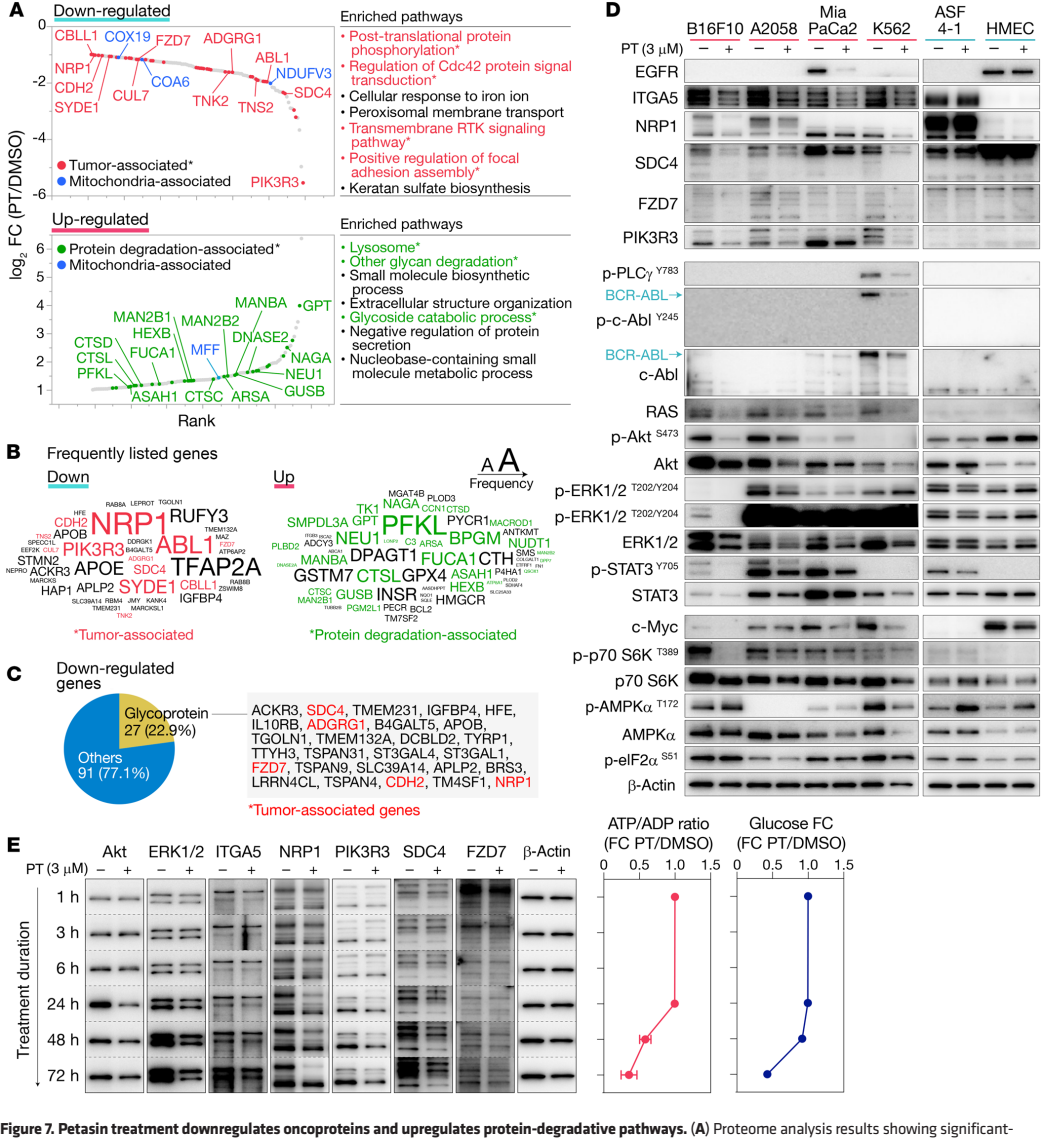
Petasin treatment downregulates oncoproteins and upregulates protein-degradative pathways. (**A**) Proteome analysis results showing significantly altered proteins (absolute log_2_ fold change ≥ 1, *P ≤* 0.05; 2-tailed, unpaired Student’s *t* test, *n =* 2) and pathways (top 7 pathways with *P ≤* 0.001, Metascape enrichment analysis using KEGG or Reactome pathways) in B16F10 cells treated for 72 hours with PT (3 μM). Genes and pathways are marked in color depending on their properties (red, tumor associated; blue, mitochondria associated; green, protein degradation associated). The downregulated proteins or pathways were mainly associated with proliferation or metastasis, whereas upregulated ones were associated with protein degradation. (**B**) Frequently listed genes in the altered pathways. The font size indicates the frequency that each gene appeared in the pathways, with larger size indicating greater frequency. Genes are marked with color depending on their properties (red, tumor associated; green, protein degradation associated). (**C**) Percentage of glycoprotein-encoding genes among the downregulated genes. Tumor-associated genes are marked in red. (**D**) Immunoblots for oncoproteins and metabolism-related proteins in melanoma (B16F10, A2058), pancreatic cancer (MiaPaCa2), chronic myeloid leukemia (K562), and nontumor (ASF 4-1 and HMEC) cell lines (loading control: β-actin). Glycosylation levels of glycoproteins were significantly reduced in tumor cell lines but not in nontumor cell lines. (**E**) Time-course changes in oncoproteins, ATP/ADP ratio, and medium glucose concentration in B16F10 cells treated with PT (3 μM) or DMSO. The downregulation of oncoproteins started before the drops in the ATP/ADP ratio and medium glucose concentration. The data were obtained from the same membrane for each target for comparison between different durations of treatment (intact images, Supplemental Figure 6C). High-glucose DMEM supplemented with 10% FBS was used for the assays. Data are presented as the mean ± SD (*n =* 3) unless otherwise indicated.

PT-mediated metabolic alterations in tumor cells may affect the synthesis, folding, and function of proteins; thus, we performed proteome and immunoblot analysis to assess its impact on the protein turnover and modification. The results showed that PT treatment drastically decreased total/phosphorylated levels of numerous proteins associated with growth, cell motility, invasion, and metastasis (Figure 7, A–E). The downregulated proteins were mainly associated with protein phosphorylation, CDC42 protein signal transduction, the receptor tyrosine kinase (RTK) signaling pathway, and focal adhesion assembly (Figure 7, A–D), all of which play essential roles in tumor growth and metastasis. Of note, not a few glycoproteins were listed as downregulated proteins (Figure 7C), and their glycosylated forms were markedly downregulated by PT treatment (NRP1, SDC4, ITGA5; Figure 7, D and E). Also, the oncoproteins that rely on the hexosamine pathway for its proper expression and function, such as c-Myc, EGFR, and Akt (23, 24), were also downregulated by PT treatment (Figure 7D). These data suggested that the decreased levels of metabolites in the hexosamine pathway negatively affected the stability and folding of oncoproteins. Also, the downregulation of oncoproteins was already evident within 24 hours before the ATP/ADP ratio drop (Figure 7E), indicating that these oncoproteins were downregulated at an early stage during PT treatment. The downregulation of oncoproteins was evident in all human/mouse tumor cell lines tested, although these changes were not apparent in the nontumor cell lines (Figure 7D).

On the other hand, PT treatment upregulated protein/glycoprotein degradation pathways, such as lysosome, glycan degradation, and glycoside catabolic processes (Figure 7A). Indeed, PT-treated B16F10 cells had increased levels of glycosidases (NEU1, FUCA1, MANBA, NAGA, GUSB, MAN2B1, HEXB), proteases (CTSL, CTSC), ceramidase (ASAH1), and sulfatase (ARSA), along with the compensative upregulation of GPT and PFKL for replenishing depleted aspartate and F1,6P, respectively (Figure 7, A and B). These results suggested that PT treatment attenuated the stability of the oncoproteins and accelerated their degradation.

Since ETCC1 inhibitors are typically noted as exerting their antitumor activity through the AMPK/mTOR–mediated pathways (19), we assessed the involvement of these signals in the PT-mediated oncoprotein downregulation. We unexpectedly found that AMPK/mTOR signals were not well correlated with the pattern or degree of the PT-induced oncoprotein downregulation (Supplemental Figure 6, A and B). Although PT treatment induced weak and transient AMPK signals in tumor cells, these signals were unchanged or even downregulated at the late stage (Supplemental Figure 6, A and B). Also, the downregulation of mTOR-regulated phosphorylated p70 S6K (p-S6K) was not clearly observed in the cell lines examined, except for one cell line (B16F10, Figure 7D). The timing and strength of these AMPK/mTOR signals were not well correlated with those of oncoprotein downregulation (Supplemental Figure 6, A and B). Particularly, PT-treated A2058 cells had prominent downregulation of oncoproteins, even though these cells had negligibly weak p-AMPKα and p-S6K signals (Figure 7D and Supplemental Figure 6, A and B).

### PT inhibited tumor growth in vivo.

**Figure 8:**
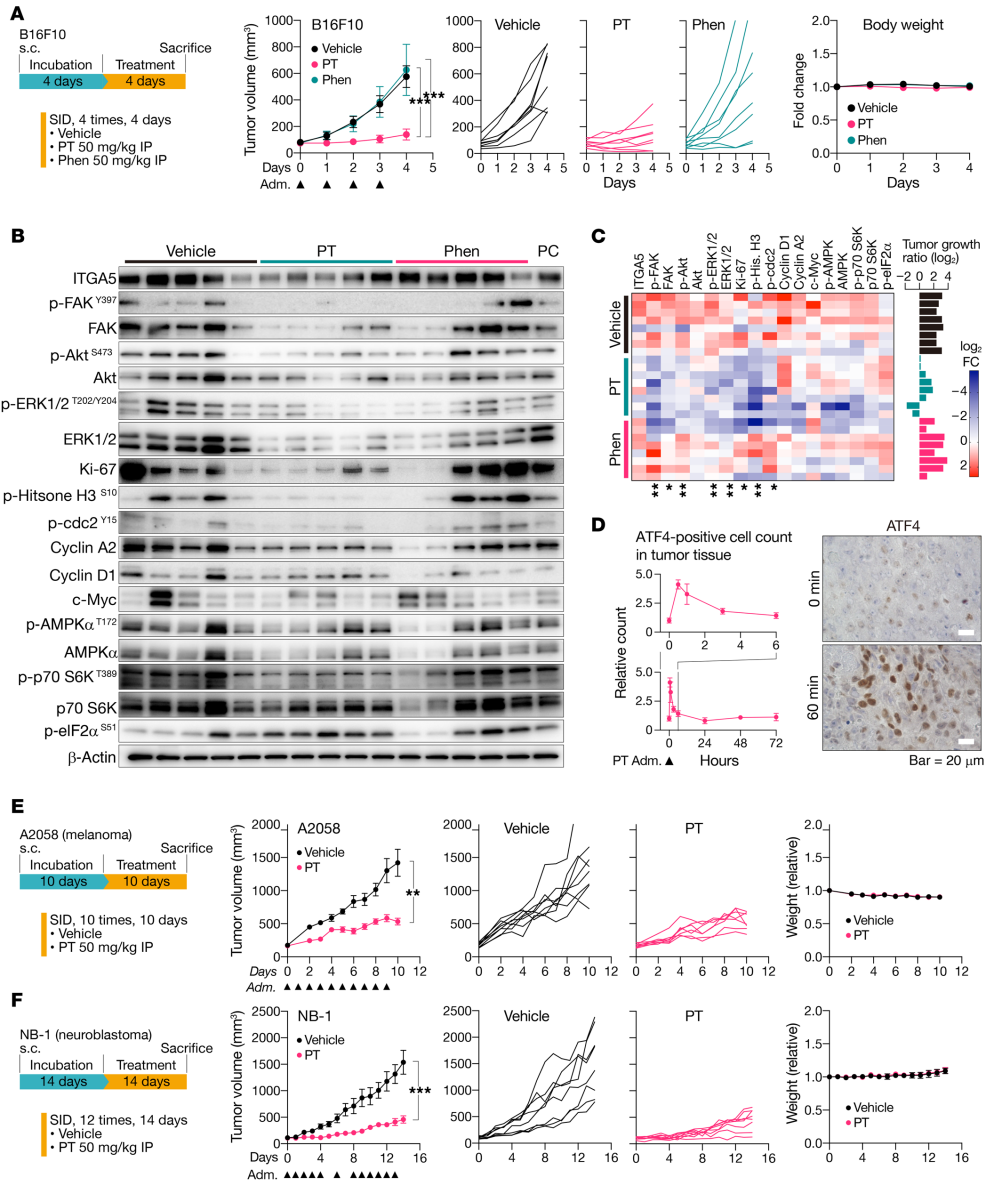
Petasin inhibits tumor growth in multiple human xenograft and mouse syngeneic models. (**A**) Experimental protocol, tumor volume, and body weight in the experiments to determine the antiproliferative effect of petasin (PT) in the orthotopic B16F10 syngeneic mouse model (*n =* 8). Triangles under the *x* axis indicate the timing of administration (adm). ***P < 0.001, 1-way ANOVA with Tukey’s post hoc test. (**B**) Representative immunoblots for oncogenes, cell cycle–related proteins, and metabolism-related proteins in the tumor samples from **A**. A pooled control (PC) was used for normalizing signals in different membranes (full data, Supplemental Figure 7). (**C**) Heatmap summarizing the protein levels (quantified by densitometry, normalized with the β-actin signals) with bar graphs showing the tumor growth rate of each mouse. *P < 0.05, **P < 0.01, Kruskal-Wallis test. Tumor growth rate = (final tumor volume)/(tumor volume at treatment start). (**D**) Time-course change in ATF4 (amino acid depletion/unfolded protein response marker) expression in tumor tissues. Mice bearing B16F10 subcutaneous masses received a single injection of PT (50 mg/kg, i.p.) and were euthanized at various time points (0, 0.5, 1, 3, 6, 24, 48, 72 hours; *n =* 3 per time point). The tumor tissue was immunohistochemically stained with anti-ATF4 antibody to count the number of ATF4-positive cells (representative images for 0- and 60-minute time points; scale bar: 20 μm). Spike in the graph after administration indicates successful drug delivery to the tumor tissues. (**E** and **F**) Experimental protocol, tumor volume, and body weight of the experiments designed to determine the antiproliferative effect of PT in the human subcutaneous xenograft model using A2058 (glycolytic, **E**) and NB-1 (glycolysis-impaired, **F**) cells (*n =* 8). SID, once per day. Data are presented as the mean ± SEM (**A**, **E**, and **F**) or SD (**D**). Triangles under the *x* axis indicate the timing of administration (adm). **P < 0.01, ***P < 0.001; 2-tailed, unpaired Student’s *t* test. Vehicle: PBS containing 1% v/v DMSO and 10% v/v high-purity oleic acid.

Because PT showed prominent growth inhibitory effects in vitro, we next evaluated its effects on in vivo tumor models. Firstly, we assessed its efficacy and side effects by using an orthotopic B16F10 melanoma model (Figure 8A). This model has the glycolytic feature as in most human cancers, in addition to the aggressive proliferative rate in vivo; thus, it is a useful model for evaluating the in vivo efficacy and molecular effects in the short term. Mice bearing a B16F10 subcutaneous mass were i.p. administered 50 mg/kg PT, phenformin, or vehicle once a day for 4 days (Figure 8A). The result showed that PT significantly inhibited B16F10 tumor growth, whereas phenformin at the same dose failed to inhibit the tumor growth (Figure 8B). Immunoblot and immunohistochemical analyses showed that PT treatment downregulated the oncoproteins associated with tumor growth and metastasis in the tumor tissues (Figure 8, B and C, Supplemental Figure 7, A and B, and Supplemental Figure 8, A and B). The time-course evaluation showed that PT administration immediately induced ATF4 upregulation in the tumor tissues within several hours (Figure 8D), suggesting that PT had been successfully delivered to the tumor tissue and induced amino acid depletion or inhibited glycosylation in vivo. PT treatment induced no apparent AMPKα activation, as was the case in the in vitro experiments but affected p-S6K and p-eIF2α levels (Figure 8, B and C), suggesting that inhibition of transcription and protein synthesis were also involved in the antitumor effects on the B16F10 in vivo model. Phenformin also elicited similar molecular profiles in the tumor tissues but with milder changes (Figure 8, B and C). Similarly, PT also exerted significant growth inhibition against 2 independent human cancer xenograft models having different metabolic backgrounds (melanoma, A2058 with complete glycolytic activity; neuroblastoma, NB-1 with deletion of a glycolytic enzyme, PGD; Figure 8, E and F). Of note, PT exhibited higher efficacy in the glycolysis-impaired NB-1 model than in the A2058 model with complete glycolytic activity (Figure 8, E and F), suggesting that PT induced its antitumor effects by inhibiting glucose metabolism. Immunohistochemical and immunoblotting analyses showed that PT treatment downregulated cell cycle–related proteins (p-histone H3 in A2058 and NB-1 models; cyclin D1 in the NB-1 model), glycoprotein (NRP1 in the A2058 model), and focal adhesion-associated protein (p-FAK^Y397^ in the NB-1 model) in the tumor tissues (Supplemental Figures 9 and 10), although the other proteins investigated were not significantly different.

Severe toxicity due to ETCC1 inhibition may be seen within a week as a severe weight loss (19); however, relatively intense PT administration for 2 weeks was well tolerated in these mice (Figure 8, A, E, and F and Supplemental Figures 11–14). The mice had neither severe weight loss nor apparent abnormalities in terms of blood cell count, blood biochemistry, histopathology of normal organs, and Ki-67 intensities in proliferative normal organs (intestine and bone marrow), suggesting that PT administration showed only minor toxicity toward normal organs at least for 2 weeks.

### PT inhibited migration and invasion of tumor cells in vitro.

**Figure 9:**
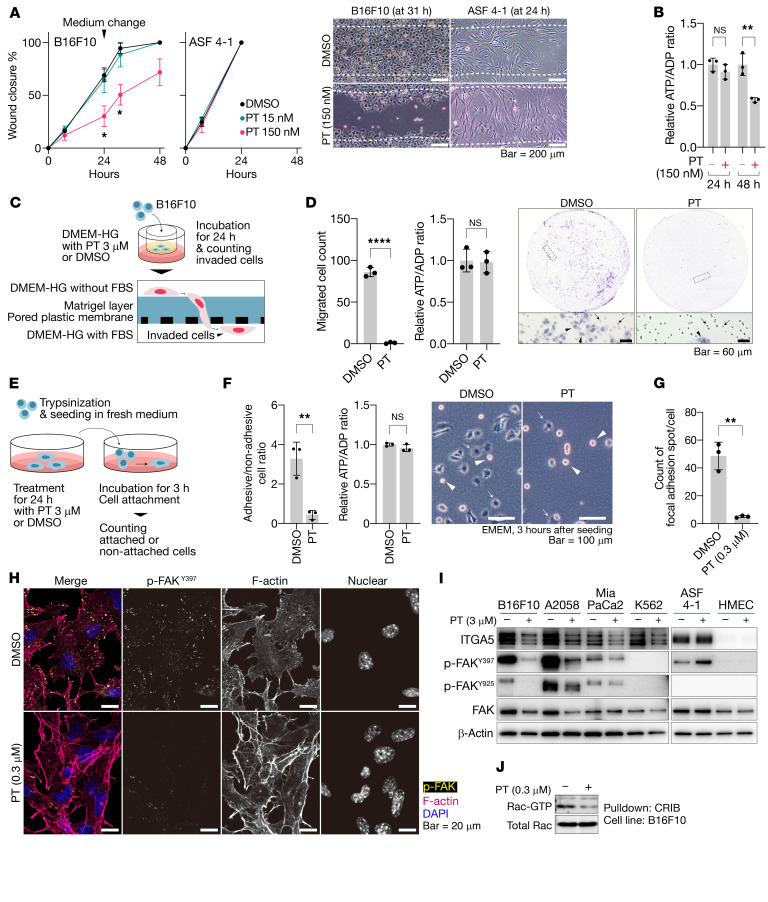
Petasin inhibits cellular motility and invasion of tumor cells. (**A**) Time-course wound closure percentage and representative images in the scratch wound healing assay for tumor (B16F10) and nontumor (ASF 4-1) cells treated with different concentrations of petasin (PT; 0, 15, or 150 nM). White dotted lines, wound borders at 0 hours. Scale bar: 200 μm. (**B**) ATP/ADP ratio of B16F10 cells treated for 24 or 48 hours with PT (150 nM). Note that cell migration was already inhibited within 24 hours before the drop in the ATP/ADP ratio. (**C**) Experimental protocol for the Matrigel invasion assay. (**D**) Representative images and counts for cells that invaded the Matrigel and passed through the PET membrane in **C** (arrow, cells that invaded; arrowhead, pores of the PET membrane). (**E**) Experimental protocol for the cell attachment assay. (**F**) Representative images, counts for cells that attached to the surface of the culture plate within 3 hours (arrow, attached cells; arrowhead, nonattached cells; scale bar: 100 μm), and the ATP/ADP ratio in **E**. (**G** and **H**) Counts of focal adhesion spots (**G**) and representative confocal microscopic images of immunofluorescence staining for p-FAK^Y397^ (yellow in the merged image), F-actin (phalloidin, red), and nuclei (DAPI, blue, **H**) in B16F10 cells treated with PT (0.3 μM) or DMSO (scale bar: 20 μm). (**I**) Immunoblot for focal adhesion-associated markers in tumor and nontumor cells treated with PT (3 μM) or DMSO (loading control, β-actin). (**J**) Pulldown assay for detecting the active form of Rac (Rac-GTP) in B16F10 cells treated for 48 hours with PT (0.3 μM) or DMSO in DMEM supplemented with 10% FBS. Data are presented as the mean ± SD (*n =* 3). *P < 0.05, **P < 0.01, ****P < 0.0001; 2-tailed, unpaired Student’s *t* test. NS, not significant High-glucose DMEM was used for the assays unless otherwise indicated.

We next assessed whether PT treatment could inhibit cell motility, invasion, and focal adhesion in vitro. The results showed that PT-treated B16F10 cells had significantly attenuated cell movement in the scratch wound healing assay (Figure 9, A and B). Also, PT treatment decreased the number of B16F10 cells that degraded Matrigel and invaded toward chemotaxis signals (Figure 9, C and D). Furthermore, the PT-treated tumor cells took a longer time to attach to the surface of culture dishes when the cells were reseeded into a new culture dish (Figure 9, E and F). However, PT treatment had no significant effect on the nontumor ASF 4-1 cells in the scratch wound healing assay (Figure 9A). Notably, significant inhibition of cell motility and invasion was already evident within 24 hours before the drop in the ATP/ADP ratio (Figure 9, B, D, F). Given that the downregulation of oncoproteins was already evident at 24 hours, these data suggested that downregulation of oncoproteins was also involved in the inhibitory mechanism of cell motility and invasion. Consistent with above findings, immunofluorescence and confocal microscopic analyses revealed that PT treatment induced drastic cytoskeletal remodeling with a loss of focal adhesion sites (Figure 9, G and H). Also, PT treatment downregulated protein levels of ITGA5 (integrin A5), FAKs phosphorylated at Y397 (an autophosphorylated site in association with integrin), and Y925 (c-Src interaction sites) (Figure 9I). Along with these changes, PT treatment downregulated the active form of Rac (Rac-GTP), an essential regulator of tumor invasion through cytoskeletal remodeling (Figure 9J). All these findings indicated that PT had prominent inhibitory activities toward cell motility, invasion, and focal adhesion formation in vitro before the depletion of ATP.

### PT inhibited metastasis in vivo.

**Figure 10:**
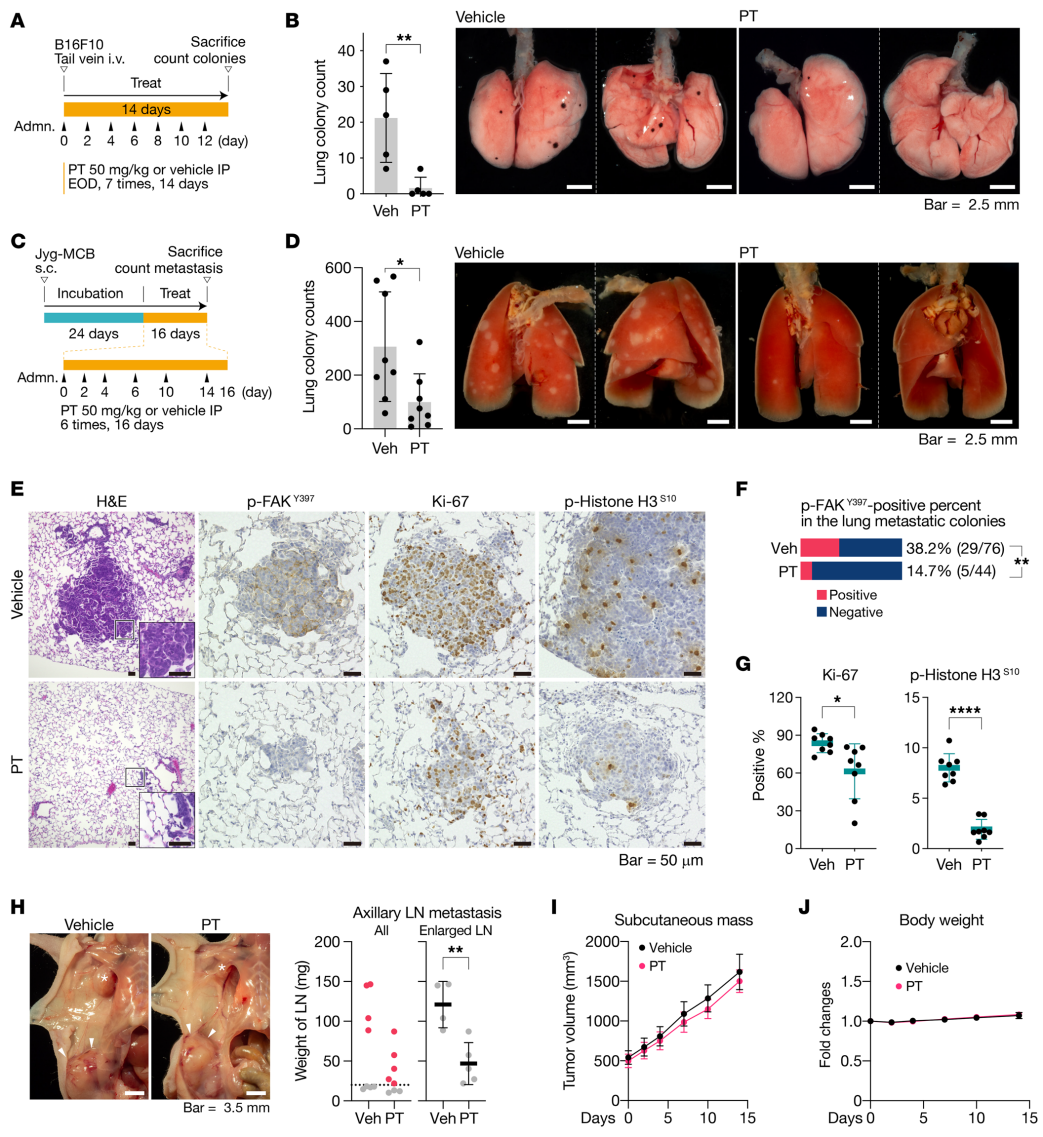
Petasin inhibits metastasis in vivo. (**A**) Experimental protocol for the experiment designed to determine the inhibitory activity of petasin (PT) against lung colonization of i.v.-injected B16F10 cells. EOD, every other day. (**B**) Counts and representative images of lung colonies in the mice from **A** (*n =* 5; all lung images, Supplemental Figure 15; scale bar: 2.5 mm). (**C**) Experimental protocol for the experiment used to determine the inhibitory activity of PT against lung and lymph node (LN) metastasis in a spontaneous metastatic model using Jyg-MCB cells. (**D**) Counts and representative images of lung metastasis in the mice from **C** (*n =* 8). The lungs were fixed by infusing neutral buffered 4% paraformaldehyde into the cannulated trachea to visualize the metastatic spots (all lung images, Supplemental Figure 16A; scale bar: 2.5 mm). (**E**) Representative images of H&E and immunohistochemical staining (p-FAK^Y397^, Ki-67, and p-Histone H3^S10^) for the lung tissue of mice in **C**. Scale bar: 50 μm. (**F**) Percentages of p-FAK^Y397^–positive lung metastatic colonies of mice in **C**. **P < 0.01, 1-way ANOVA with Fisher’s exact test. (**G**) Count for Ki-67– or p-Histone H3^S10^–positive proliferating cells in the lung metastatic tissues (*n =* 8). (**H**) Representative images and weight of axillary LNs in **C** (asterisk, enlarged axillary LNs; arrowheads, primary tumors; scale bar: 3.5 mm). Red dots, weight of enlarged LNs; dashed line, the threshold for LN enlargement. (**I**) Growth curves for primary subcutaneous Jyg-MCB tumor (*n =* 8). (**J**) Body weight of mice in **C** (*n =* 8). Vehicle (Veh): PBS containing 1% v/v DMSO and 10% v/v high-purity oleic acid. Triangles under the orange bar in the schematic diagrams indicate the timing of administration (adm). Data are presented as the mean ± SD (**B**, **D**, **G**, and **H**) or SEM (**I** and **J**). *P < 0.05, **P < 0.01, ****P < 0.0001; 2-tailed, unpaired Student’s *t* test. NS, not significant.

We further assessed the potency of PT to inhibit metastasis by using 2 in vivo metastatic models. Firstly, we utilized the lung colonization assay using B16F10 cells to examine whether PT could inhibit i.v.-injected B16F10 cells from forming colonies in the lung (Figure 10A). The results showed that PT administration (50 mg/kg i.p., every other day for 14 days) significantly decreased lung colony counts (Figure 10B and Supplemental Figure 15). Furthermore, we evaluated its antimetastatic potential in the Jyg-MCB (mouse metastatic mammary cancer) spontaneous metastatic model, in which mice developed lung and lymph node metastasis from the sites of the s.c.-injected cells. Mice were s.c. injected with Jyg-MCB cells and 24 days thereafter, they received i.p.-delivered PT at 50 mg/kg for a total of 6 times over a 16-day period (Figure 10C). As a result, we found that PT treatment also significantly inhibited metastasis to the lungs and lymph nodes (Figure 10, D–H and Supplemental Figure 16A). Immunohistochemical analysis revealed that the mice administered with PT had fewer p-FAK^Y397^–positive lung metastatic colonies, as well as a lower positive percentage of cell cycle–related proteins (Ki-67 and p-histone H3^S10^; Figure 10, E–G). Of interest, under this experimental condition, PT treatment showed no apparent growth-inhibitory effects on primary tumors despite the significant antimetastatic effects (Figure 10I), indicating that PT had higher efficacy to inhibit metastasis than the growth of primary tumors. The mice had neither severe weight loss nor apparent abnormalities of blood cell count, blood biochemistry, and Ki-67 intensities in proliferative normal organs (intestine and bone marrow; Figure 10J, Supplemental Figure 16B, and Supplemental Figure 17). Overall, these findings indicate that PT could inhibit metastasis to lungs and lymph nodes in vivo.

## Discussion

Here, we identified PT as a highly potent ETCC1 inhibitor with at least 1700 times higher activity than that of biguanides (metformin and phenformin). We demonstrated that PT showed prominent cytotoxicity toward a broad spectrum of tumor cell lines. PT-treated tumor cells showed significantly attenuated proliferation, motility, and invasion activities, eventually resulting in necrotic cell death with ATP depletion. Such prominent cytotoxicity was due to the exceptionally high inhibitory potency against ETCC1. The PT-mediated ETCC1 inhibition disrupted the TCA cycle–associated and glycolysis-associated metabolism, leading to severe impairment of nucleotide synthesis and glycosylation. Furthermore, oncoproteins associated with aggressive proliferation and metastasis were drastically downregulated in the PT-treated cells. Such antigrowth/antimetastatic activities were also evident in multiple human xenograft and mouse syngeneic models. Despite the prominent tumor inhibitory activities, PT had only minor effects on the nontumor cells and normal organs. These findings suggest that PT has promising potential as a potent ETCC1 inhibitor for cancer treatment through disruption of cancer cell metabolism.

Although PT exerted prominent cytotoxicity and metabolic disruption in the tumor cells, it induced only minor changes in the nontumor cells. The tumor specificity may be explained by the high dependency of tumor cells on NAD metabolism. NAD is an essential cofactor to drive glycolysis and the TCA cycle (25), and tumor cells have a high demand for NAD for efficient synthesis of macromolecules that contribute to rapid proliferation and metastasis (25). Indeed, oncogenic RAS, c-Myc, and Akt signals are known to increase dependency on ETCC1 and NAD metabolism through tumorigenic processes (6, 26–28). Such tumor cells are highly sensitive to the NAD depletion strategy, whereas NAD depletion has only slight effects on nontumor cells (29). These reports agree with our findings that PT uniformly downregulated the NAD/NADH ratio in both tumor and nontumor cells, but only tumor cells showed high sensitivity to the treatment.

Several acute toxicities have been reported for other potent ETCC1 inhibitors. The well-established potent ETCC1 inhibitor rotenone has nonspecific interaction with microtubes and induces off-target toxicity, such as severe bone marrow suppression (13, 30). Also, the recently developed potent ETCC1 inhibitor IACS-010759 has relatively specific activity toward ETCC1 but induces severe weight loss at a high dosage (31). Both of these toxicities are typically evident within 1 week. In contrast, the intensive PT administration (once per day, i.p., 50 mg/kg) did not induce apparent toxicities in major organs at least for 2 weeks. Although the exact reason for the difference in the toxicities between PT and the other reported ETCC1 inhibitors is still elusive, these findings suggest that the toxicological features of PT are distinct from those of rotenone and IACS-010759; rather, they are similar to those of safer ETCC1 inhibitors, such as biguanides. This similarity is further supported by the findings that PT had metabolic and transcriptomic profiles similar to those of biguanides. These findings suggest that PT serves as an ETCC1 inhibitor with high potency and safety. Nevertheless, further study is necessary to validate its exact degree of safety, given the broad and profound functions of ETCC1.

Cellular ATP and NAD levels are cooperatively maintained by oxidative phosphorylation (OXPHOS) and anaerobic glycolysis. ETCC1 and subsequent OXPHOS are major sources of NAD and ATP; thus, inhibition of ETCC1 could cause shortages of ATP and NAD. Indeed, PT treatment increased glucose consumption and lactate production in tumor cells, suggesting that tumor cells upregulated anaerobic glycolysis to compensate the energy shortage. However, the anaerobic glycolysis is much less efficient for producing ATP than OXPHOS (glycolysis, 2 ATPs/glucose; OXPHOS, 30–36 ATPs/glucose); therefore, such metabolic changes force tumor cells to consume an extensive amount of glucose as compensation. Also, NAD is regenerated by lactate dehydrogenase in anaerobic glycolysis; however, this reaction does not increase the net amount of NAD since GAPDH consumes the same amount of NAD in the process. Therefore, the upregulation of anaerobic glycolysis would be insufficient to fully compensate the loss of ATP and NAD, and such changes could cause a shortage of glucose-derived metabolites.

Indeed, PT treatment caused downregulation of glucose-derived metabolites in 2 major branch pathways from glycolysis, the hexosamine biosynthetic pathway (HBP) and PPP. These 2 pathways are upregulated in tumor cells and contribute to the synthesis of more macromolecules for tumor growth and metastasis (22). Particularly, the HBP is indispensable for glycosylation, which promotes proper folding, function, and stabilization of proteins. A vast number of oncoproteins including c-Myc, Akt, and EGFR are regulated in a glycosylation-dependent manner (23, 24); therefore, downregulation of HBP and glycosylation could severely impair the turnover and function of oncoproteins. In fact, PT treatment induced extensive downregulation of oncoproteins with concomitant upregulation of protein-degradative pathways and stress of unfolded protein in the ER. These findings suggest that PT treatment disturbed the ETCC1-mediated metabolic flux and induced oncoprotein degradation in tumor cells.

The loss of NAD also caused severe depletion of aspartate, an indispensable metabolite for the synthesis of purine and pyrimidine nucleotides (21). The aspartate depletion, along with PPP inhibition, is highly likely a direct cause of the nucleotide depletion and subsequent severe inhibition of cell replication. This likelihood is further supported by our finding that supplementation with aspartate rescued the PT-induced growth inhibition. Such aspartate depletion strategy may be effective particularly for tumor cells in vivo, since aspartate availability is quite limited in the microenvironment because of the low permeability of the cell membrane to it (19, 20), its low blood level (21, 32), and the fact that the asparaginase activity necessary to convert asparagine to aspartate is absent in most mammals (33–35).

PT treatment significantly inhibited the formation of lung metastatic colonies in both spontaneous metastatic and i.v. injection models. This effect was likely due to ETCC1 inhibition and its subsequent events, including energy depletion, cytoskeletal remodeling, focal adhesion inhibition, oncoprotein downregulation, and cell-cycle arrest. Particularly, PT treatment induced depletion of ATP, an energy source for all metastatic steps, including invasion, extravasation, and colonization. Tumor cells flexibly utilize both glycolysis and OXPHOS for ATP production; however, PT treatment defunctionalized OXPHOS-mediated ATP production and made tumor cells highly dependent on glycolysis for their ATP production. Such tumor cells easily demonstrated ATP depletion under the low-glucose condition. Given that glucose is typically less available in the tumor microenvironment (36), the loss of flexibility for ATP production could critically impair the metastatic potential of the tumor cells in vivo.

PT treatment induced necrotic cell death, which could result in immunological reactions. Such immunogenicity may promote tumor pathology under some circumstances; however, immunogenic cell death also works for protecting against cancer by inducing long-lasting protective antitumor immunity (37). Furthermore, recent studies suggest that metformin, a conventional ETCC1 inhibitor, improves the anticancer effects of immune checkpoint inhibitors and thus has beneficial effects on cancer prevention and treatment (38). Given these reports, PT may induce immunogenic cell death in vivo; however, the immunogenicity could be exploited for improving efficacy when combined with immunotherapy.

In conclusion, we identified PT as a highly potent ETCC1 inhibitor. Given that PT-mediated ETCC1 inhibition simultaneously disrupted multiple metabolism essential for tumor growth and metastasis, such a strategy could provide a better chance to conquer metastatic tumors that have heterogeneous molecular backgrounds and thus are resistant to conventional single molecule–targeted therapy. Also, since PT has a unique chemical structure and high potency, it may serve as a lead compound to develop a novel family of potent and less toxic ETCC1 inhibitors for treating metastatic tumors.

## Methods

Please see Supplemental Methods for the following methods: development and screening of a phytochemical library; identification of active ingredients by use of HPLC; purification of PT; spheroid formation assay; cell-cycle analysis; measurement of glucose, lactate, and pH in culture medium; transmission electron microscopy; ATP/ADP ratio; senescence-associated β-galactosidase staining; MitoTracker orange/green staining; mitochondrial ETC activity assays; *NDI1* overexpression; generation of Rho-0 cells; NAD/NADH ratios; metabolome analysis; cDNA microarray analysis; proteome analysis; animal experiments; scratch wound healing assay; Matrigel invasion assay; cell attachment assay; and Rac1 pulldown assay.

### Cell lines and culture.

MDA-MB-231, WiDr, B16F10, and CMeC1 cells were cultured in DMEM (Wako) supplemented with 10% FBS (Sigma-Aldrich); NB-1 cells in high-glucose DMEM (Wako) supplemented with 20% FBS; MCF-7, Jyg-MCB, HeLa, A549, Panc-1, MiaPaCa2, KK47, A2058, G-361, RD, ASF 4-1, TIG-3-20, SVts-8, and H9c2 cells in EMEM (Wako) containing 10% FBS; BJMC3879, DLD-1, HT-29, Colo 201, HCT116, SW480, SW620, SW837, BxPC3, MKN1, MKN7, MKN45, T24, 253JBV, DU145, LNCaP, PC3, U-251MG, Rh30, Rh41, K562, Ba/F3 T315I, RPMI8226, KCL-22, and NOMO-1 cells in RPMI1640 (Wako) supplemented with 10% FBS; and human mammary epithelial cells (HMECs) in mammary epithelial cell basal medium (CC-3151, Lonza) supplemented with mammary epithelial cell growth medium BulletKit (CC-3150, Lonza). MCF-7, Jyg-MCB, DLD-1, WiDr, Colo 201, SW480, SW837, Panc-1, MiaPaCa2, MKN1, MKN7, MKN45, T24, PC3, U-251MG, NB-1, A2058, G-361, RD, K562, RPMI8226, KCL-22, NOMO-1, ASF 4-1, TIG-3-20, and SVts-8 cells were obtained from the Japanese Cancer Research Resources Bank; HCT116, DU145, LNCaP, and B16F10 cells from the Riken Bioresource Research Center; and KK47 from the Cell Resource Center for Biomedical Research of Tohoku University. MDA-MB-231, HeLa, A549, HT-29, SW620, and H9c2 cells were procured from the American Type Culture Collection. BxPC3 cells were obtained from the European Collection of Authenticated Cell Cultures. BJMC3879 and 253JBV cells were provided by the Osaka Medical College; Rh30 and Rh41 cells, by the Kyoto Prefectural University of Medicine; CMeC1 cells, by the University of Tokyo; and Ba/F3 T315I, by the National Cancer Center Japan. HMECs were purchased from Lonza. Cell lines used were confirmed as being negative by mycoplasma testing (MycoAlert, Lonza). All cells were cultured in a humidified incubator with 5% CO_2_ at 37°C. Amino acid or glucose was supplemented depending on the experiment (MEM nonessential amino acids solution 100×, 139-15651, Wako; MEM essential amino acids solution 50×, 132-15641, Wako; L-aspartic acid, A9256, Sigma-Aldrich; glucose, G8644, Sigma-Aldrich). The cells were treated with compounds dissolved in DMSO (PT) or water (metformin and phenformin). The DMSO concentration used in cell culture was 0.1% at the final concentration.

### Immunoblotting.

Cell and tissue lysates were prepared with 1% SDS buffer. The protein concentrations were measured by using a DC Protein Assay Kit (Bio-Rad). Protein samples (1–5 μg/lane) were resolved in 10%–12.5% SDS-PAGE gells and transferred to 0.45 μm PVDF membranes (Immobilon-P Membrane, EMD Millipore). After having been blocked with PVDF Blocking Reagent for Can Get Signal (TOYOBO) for 1 hour, the membranes were incubated overnight at 4°C with primary antibodies. The primary antibodies used for immunoblotting are summarized in Supplemental Table 2. The membranes were incubated at room temperature with HRP-linked anti–mouse IgG or anti–rabbit IgG as secondary antibodies (Cell Signaling Technology) diluted 1:4000. After 3 washes with TBS-T, the immunoblots were visualized by using the Luminata Forte Western HRP substrate (EMD Millipore). The loaded amount was verified with an anti–β-actin mouse monoclonal antibody (clone AC-74, Sigma-Aldrich, A5316). Please see Supplemental Methods for detailed information.

### RT-qPCR.

Total RNA from cells was purified by using NucleoSpin miRNA (MACHEREY-NAGEL). The concentration of total RNA was assessed by use of UV spectrophotometry. RNA integrity was checked with an Agilent 2100 Bioanalyzer (Agilent). cDNA was reverse transcribed from 0.5 μg of the total RNA by using a PrimeScript RT Reagent Kit (Takara). The cDNA was amplified with Universal SYBR Select Master Mix (Applied Biosystems), and signals were recorded with a Takara Thermal Cycler Dice Real Time System II. Relative expression levels were quantified by the ΔCt method and normalized to *B2m*. The gene used as an internal control was selected from *Tbp*, *B2m*, *Gapdh*, *Actb*, *Hprt* by using the NormFinder algorithm (https://moma.dk/normfinder-software).

### Histology and immunohistochemistry.

Tissues for H&E staining and immunohistochemistry were fixed in 4% neutral buffered paraformaldehyde, embedded in paraffin, sectioned at 5 μm, and mounted on microscope glass slides (S2226 for H&E, SCRE-05, for immunohistochemistry, Matsunami Glass). The slides were deparaffinized in lemosol and rehydrated by passage through graded alcohols. H&E staining was performed with Tissue-Tek Hematoxylin 3G (9131-4P, Sakura Finetek Japan) and Tissue-Tek Eosin (8659, Sakura Finetek Japan). For immunohistochemistry, the sections were incubated in 0.3% hydrogen peroxide in methanol for 20 minutes to inhibit endogenous peroxidase activity. For antigen retrieval, the sections were then autoclaved for 1 minute at 120°C in antigen retrieval buffers (Immunoactive, IA6500, IA9500, Matsunami Glass) and cooled down to room temperature. After 3 washes with TBS-T, the sections were blocked with 2.5% normal horse serum (S-2012-50, Vector Laboratories) and incubated with primary antibodies overnight at 4°C. The primary antibodies used are listed in Supplemental Table 2. The sections were then washed 3 times with TBS-T and incubated for 20 minutes with the secondary antibody conjugated with HRP (ImmPRESS HRP Horse Anti-Rabbit IgG Polymer Detection Kit, MP-7401, Vector Laboratories). After 3 washes with TBS-T, the signals were visualized with DAB chromogen solution (ImmPACT DAB Substrate, SK-4105, Vector Laboratories). The slides were counterstained by using hematoxylin or Giemsa stain. Histopathological images were acquired by using a microscope (BZ-X700, KEYENCE).

### Immunocytochemistry and confocal microscopy.

B16F10 cells were seeded onto coverslips at the concentration of 0.25 × 10^5^ cells/mL. After treatment with PT (0.3 μM) or DMSO and incubation for 72 hours, the cells were washed with ice-cold PBS and fixed with 4% paraformaldehyde in PBS for 30 minutes at 4°C. After 3 washes with PBS, the cells were made permeable with 0.1% Triton X-100 in PBS for 5 minutes and then blocked with 10% goat serum in PBS for 1 hour. Subsequently, the coverslips were incubated overnight at 4°C with anti–p-FAK^Y397^ (clone D20B1, Cell Signaling Technology, 8556) as the primary antibody. After 4 washes with PBS, the coverslips were incubated for 45 minutes at room temperature with anti-rabbit antibody conjugated with Alexa Fluor 488 (Life Technologies) as the secondary antibody and phalloidin conjugated with Alexa Fluor 568 (Life Technologies). The coverslips were finally incubated with DAPI (200 nM) for 5 minutes and mounted on slides with Lab Vision PermaFluor (Thermo Fisher Scientific). Confocal images were obtained with a laser scanning confocal microscope (LSM-710, Carl Zeiss). The images obtained were analyzed by using ImageJ (version 2.0.0-rc-69/1.52p, NIH).

### Statistics.

GraphPad Prism 8 (version 8.4.0), and JMP (version 12.2, SAS Institute) were used for statistical analysis and illustrating heatmaps. The detailed information on error bars, *P* values, and statistical tests is given in the figure legends. *P* values of less than 0.05 were considered significant. If not specified, biological replicates (sample size, *n =* 3) were employed for performing the experiments, and the measurements were taken from distinct samples in each experiment. All experiments were performed at least twice, and the technical and biological replicates were reliably reproduced.

### Data availability.

cDNA microarray data have been deposited in the Gene Expression Omnibus (GEO) under accession code GSE150348.

### Study approval.

All mouse experiments were approved by the IACUC of Gifu University and performed according to the NIH *Guide for the Care and Use of Laboratory Animals* (National Academies Press, 2011).

## Author contributions

KH and YA conceived the study. Development and screening of a phytochemical library, HPLC, and purification of PT were performed by RI. Viable cell counts for various cancer cell lines were carried out by KH, NS, YK, and TN. Spheroid formation assay; cell-cycle analysis; immunoblotting; Matrigel invasion assay; senescence-associated β-galactosidase staining; MitoTracker orange/green staining; mitochondrial ETC activity assays; determination of NAD/NADH ratios; cDNA microarray analysis; generation of Rho-0 cells; measurement for glucose, lactate, and pH in the culture medium; measurement of ATP/ADP ratio; cDNA microarray analysis; proteome analysis; scratch wound healing assay; cell attachment assay; and statistical analysis were performed by KH. RT-qPCR and animal experiments were done by KH and NS. Metabolome analysis was carried out by KH and TS. *NDI1* overexpression was performed by KH and RH. Histology and immunohistochemistry were performed by KH and HS. Immunocytochemistry, confocal microscopy, and Rac1 pulldown assay were performed by MN and HU. Transmission electron microscopy analysis was done by YI and KH. KH conducted data analysis. KH wrote the manuscript with contributions from YA. All authors read and approved the final manuscript.

## Supplementary Material

Supplemental data

## Version 1 09/01/2021

Electronic publication
