# Supplementary material for: Petasin potently inhibits mitochondrial complex I–based metabolism that supports tumor growth and metastasis
Source: J Clin Invest. 2021 Sep 1;131(17):e139933. doi: 10.1172/JCI139933 (PMC8409585; doi:10.1172/JCI139933)
Supplement: Supplemental data [file jci-131-139933-s197.pdf]

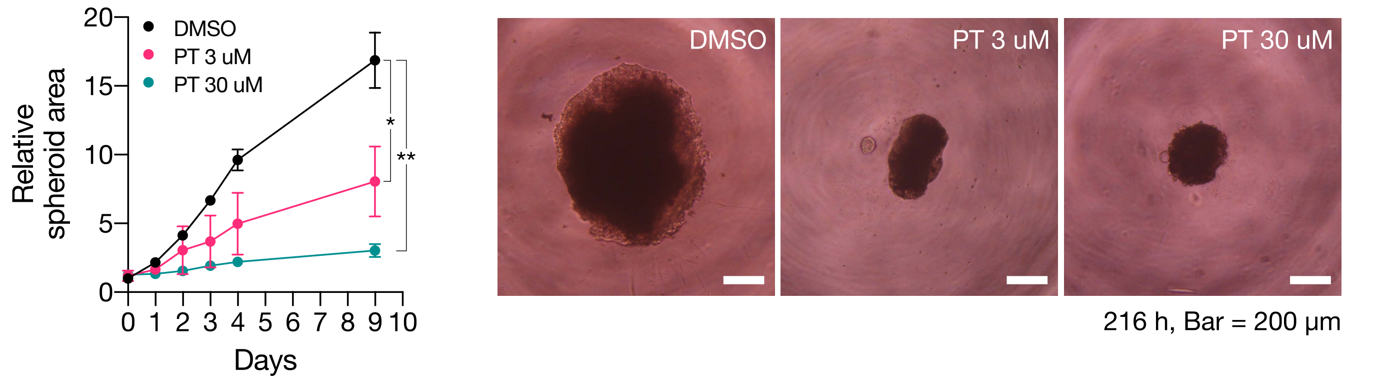

Supplemental Figure 1

Growth curve and representative images of the spheroid assay using B16F10 cells treated with PT (3 μM) or DMSO. Scale bar, 200 μm. Data are presented as the mean ± SE. *P<0.05; **P<0.01, Dunnett test.

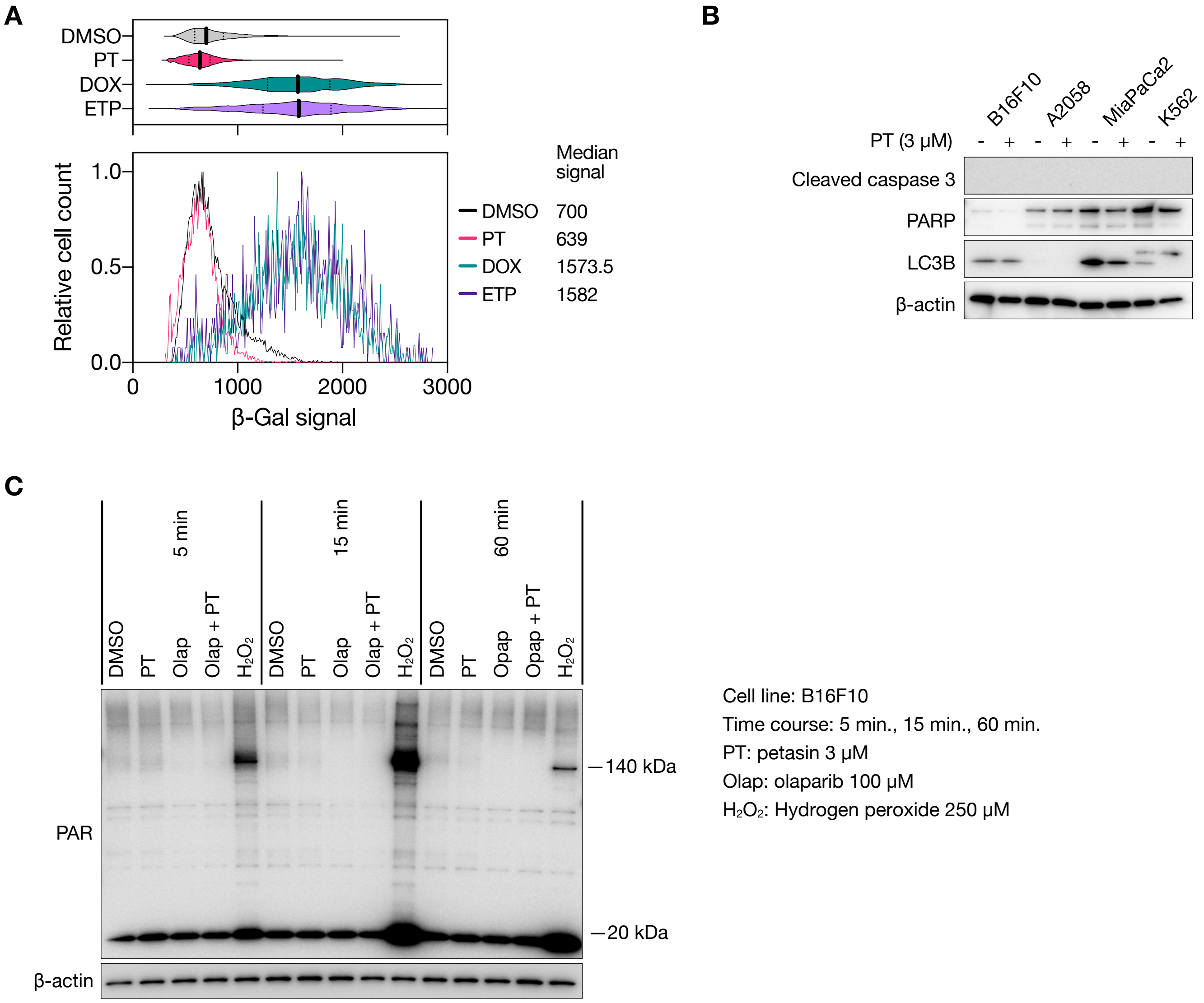

Supplemental Figure 2

(A) Histogram and violin plots for senescence-associated-β-galactosidase staining results for B16F10 cells treated for 48 hours with petasin (PT, 3 μM), doxorubicin (DOX, 0.2 μM) or etoposide (ETP, 10 μM) as positive controls or with DMSO as a negative control. (B) Immunoblots for cleaved caspase 3, PARP, LC3B, and β-actin (loading control) in melanoma (B16F10, A2058), pancreatic cancer (MiaPaCa2), and chronic myeloid leukemia (K562) cells treated for 72 hours with PT (3 μM) or DMSO. (C) Immunoblots for Poly/Mono-ADP Ribose (PAR) and β-actin (loading control) in B16F10 cells treated for 5, 15 or 60 min with PT (3 μM), Olaparib (negative control, a poly ADP ribose polymerase inhibitor, 100 μM) or hydrogen peroxide (positive control, H_2O_2, 250 μM).

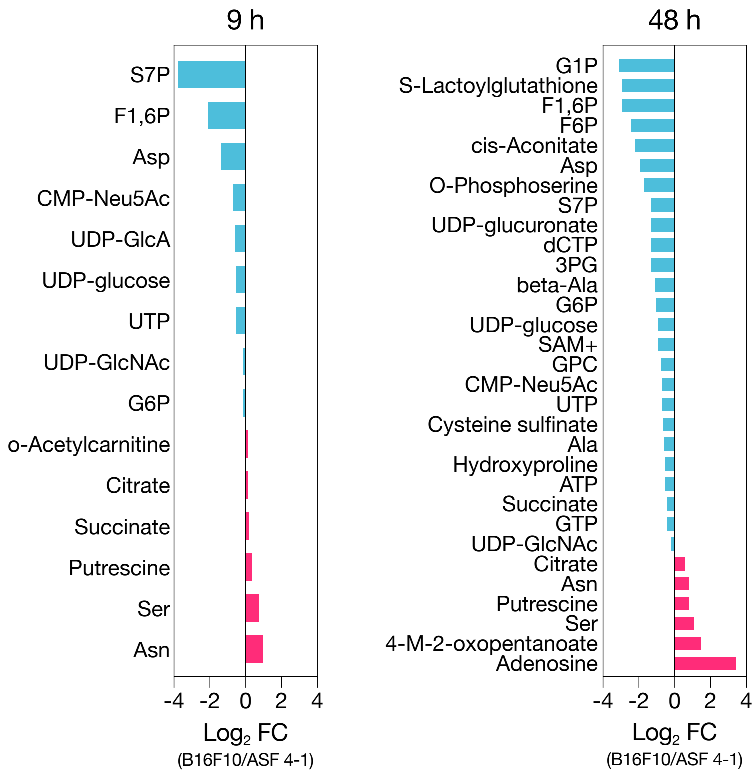

Supplemental Figure 3

List of significantly different metabolites between tumor (B16F10) and non-tumor (ASF 4-1) cells treated for 9 or 48 hours with petasin (3 μM) or DMSO. Bar graph for altered metabolites is marked with colors (red, high in tumor cells; blue, low in tumor cells). Metabolites with absolute $\log_2$ fold-changes (FC) greater than 0.585 and P-values of less than 0.05 were included.

Petasin (PT)

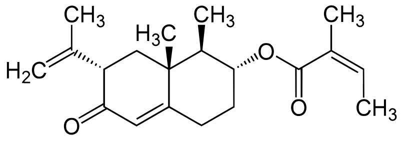

Metformin

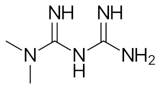

Phenformin

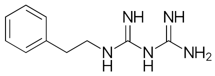

Rotenone

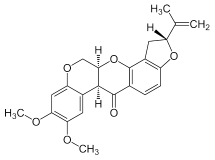

BAY 87-2243

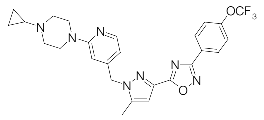

IACS-010759

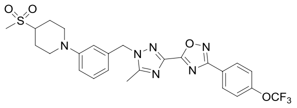

Supplemental Figure 4

Chemical structures of petasin (PT), metformin, phenformin, rotenone, BAY87-2243, and IACS-010759.

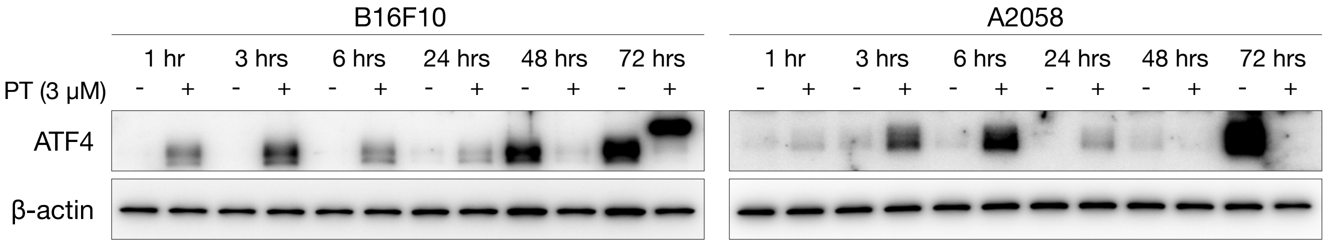

Supplemental Figure 5

Immunoblots for ATF4 and β-actin (loading control) in B16F10 and A2058 cells treated with PT (3 μM) at different time points (1, 3, 6, 24, 48, 72 hours after PT-treatment had started, original blot data of Figure 6C).

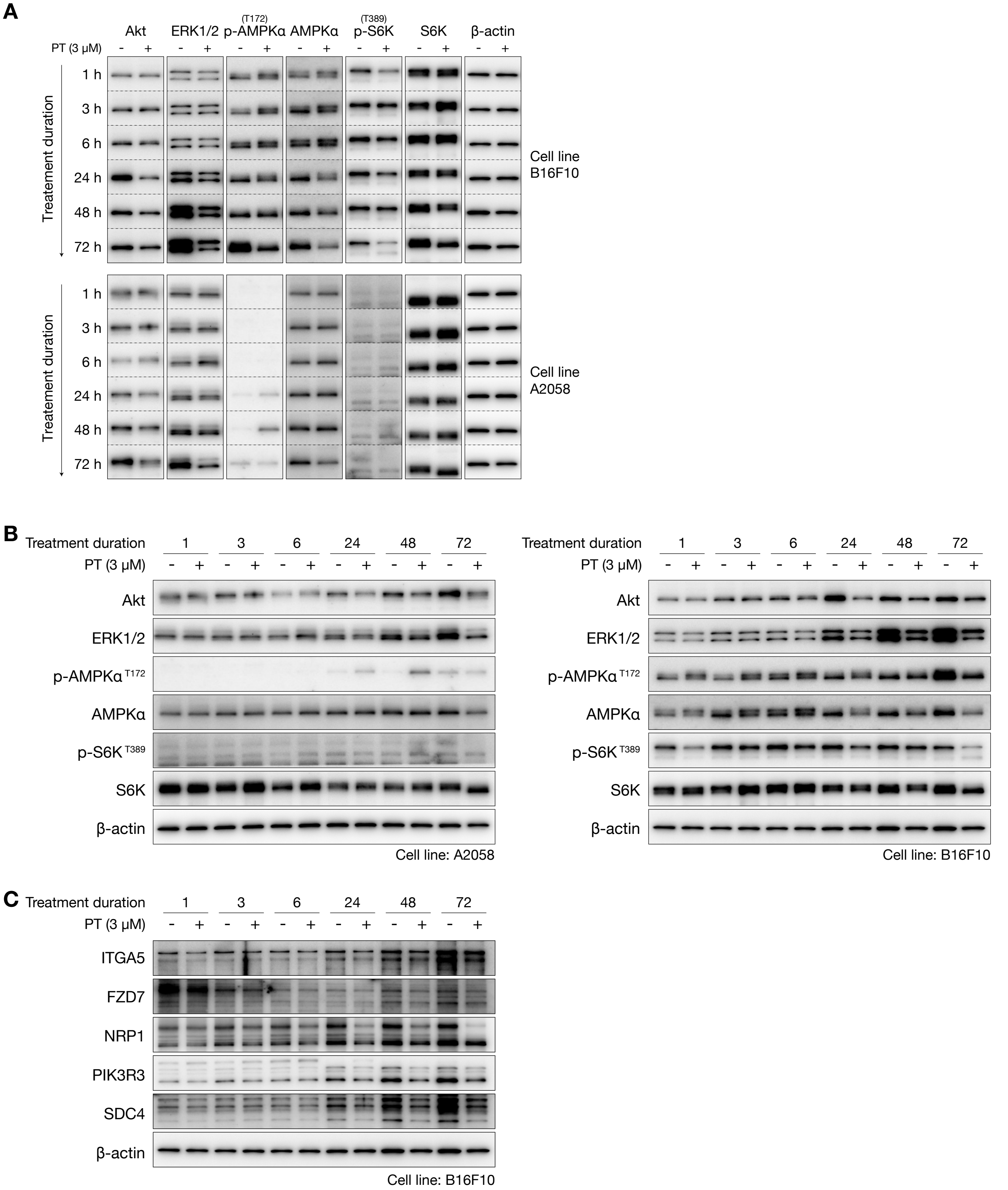

Supplemental Figure 6

(A) Immunoblots for Akt, ERK1/2, p-AMPKα^{T172}, AMPKα, p-S6K^{T389}, and S6K in B16F10 and A2058 cells treated with PT (3 μM) at different time points (1, 3, 6, 24, 48, 72 hours after PT treatment had started). The data were obtained from the same membrane for each target for comparison between different durations of treatment. (B) original blot data of A. (C) original blot data of Figure 7E.

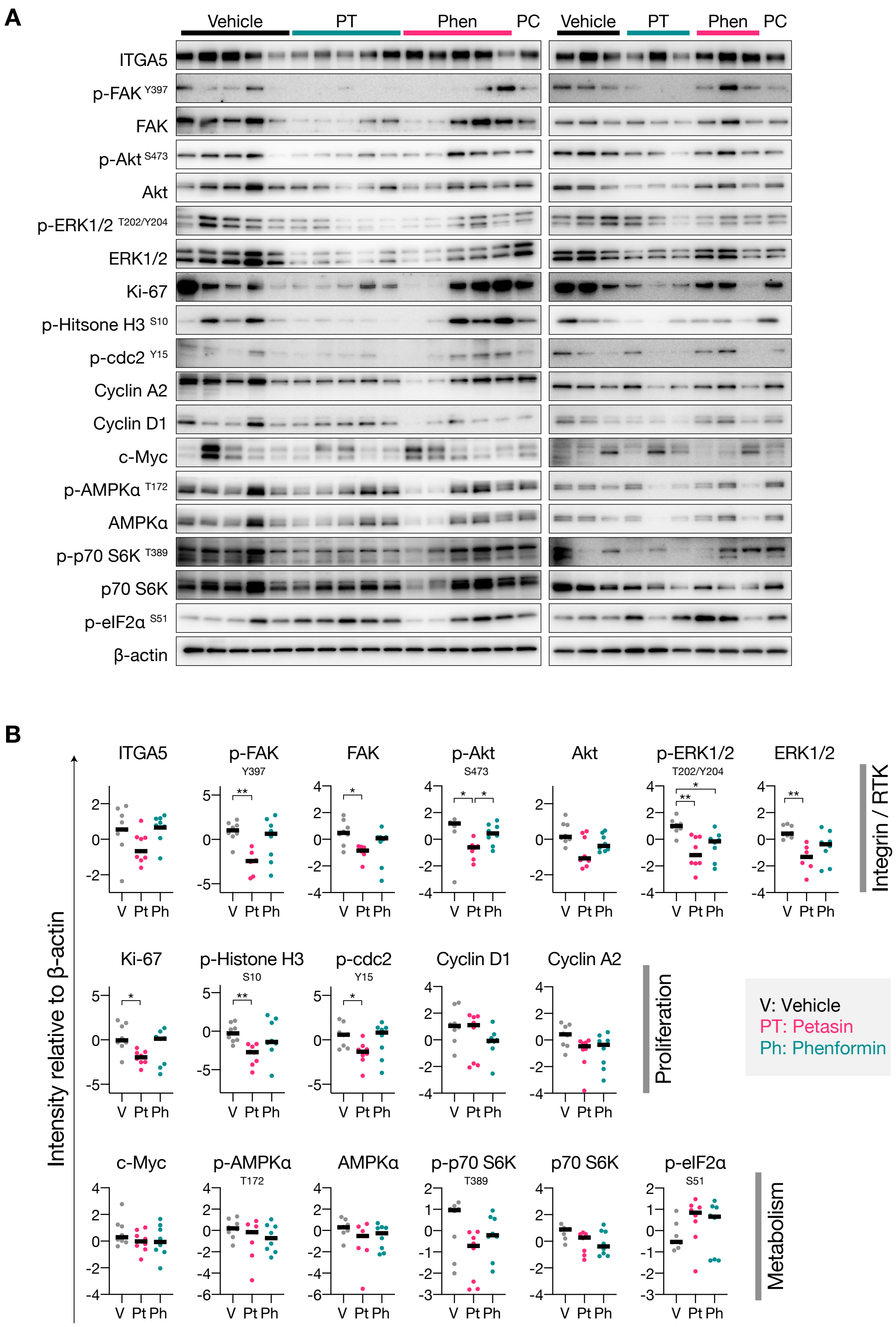

Supplemental Figure 7

(A) Immunoblots for oncogenes, cell cycle-related proteins, and metabolism-related proteins in the tumor samples from the B16F10 orthotopic syngeneic mouse model treated with petasin (PT, 50 mg/kg), phenformin (Phen, 50 mg/kg) or vehicle (Full data of Figure 8B). A pooled control (PC) was used for normalizing signals in different membranes. Vehicle: PBS containing 1% v/v DMSO and 10% v/v high-purity oleic acid. (B) Protein levels of tumor samples from mice treated with each agent (V, vehicle; Pt, petasin; Ph, phenformin). The protein levels were measured by densitometry and normalized with the levels of β-actin. Thick horizontal bars indicate the median. RTK, receptor tyrosine kinase. *P<0.05, **P<0.01, Kruskal-Wallis test (n=8).

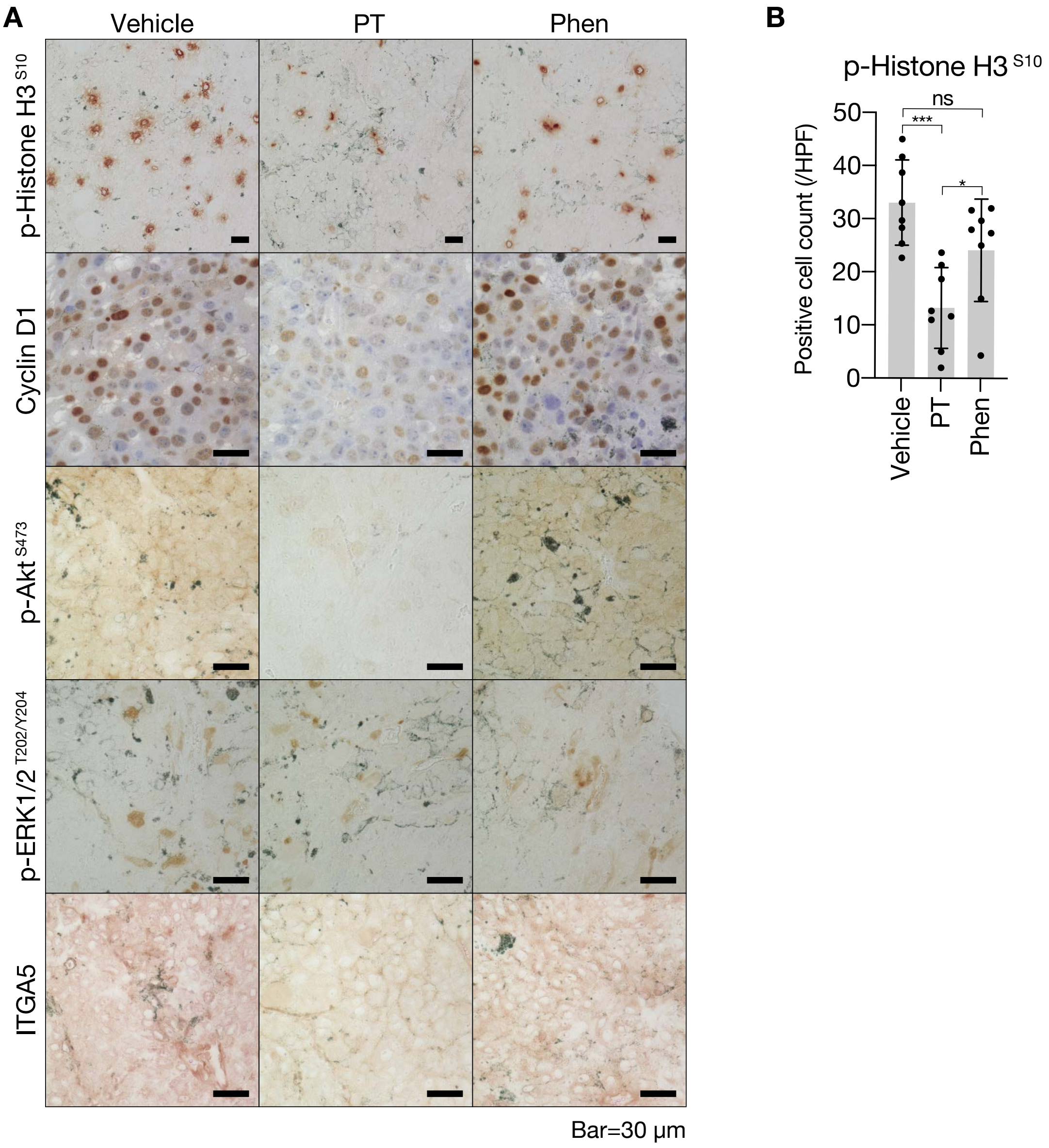

Supplemental Figure 8

(A) Representative images of immunohistochemical analysis for p-Histone H3 ^{S10}, Cyclin D1, p-Akt ^{S473}, p-ERK1/2 $^{T202/Y204}$, and ITGA5 in the tumor samples from the B16F10 orthotopic syngeneic mouse model treated with petasin (PT, 50 mg/kg), phenformin (Phen, 50 mg/kg) or vehicle (PBS containing 1% v/v DMSO and 10% v/v high-purity oleic acid.). Giemsa staining was used for the counterstain to distinguish endogenous melanin (dark green with Giemsa stain) and DAB stain (brown), except for Cyclin D1, which was stained with hematoxylin. Scale bar, 30 µm. (B) Count for p-Histone H3 ^{S10}-positive proliferating cells in the tumor tissues. Data are presented as the mean ± SD (n=8 per condition). HPF, high-power field. *$P<0.05$, ***$P<0.001$, ns (not significant), Tukey test.

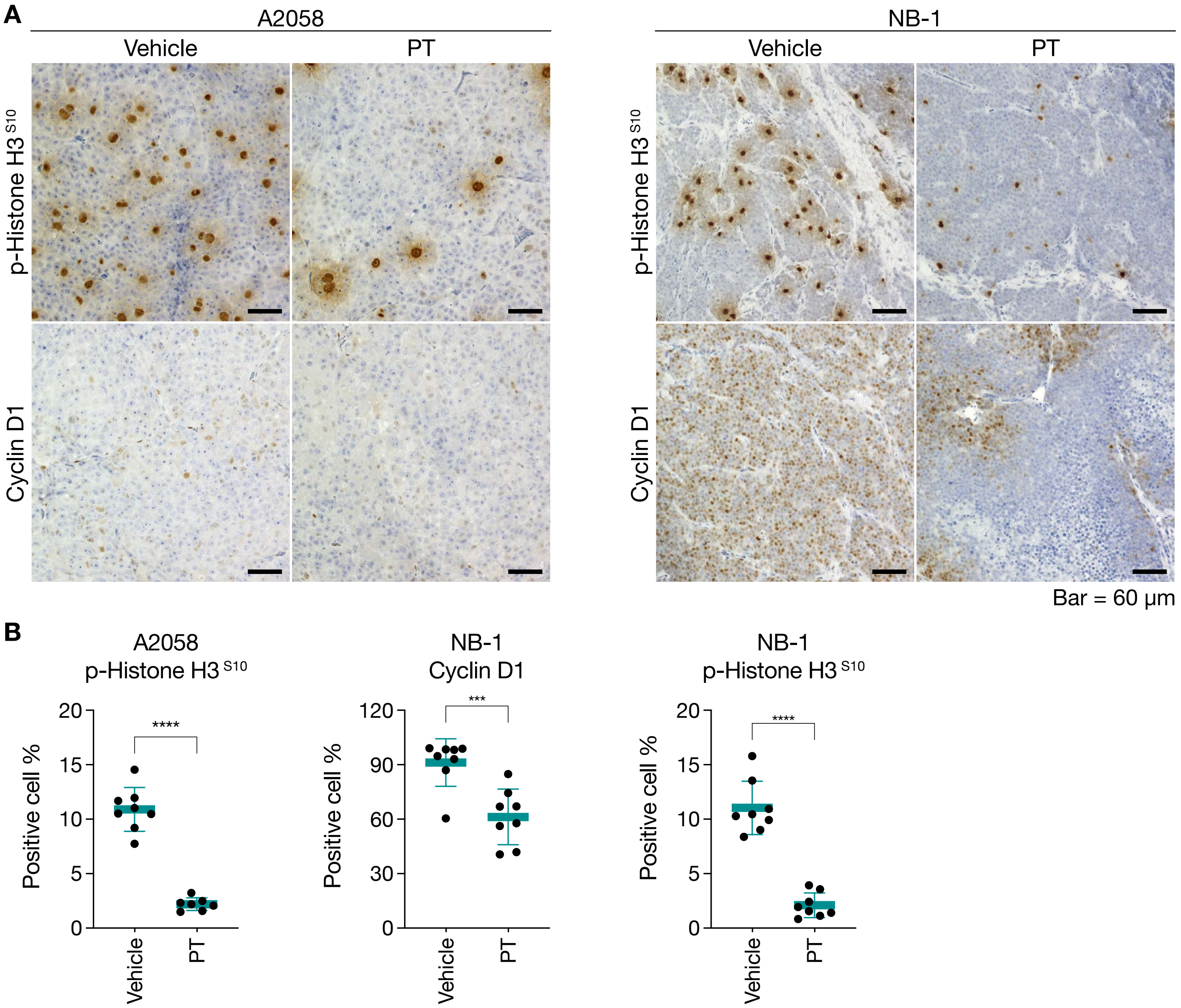

Supplemental Figure 9

(A) Representative images of immunohistochemical analysis for p-Histone H3 S10 and Cyclin D1 in the tumor samples from the A2058 and NB-1 model treated with petasin (PT, 50 mg/kg) or vehicle (PBS containing 1% v/v DMSO and 10% v/v high-purity oleic acid.). Scale bar, 60 μm. (B) Count for p-Histone H3 S10- or Cyclin D1-positive proliferating cells in the tumor tissues. Data are presented as the mean ± SD (n=8 per condition). ***P<0.001, ****P<0.0001, ns (not significant), 2-tailed unpaired Student's t-test.

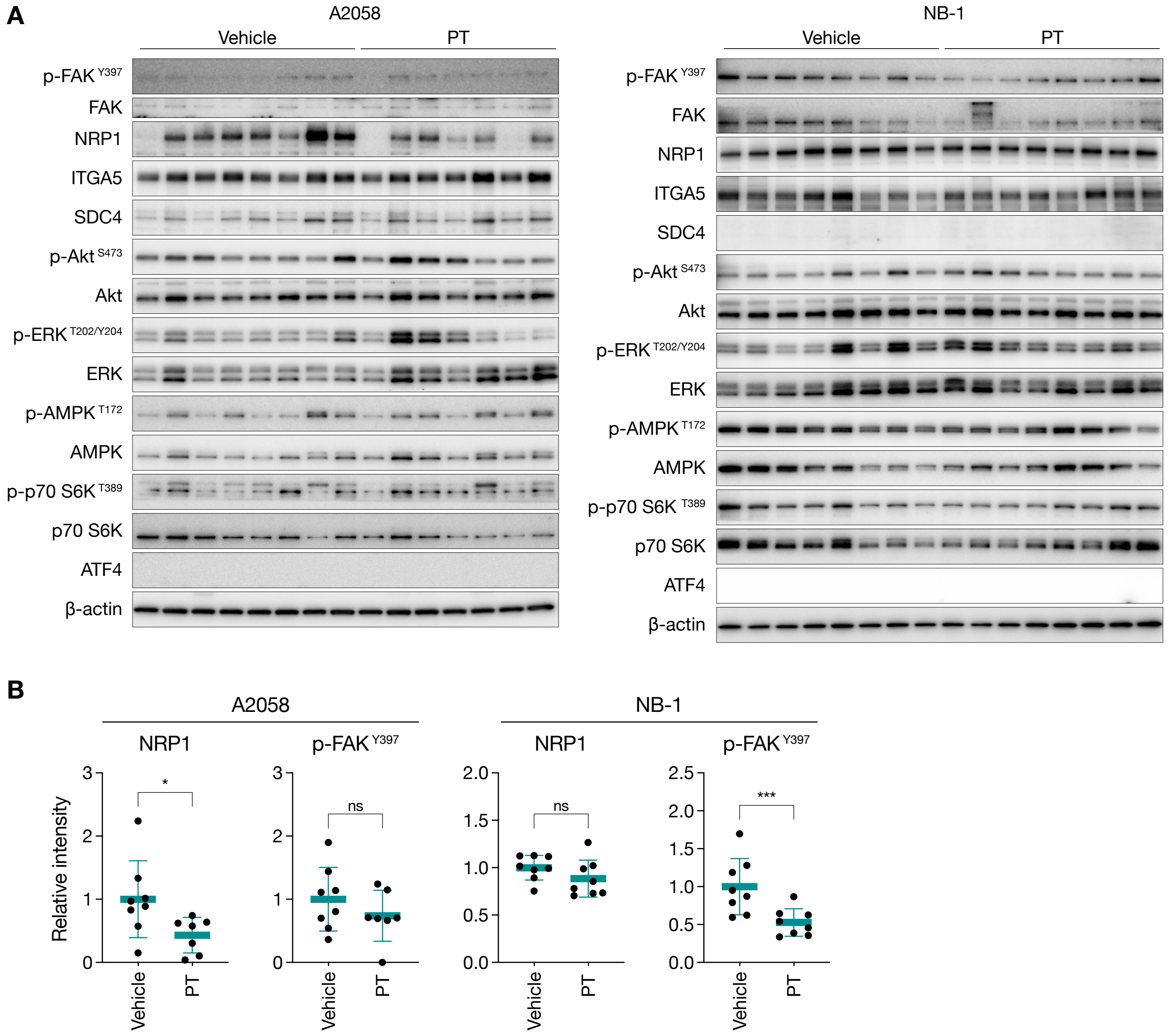

Supplemental Figure 10

(A) Immunoblots for oncogenes, cell-cycle-related proteins, and metabolism-related proteins in the tumor samples from the A2058 and NB-1 models treated with petasin (PT, 50 mg/kg) or vehicle (PBS containing 1% v/v DMSO and 10% v/v high-purity oleic acid). (B) Protein levels of tumor samples from mice treated with PT or vehicle. The protein levels were measured by densitometry and normalized with the levels of β-actin. Data are presented as the mean ± SD (n=8 per condition). A mouse in the A2058 model treated with PT was excluded from the analysis due to drug administration error. *P<0.05, ***P<0.001, ns (not significant), 2-tailed unpaired Student's t-test.

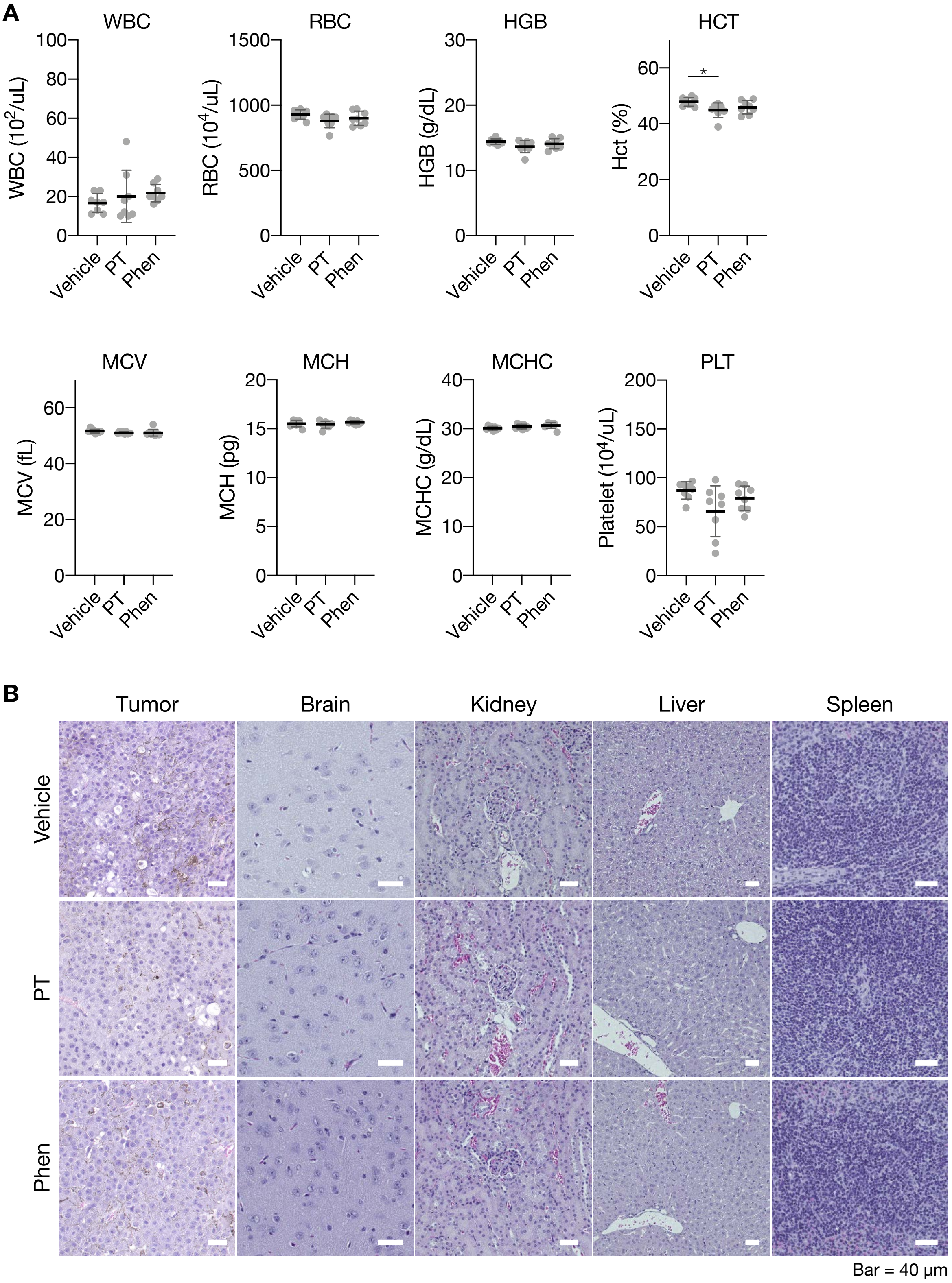

Supplemental Figure 11

(A) Complete blood counts for the orthotopic B16F10 model treated with petasin (PT), phenformin (Phen) or vehicle. There was no statistically significant difference between the treatment and control groups except in the case of hematocrit (HCT) values; however, the difference seen in the means of HCT was negligibly small. Data are presented as the mean ± SD (n=8 per condition). ns (not significant), Tukey test. WBC, white blood cell; RBC, red blood cell; HGB, hemoglobin; MCV, mean corpuscular volume; MCH, mean corpuscular hemoglobin; MCHC, mean corpuscular hemoglobin concentration; PLT, platelet. (B) Representative H&E images for tumor, brain, kidney, liver, and spleen in mice treated with PT, Phen or vehicle (n=8 per condition). Scale bar, 40 μm. Vehicle: PBS containing 1% v/v DMSO and 10% v/v high-purity oleic acid.

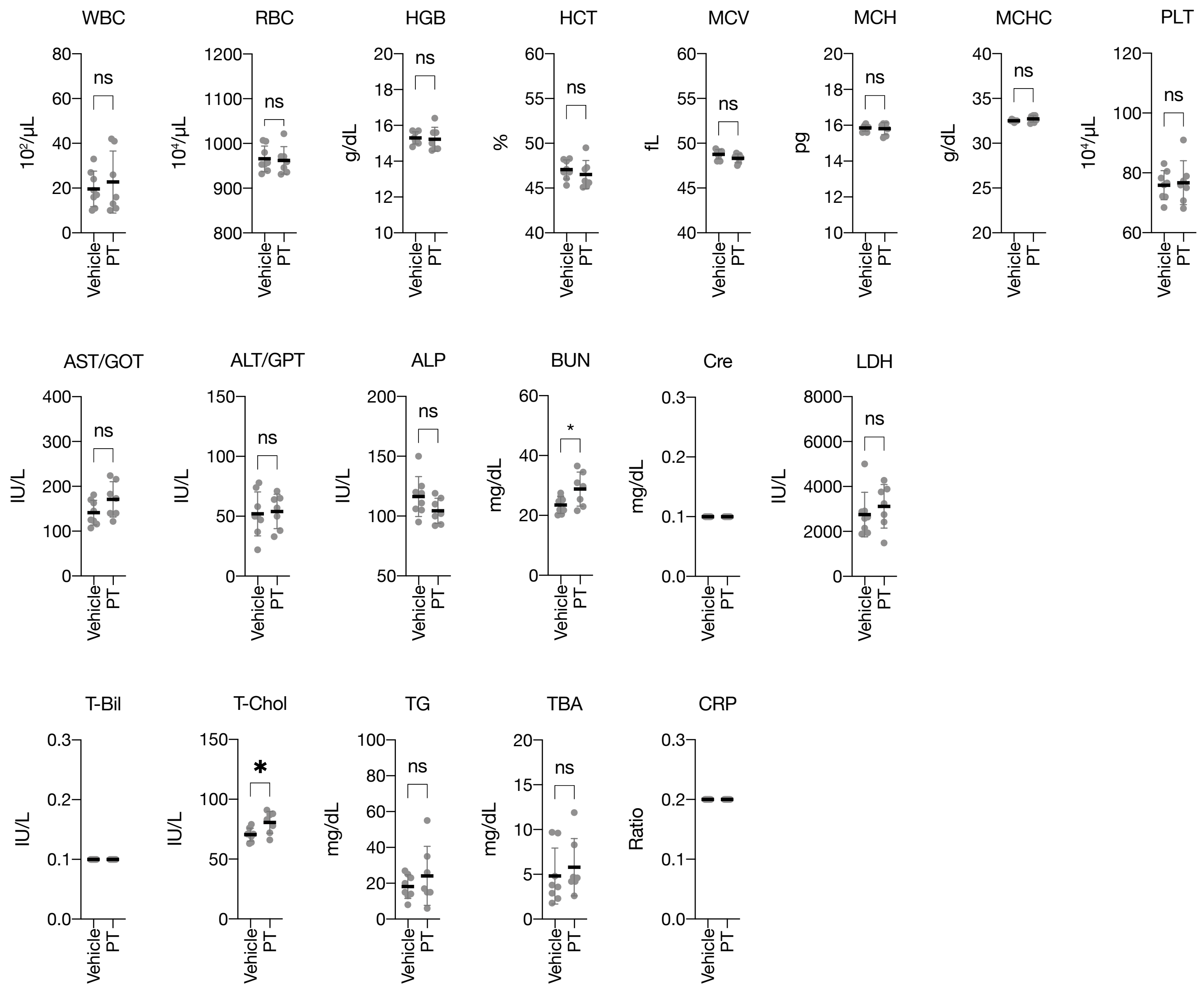

Supplemental Figure 12

Complete blood counts and blood biochemical profiles for A2058 xenograft model treated with petasin (PT 50 mg/kg) or vehicle. There was no statistically significant difference between the treatment and vehicle groups except in the case of the blood urea nitrogen (BUN), which difference in the means was negligibly small. Data are presented as the mean ± SD (n=8 per condition). ns (not significant), 2-tailed unpaired Student's t-test. WBC, white blood cell; RBC, red blood cell; HGB, hemoglobin; HCT, hematocrit; MCV, mean corpuscular volume; MCH, mean corpuscular hemoglobin; MCHC, mean corpuscular hemoglobin concentration; PLT, platelet; AST/GOT, aspartate aminotransferase/glutamic oxaloacetic transaminase; ALT/GPT, alanine aminotransferase/glutamic-pyruvic transaminase; ALP, alkaline phosphatase; Cre, creatinine; LDH, Lactate dehydrogenase; T-Bil, total bilirubin; T-Chol, total cholesterol; TG, triglycerides; TBA, Total bile acids; CRP, c-reactive protein.

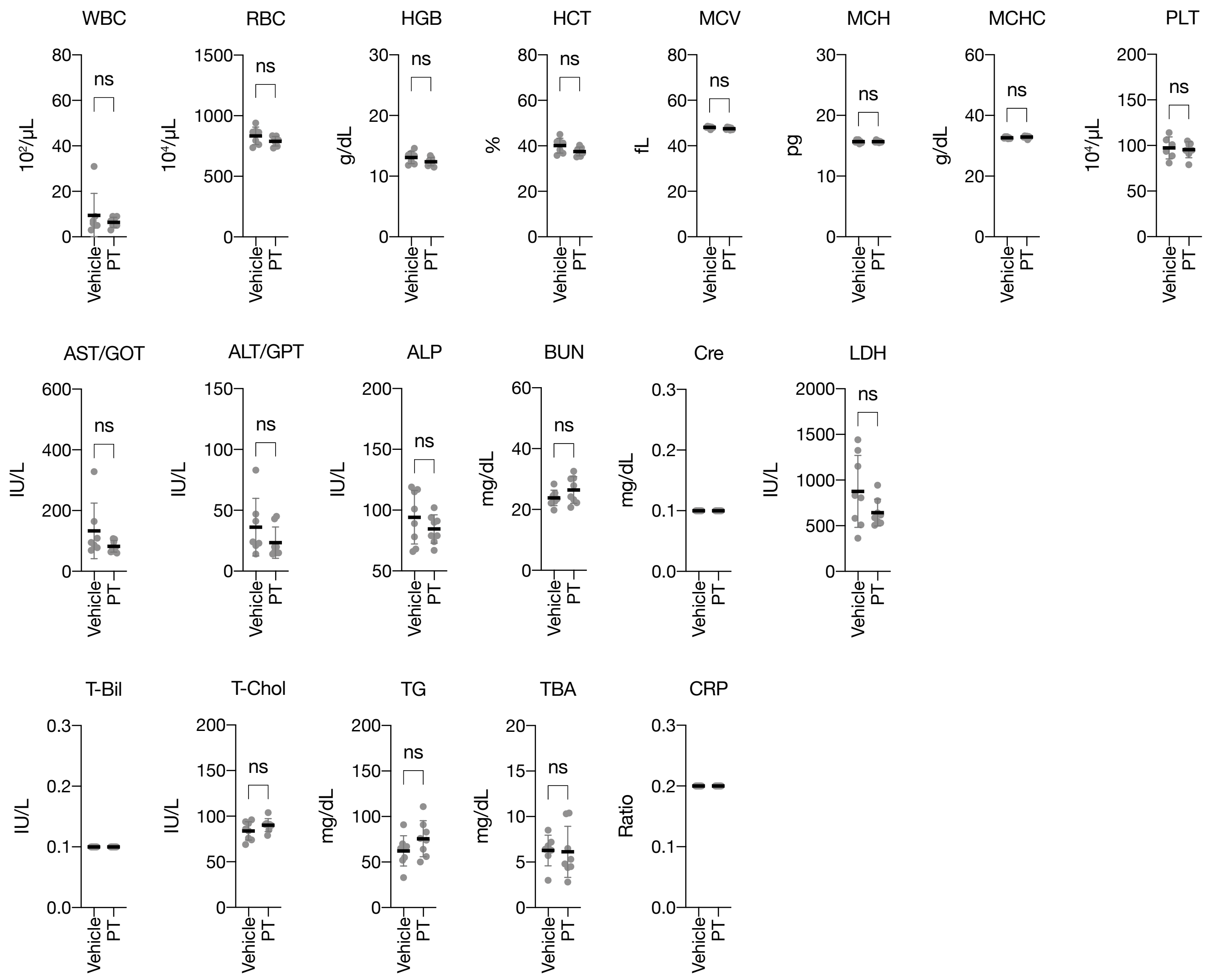

Supplemental Figure 13

Complete blood counts and blood biochemical profiles for NB-1 xenograft model treated with petasin (PT 50 mg/kg) or vehicle. There was no significant difference between the treatment and vehicle groups. Data are presented as the mean ± SD (n=8 per condition). ns (not significant), 2-tailed unpaired Student's t-test. WBC, white blood cell; RBC, red blood cell; HGB, hemoglobin; HCT, hematocrit; MCV, mean corpuscular volume; MCH, mean corpuscular hemoglobin; MCHC, mean corpuscular hemoglobin concentration; PLT, platelet; AST/GOT, aspartate aminotransferase/glutamic oxaloacetic transaminase; ALT/GPT, alanine aminotransferase/glutamic-pyruvic transaminase; ALP, alkaline phosphatase; BUN, blood urea nitrogen; Cre, creatinine; LDH, Lactate dehydrogenase; T-Bil, total bilirubin; T-Chol, total cholesterol; TG, triglycerides; TBA, Total bile acids; CRP, c-reactive protein.

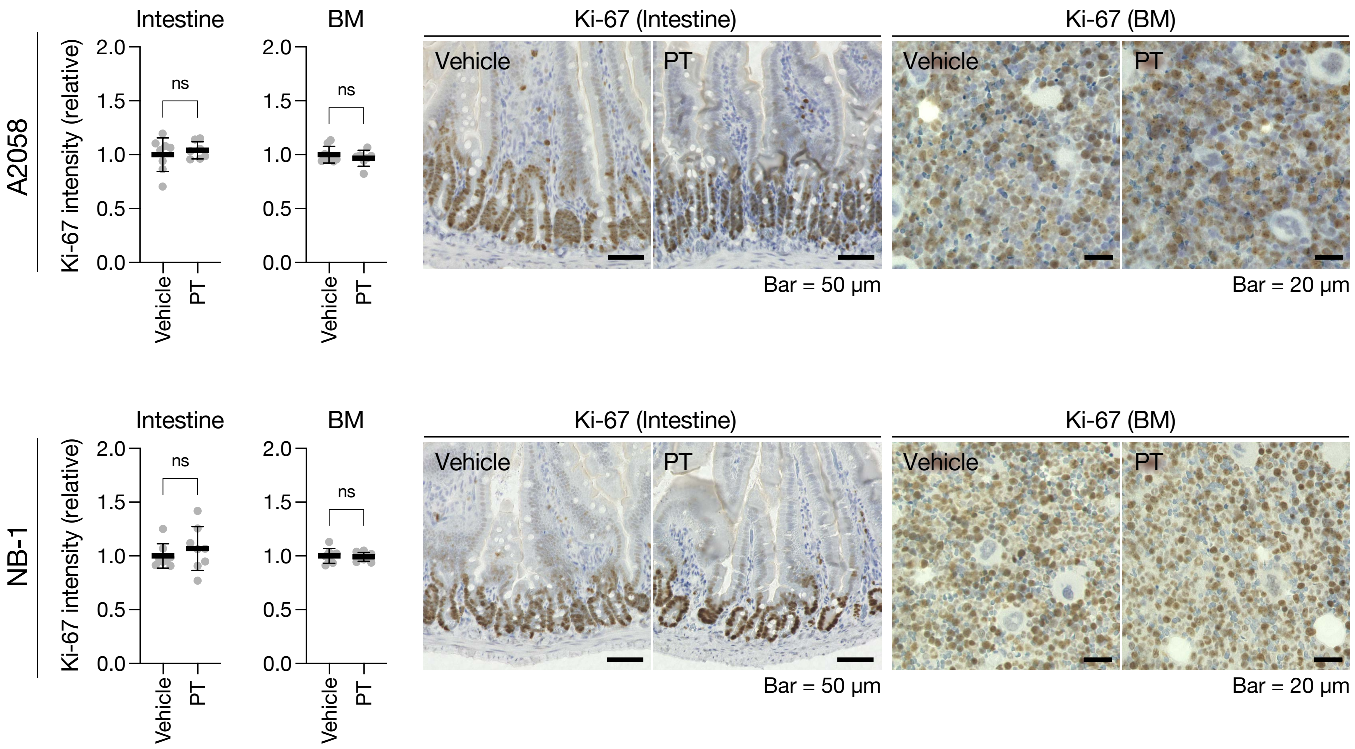

Supplemental Figure 14

Ki-67 index and representative immunohistochemical images of the intestine and bone marrow (BM) of the A2058 and NB-1 model mice treated with petasin (PT, 50 mg/kg) or vehicle (PBS containing 1% v/v DMSO and 10% v/v high-purity oleic acid). Scale bar: 50 μm for intestine; 20 μm for BM. Data are presented as the mean ± SD (n=8). ns (not significant), 2-tailed unpaired Student's t-test.

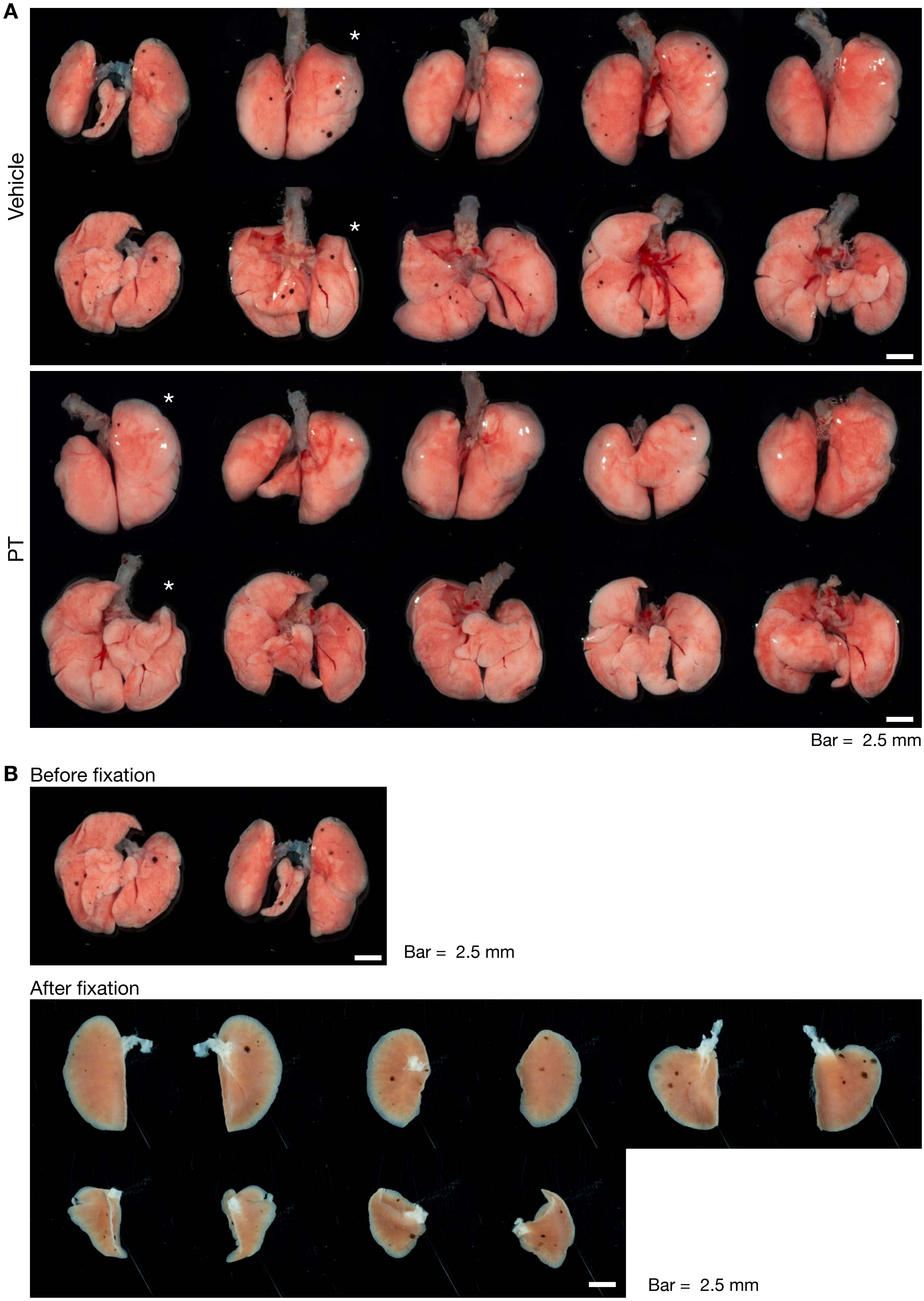

Supplemental Figure 15

(A) All gross images of lungs from lung colonization assay using B16F10 (n=5 per condition. Figure 10A,B). The mice intravenously injected B16F10 cells were treated with petasin (PT, 50 mg/kg) or vehicle (PBS containing 1% v/v DMSO and 10% v/v high-purity oleic acid). Lungs marked with asterisks were also presented in Figure 10B. Scale bar, 2.5 mm. (B) Detailed gross images of each lung lobe after fixation with 4% paraformaldehyde and images of the lungs before fixation. The images of the lungs before fixation were also presented in A. All visible spots on the lung surface were counted for the analysis. Scale bar, 2.5 mm.

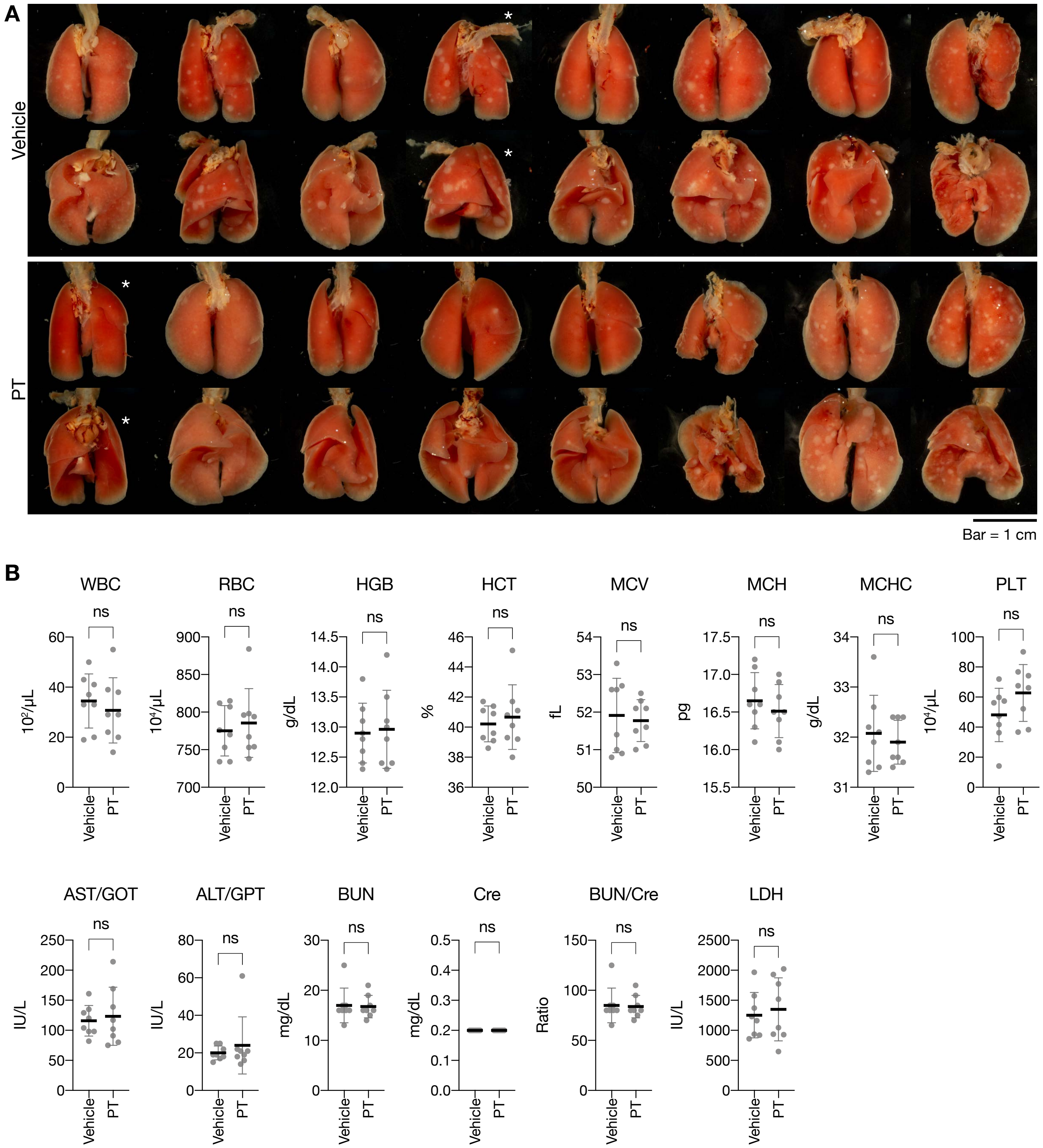

Supplemental Figure 16

(A) All gross images of lungs from the Jyg-MCB model treated with petasin (PT, 50 mg/kg) or vehicle (PBS containing 1% v/v DMSO and 10% v/v high-purity oleic acid, Figure 10C,D). Lungs marked with asterisks were also presented in Figure 10D. (B) Complete blood counts and blood biochemical profiles for the Jyg-MCB model treated with petasin (PT 50 mg/kg) or vehicle. There was no significant difference between the treatment and vehicle groups. Data are presented as the mean ± SD (n=8 per condition). ns (not significant), 2-tailed unpaired Student's t-test. WBC, white blood cell; RBC, red blood cell; HGB, hemoglobin; HCT, hematocrit; MCV, mean corpuscular volume; MCH, mean corpuscular hemoglobin; MCHC, mean corpuscular hemoglobin concentration; PLT, platelet; AST/GOT, aspartate aminotransferase/glutamic oxaloacetic transaminase; ALT/GPT, alanine aminotransferase/glutamic-pyruvic transaminase; BUN, blood urea nitrogen; Cre, creatinine; LDH, Lactate dehydrogenase. n=8 per condition. Scale bar, 1 cm.

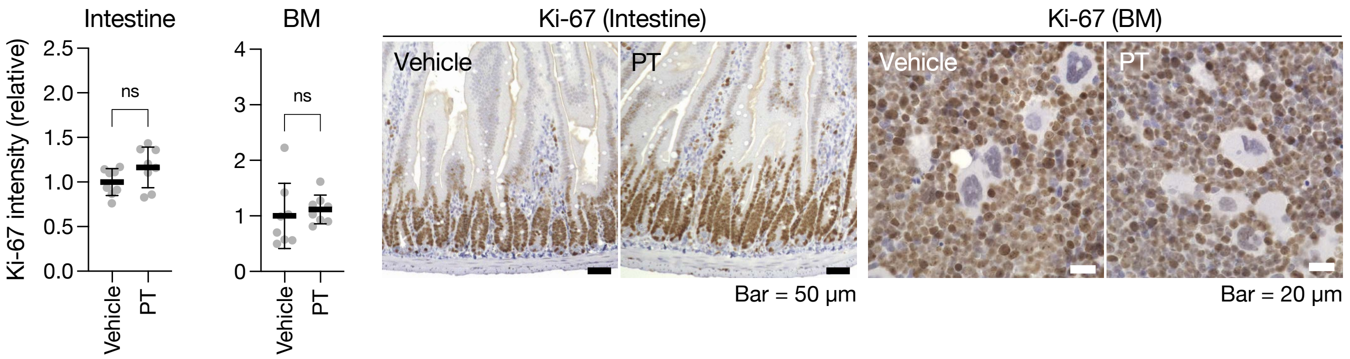

Supplemental Figure 17

Ki-67 index and representative immunohistochemical images of the intestine and bone marrow (BM) of the Jyg-MCB model treated with petasin (PT, 50 mg/kg) or vehicle (PBS containing 1% v/v DMSO and 10% v/v high-purity oleic acid). Scale bar: 50 μm for intestine; 20 μm for BM. Data are presented as the mean ± SD (n=8). ns (not significant), 2-tailed unpaired Student's t-test.

Library ID	Scientific or product name	Relative cell counts (%)
64	Petasites japonicus	48
219	Citrus × hassaku	54
89	Rosmarinus officinalis L.	54
121	Camellia sinensis	55
58	Chrysanthemum × morifolium Ramat.	63
183	Aristotelia Chilensis	64
91	Ocimum basilicum	66
139	Prunus mume	66
250	Phyllanthus emblica	71
299	Ocimum basilicum	71
355	Physalis alkekengi var. franchetii	71
293	Curcuma longa	73
373	Alpinia garanga	73
158	Lavandula angustifolia	73
294	Dioscorea bulbifera	74
56	Boswellia serrata	74
123	Phyllanthus emblica	75
218	Citrus hystrix	75
246	Brassica oleracea var. italica	75
339	Abelmoschus esculentus	75
367	Anthriscus cerefolium	76
371	Artemisia dracunculus	76
307	Cucumis sativus 'akageuri'	76
217	Citrus junos	76
6	Beta vulgaris var. cicla	77
140	Crataegus cuneata	77
188	Larix sibirica	77
255	Phlebodium aureum	78
25	Armoracia rusticana	78
138	Pseudocydonia sinensis	78
387	Pinus L.	78
79	Morus	79
216	Citrus unshiu	79
61	Cichorium intybus L.	79
162	Spinacia oleracea	79
222	Punica granatum	79
239	FloraGLO Lutein10%	79
197	Vigna angularis	80
276	Achillea millefolium	80
370	Diplotaxis tenuifolia	80
81	Sesamum indicum L.	80
125	Eurycoma longifolia	80
190	Phaseolus vulgaris	80
227	Olea europaea L.	80
228	Nemacystus decipiens	80
130	Musa spp.	81
292	Zingiber mioga	81
34	Brassica Rapa	81
49	Cucurbita pepo L. 'Melopepo'	81
50	Momordica charantia L.var.pavel Crantz	81
104	Peucedanum japonicum	81
202	Apios americana Medik.	81
256	Pisum sativum	81
421	Chenopodium quinoa	81
215	Citrus junos	81
115	Picrorhiza kurroa	81
352	Apium graveolens var. dulce	82
114	Fagopyrum esculentum	82
194	Pisum sativum L.	82
200	Pisum sativum L.	82
203	Millettia reticulata	82
257	Lutein P40A	82
378	Brassica oleracea var. botrytis	82
177	Castanea sativa	82
362	Momordica charantia var. pavel	83
258	Glycine max 'Kuromame'	83
392	Anthriscus cerefolium	83
117	Vaccinium myrtillus L.	83
204	Fortunella	83
304	Anredera cordifolia	83
174	Vitis spp	83

Library ID	Scientific or product name	Relative cell counts (%)
297	Cucurbita pepo	83
309	Capsicum annuum L. 'grossum'	84
21	Brassica oleracea var. italica	84
173	Vitis vinifera	84
186	Actinidia deliciosa	84
71	Andrographis paniculata	84
308	Daucus carota L. var.sativa DC.	84
281	Equisetum arvense	84
364	Capsicum annuum L.	84
301	Phaseolus vulgaris L.	85
305	Gynura bicolor	85
283	Ocimum tenuiflorum	85
35	Brassica junce	85
144	Hibiscus sabdariffa	85
334	Eruca vesicaria	85
172	Vitis spp	86
409	Pisum sativum L.	86
336	Cucurbita moschata	86
418	Fagopyrum esculentum	86
269	Origanum majorana L.	86
3	Salsola komarovii	86
20	sprout of the Brassia oleracea ver italica	86
33	Eutrema japonicum (Miq.) Koidz.	86
76	Engelhardtia chrysolepis	86
108	Daucus carota subsp. sativus	86
161	Pfaffia Paniculata	86
390	Brassica juncea	86
296	Brassica oleracea var. gongylodes	86
273	Morus alba	86
376	Brassica oleracea var. acephala	87
274	Fragaria vesca	87
412	Basella alba L.	87
7	Uncaria tomentosa	87
44	Oryza sativa	87
90	Ocimum tenuiflorum L.	87
234	Allium cepa	87
240	Lutein Extract	87
241	Lutein Soft Extract	87
272	Mentha spicata L.	87
122	Mallotus japonicus	87
295	Brassica oleracea var. gongylodes	87
245	Citrus × limon	87
263	Petasites japonicus	88
348	vulgaris vulgaris	88
31	Diplotaxis tenuifolia (L.) DC.	88
105	Coriandrum sativum L	88
167	Ipomoea batatas	88
181	Graptopetalum paraguayense	88
232	Oryza sativa	88
420	Cucurbita moschata	88
337	Aralia cordata	88
271	Mentha suaveolens	88
116	Phellinus igniarius	88
384	Lens culinaris	88
171	Vitis coignetiae	89
298	Solanum melongena L.	89
22	Brassica oleracea L. var. capitata	89
53	Cyclocybe aegerita	89
63	Helianthus tuberosus	89
201	Tamarindus indica L	89
383	Cicer arietinum L.	89
385	Lens culinaris	89
358	Cucurbita moschata	89
286	Echinacea	89
179	Rhodiola rosea	89
279	Origanum vulgare L.	90
360	Oenanthe javanica	90
92	Drynaria fortunei	90
322	Allium cepa "pekorosu"	90
2	Theobroma cacao	90
113	Pandanus Amaryllifolius	90

Library ID	Scientific or product name	Relative cell counts (%)
135	Mesembryanthemum crystallinum	90
157	Allium cepa	90
176	Myrcia Spaherocarpa	90
184	Ptychopetalum olacoides	90
191	Phaseolus vulgaris	90
198	Psophocarpus tetragonolobus	90
230	Dioscorea polystachya	90
226	Olea europaea	90
65	Echinacea purpurea	90
302	Corchorus olitorius L.	91
314	Zea mays L.	91
306	Luffa cylindrica（L.）Roem.	91
23	Brassica rapa L. var. nippo-oleifera	91
43	Oryza sativa	91
46	Acanthopanax sieboldianus	91
55	Diospyros kaki Thunb.	91
68	Artemisia dracunculus L.	91
97	Sambucus nigra	91
160	Celosia argentea	91
193	Pisum sativum L.	91
346	Flammulina velutipes (Curt.: Fr.) Sing.	91
52	Lagenaria siceraria var.clavata	91
178	Rhodiola rosea	91
277	Urtica thunbergiana	91
264	Petasites japonicus	91
414	Zanthoxylum bungeanum	91
342	Solanum melongena	91
406	Salsola komarovii	91
311	Capsicum annuum L. 'grossum'	91
335	Coriandrum sativum L.	92
338	Capsicum annuum L.	92
280	Mentha × piperita var. citrata cv. 'Orange'	92
386	Helianthus annuus	92
377	Cichorium intybus L.	92
251	Citrus ×paradisi	92
262	Petasites japonicus	92
5	Beta vulgaris ssp. vulgaris var. vulgaris	92
24	Brassica rapa L. var. hakabura.	92
30	Isatis tinctoria	92
51	Ipomoea alba	92
87	Perilla frutescens (L.) Britton var. crispa (Thunb.) H.Deane	92
136	Aronia	92
147	Malus pumila	92
164	Pleurotus cornucopiae var. citrinopileatus	92
254	-	92
260	Hordeum vulgare	92
357	Glebionis coronaria (L.) Cass. ex Spach	92
389	Brassica juncea	92
267	Pteridium aquilinum	92
175	Psidium guajava L.	92
321	Dioscorea japonica	92
253	Serenzo	93
259	Dark Brown Rice Vinegar	93
290	Brassica oleracea x	93
268	Zanthoxylum piperitum	93
388	Vigna radiata	93
359	Gynura bicolor	93
10	Morinda citrifolia	93
18	Brassica oleracea var. botrytis	93
19	Brassica napus L. ver. napobrassica	93
28	Raphanus sativus var. sativus	93
29	Raphanus sativus L. var. longipinnatus L.H.Bailey.	93
47	Aralia cordata	93
231	Daphniphyllum macropodum	93
243	Fucus vesiculosus	93
249	Black vinegar	93
75	Juglans	93
170	Vitis spp	93
349	Hyacinthus orientalis *Anastasia*	93
381	Ocimum basilicum L.'Dark Oparl'	94
275	Trifolium pratense L.	94

Library ID	Scientific or product name	Relative cell counts (%)
315	Solanum melongena	94
221	Punica granatum	94
38	Avena sativa	94
17	Brassica oleracea cultivar	94
48	Aralia elata	94
60	Cichorium endivia L	94
78	Ziziphus jujuba	94
83	Colocasia antiquorum var. toonoimo	94
106	Anthriscus cerefolium	94
199	Glycine max	94
340	Cucumis melo var. makuwa	94
236	Cladosiphon okamuranus	94
120	Camellia japonica L.	94
185	Actinidia deliciosa	95
343	Tricholoma matsutake (S. Ito et Imai) Sing	95
341	Cucurbita pepo L. 'Melopepo'	95
265	Aralia cordata	95
278	Stevia rebaudiana	95
350	Grifola frondosa	95
313	Brassica oleracea	95
16	Brassica napus	95
62	Arctium lappa	95
129	Tropaeolum majus	95
148	Coprinus comatus	95
159	Amaranthus	95
163	Pleurotus eryngii var. tuoliensis	95
224	Paullinia cupana	95
242	Lycopersicon esculentum	95
261	Hordeum vulgare	95
220	Pomegranate extract	95
407	Zea mays	95
214	Citrus sinensis	95
366	Anethum graveolens L.	95
331	Raphanus sativus var. sativus	95
419	Pinus densiflora	96
32	Lepidium meyenii	96
59	Cichorium intybus	96
82	Colocasia antiquorum var. toonoimo	96
102	Apium graveolens var. dulce	96
155	Allium schoenoprasum var. foliosum	96
156	Allium oschaninii	96
363	Eruca sativa mill	96
66	Tithonia diversifolia	96
143	Rosa roxburghii	96
375	Pimenta Racemosa	96
182	Lentinula edodes	96
12	Humulus lupulus	97
15	Brassica oleracea var. acephala	97
67	Artemisia indica var. maximowiczii	97
70	Flammulina velutipes (Curt.: Fr.) Sing.	97
100	Anethum graveolens L.	97
109	Glehnia littoralis	97
131	Nelumbo nucifera	97
213	Citrus sudachi	97
248	Camponotus japonicus	97
404	Ipomoea aquatica	97
287	Passiflora caerulea	97
289	Bupleurum scorzonerifolium	97
282	Salvia officinalis	97
212	Citrus unshiu	97
300	Brassica juncea	97
330	Zingiber officinale	97
266	Fallopia japonica	97
169	Vitis labrusca	98
103	Peucedanum japonicum Thunb.	98
345	Panicum miliaceum	98
329	Auricularia auricula-judae	98
319	Gynura bicolor	98
211	Citrus unshiu	98
98	Ribes nigrum	98
4	Beta vulgaris ssp. vulgaris var. vulgaris	98

Library ID	Scientific or product name	Relative cell counts (%)
27	Raphanus sativus var. longipinnatus	98
42	Oryza sativa subsp. japonica	98
69	Cichorium intybus	98
107	Daucus carota subsp. sativus	98
151	Agaricus subrufescens	98
153	Allium cepa	98
154	Allium tuberosum	98
165	P. eryngii var. tuoliensis CJ.Mou	98
192	Styphnolobium japonicum	98
247	Phyllanthus emblica	98
405	Allium cepa	98
11	Peucedanum japonicum	98
403	lilium lancifolium	98
391	Brassica juncea	98
411	Glycine max	98
73	Apocynum venetum	99
206	Zanthoxylum bungeanum	99
393	Anthriscus cerefolium	99
318	Cryptotaenia canadensis subsp. japonica	99
127	Salacia reticulata	99
110	Carum carvi	99
37	Ginkgo biloba	99
195	Cassia alata	99
402	Allium ampeloprasum var. sectivum	99
417	Citrus grandis	99
14	Brassica rapa L. var. rapa	99
77	Hovenia dulcis	99
189	Pinus densiflora	99
368	Mentha nemorosa	99
303	Pisum sativum	99
74	Malpighia emarginata DC.	99
291	Coriandrum sativum L.	99
396	Hosta sieboldiana	99
416	Cichorium intybus L.	99
324	Perilla frutescens	99
408	Vigna unguiculata	99
252	Lactobacillus Buchneri	100
395	Brassica oleracea var. italica	100
332	Raphanus sativus var. sativus	100
40	Zizania latifolia L.	100
86	Perilla frutescens (L.) Britton var. crispa (Thunb.) H.Deane	100
142	Rosaceae Rosa	100
180	Rhpdiola Rosea	100
229	Dioscorea japonica	100
235	Pfaffia Paniculata	100
237	-	100
9	Coffea arabica	100
132	Carica papaya	100
141	Rosaceae Rosa	100
244	Haematococcus lacustris	100
150	Agaricus subrufescens	100
233	Ruscus aculeatus	100
111	Pimpinella anisum	100
26	Raphanus sativus L. var. longipinnatus L.H.Bailey	101
284	Aloysia citrodora	101
320	Colocasia gigantea	101
399	Ziziphus jujuba	101
13	Brassica oleracea var. italica	101
41	Oryza sativa	101
54	Acer palmatum	101
101	Foeniculum vulgare	101
133	Cistanche tubulosa （schenk） wight	101
210	Citrus depressa	101
344	Canavalia gladiata	101
95	Zingiber officnale Rosc	101
149	Agaricus Blazei Murrill	101
119	Camellia japonica L.	101
96	Viburnum dilatatum	102
205	Zanthoxylum piperitum	102
354	Actinidia arguta	102
39	sasa veitchi	102

Library ID	Scientific or product name	Relative cell counts (%)
410	Glycine max	102
168	Geranium dielsianum.	102
397	Capsicum annuum L.	102
1	Adansonia	102
333	Beta vulgaris var. cicla (L.) K.Koch	102
209	Citrus junos	102
225	Litchi chinensis	102
285	Mentha x piperita L.	103
398	Pastinaca sativa	103
124	Houttuynia cordata	103
187	Larix kaempferi	103
270	Calendula officinalis	103
379	Allium ampeloprasum var. porrum	103
400	Lycium chinense	103
413	Allium schoenoprasum L. var. foliosum Regel	103
422	Amaranthus caudatus	103
310	Capsicum annuum L. 'grossum'	103
312	Capsicum annuum L. 'grossum'	103
327	Arctium lappa L.	103
365	Allium schoenoprasum L.	104
94	Zingiber officinale	104
84	Ajuga decumbens Thunb	104
85	Perilla frutescens	104
134	Cistanche salsa	104
196	Astragalus sinicus L.	104
238	Moringa oleifera	104
118	Vaccinium corymbosum	104
415	Armoracia rusticana	104
288	Malva	104
112	Azadirachta indica	105
351	Arachis hypogaea	105
99	Aloe vera (L.) Burm.f.	105
208	Citrus aurantium	105
72	Gymnema sylvestre	105
316	Capsicum annuum	106
361	Abelmoschus esculentus	106
128	Handroanthus impetiginosus	106
88	Origanum majorana	106
36	Linum usitatissimum	106
146	Malus pumila	106
369	Pimenta Racemosa	106
223	Lagerstroemia speciosa	107
317	Cucurbita pepo L. 'Melopepo'	107
57	Silybum marianum	107
80	Erythroxylum catuaba	107
166	Gynura bicolor	107
372	Cymbopogon citratus	107
401	Vigna unguiculata	107
326	Camellia sinensis	108
152	Allium sativum	109
8	Coffea arabica	109
137	Fragaria ananassa	109
145	Amygdalus persica L.	109
207	Citrus sinensis (L.) Osbeck	109
394	Sparassis crispa	110
356	Perilla frutescens	110
328	Oryza sativa	111
374	Pandanus Amaryllifolius	112
323	Perilla frutescens var. crispa	112
353	Asparagus officinalis	112
347	Beta vulgaris ssp. vulgaris	114
382	Phaseolus vulgaris	115
93	Kaempferia parviflora	116
325	Allium chinense	119
380	Rheum rhabarbatum L.	121
45	Acanthopanax senticosus (Rupr. et Maxim.) Harms	122
126	Salacia	125

Supplemental Table 2 List of antibodies used

Antigen	Antibody Clone	Clonality	Vendor	Cat. No.	Host Species
Akt	-	Polyclonal	CST	9272	Rabbit
AMPK α	D5A2	Monoclonal	CST	5831	Rabbit
ATF4	D4B8	Monoclonal	CST	11815	Rabbit
c-Abl	K-12	Polyclonal	Santa Cruz	sc-131	Rabbit
c-Myc	9E10	Monoclonal	Santa Cruz	sc-40	Mouse
CDK4	D9G3E	Monoclonal	CST	12790	Rabbit
Cleaved caspase 3	5A1E	Monoclonal	CST	9664	Rabbit
Cyclin A2	EPR17351	Monoclonal	abcam	ab181591	Rabbit
Cyclin D1	92G2	Monoclonal	CST	2978	Rabbit
EGFR	D38B1	Monoclonal	CST	4267	Rabbit
ERK1/2	137F5	Monoclonal	CST	4695	Rabbit
FAK	D2R2E	Monoclonal	CST	13009	Rabbit
FZD7	-	Polyclonal	abcam	ab64636	Rabbit
ITGA5	-	Polyclonal	HPA	HPA002642	Rabbit
Ki-67	-	Polyclonal	Invitrogen	PA5-19462	Rabbit
LC3B	D11	Monoclonal	CST	3868	Rabbit
NRP1	EPR3113	Monoclonal	abcam	ab81321	Rabbit
p-Akt (S473)	D9E	Monoclonal	CST	4060	Rabbit
p-AMPK α (T172)	40H9	Monoclonal	CST	2535	Rabbit
p-c-Abl (Y245)	-	Polyclonal	CST	2861	Rabbit
p-cdc2 (Y15)	10A11	Monoclonal	CST	4539	Rabbit
p-eIF2 α (S51)	D9G8	Monoclonal	CST	3398	Rabbit
p-ERK1/2 (T202/Y204)	D13.14.4E	Monoclonal	CST	4370	Rabbit
p-FAK (Y397)	D20B1	Monoclonal	CST	8556	Rabbit
p-FAK (Y925)	-	Polyclonal	CST	3284	Rabbit
p-Histone H3 (S10)	D2C8	Monoclonal	CST	3377	Rabbit
p-p70 S6K (T389)	D5U1O	Monoclonal	CST	97596	Rabbit
p-PLC γ (Y783)	-	Polyclonal	CST	2821	Rabbit
p-STAT3 (Y705)	D3A7	Monoclonal	CST	9145	Rabbit
p-Wee1 (S642)	D47G5	Monoclonal	CST	4910	Rabbit
p70 S6K	49D7	Monoclonal	CST	2708	Rabbit
PAR	E6F6A	Monoclonal	CST	83732	Rabbit
PARP	-	Polyclonal	CST	9542	Rabbit
PIK3R3	D2B3	Monoclonal	CST	11889	Rabbit
Rac	102	Monoclonal	BD Transduction Laboratories	610650	Mouse
RAS	-	Monoclonal	abcam	ab55391	Mouse
SDC4	-	Polyclonal	CST	12236	Rabbit
STAT3	124H6	Monoclonal	CST	9139	Mouse
Survivin	71G4B7	Monoclonal	CST	2808	Rabbit
β-actin	AC-74	Monoclonal	Sigma-Aldrich	A5316	Mouse

Supplemental Methods

Development and screening of a phytochemical library

Four-hundred twenty-two kinds of plant extracts from herbal or edible plants were collected (Supplemental Table 1) to construct our new phytochemical library. K562 (a human myeloid leukemia cell line) was used for cytotoxicity screening for each extract. Briefly, K562 cells were seeded at the concentration of 0.5×10^5 cells/mL onto 6-well culture plates. Subsequently, the cells were treated with the extracts dissolved at the concentration of 10 μg/mL in DMSO. After 48 hours of incubation, the viable cell percent was calculated by using the trypan-blue exclusion test.

Identification of active ingredients by use of High-Performance Liquid Chromatography (HPLC)

The extract from *Petasites japonicus* (10 μg/mL) was analyzed by using LC-PDA-VP (Shimadzu, Kyoto, Japan) with the column of Tskgel-80Ts 4.6 mm × 15 cm (Tosoh Corporation, Tokyo, Japan). The analysis conditions were the following: column temperature, 40°C; eluent, solution A (0.1% formic acid water solution), solution B (0.1% formic acid-acetonitrile); elution (solution A/B = 50/50), measurement duration, 60 min; flow rate, 1 mL/min; detection, 235 nm.

Purification of petasin

Petasin (PT) used in the study was purified in-house from the bulk extract of *Petasites japonicus*. Dried powder (100 g) of *Petasites japonicus* was mixed with 1.2 L of distilled water, and heated for 30 minutes at 95°C. After filtration through gauze and subsequent vacuum concentration, the concentrated extract was reconstituted in 150 mL of distilled water. The bulk extract solution was then subjected to further extraction with 200 mL of hexane in a separatory funnel; and then, the remaining water layer was subjected to repeated hexane extraction with 200 mL of hexane. The total 400 mL of hexane containing the bulk extract was paper-filtered and vacuum concentrated. The concentrated extract was reconstituted in hexane and separated by using step-wise column chromatography under the following conditions: column, r=2.5 cm × 15 cm; Merch silica gel 60, 0.063-0.200 nm; elution, Hex (hexane):AcOEt (ethyl acetate) = 40:1, 20:1, 10:1, 5:1, 0:1; 800 mL/each condition. Each fraction was separately collected, and one containing PT was determined by using HPLC. The fraction containing PT was subjected to the column chromatography for purification of PT under the following conditions: first elution, Hex:AcOEt = 8:1; second elution, CH_2Cl_2:EtOH = 150:1. The purified PT was dissolved in ethanol and confirmed to show a single spot on thin-layer chromatograms (CH_2Cl_2:AcOEt = 20:1, detected in anisicaldehyde). The PT solution was filtered through a

0.45 μm-pore filter, and the purity was confirmed to be over 98% by determining the absorbance at 235 nm. The conditions used for LC were same as described above.

Spheroid formation assay

The spheroid formation assay was performed by using a Cultrex 3-D spheroid Colorimetric Proliferation/Viability Assay Reagent Kit (Cat. No. 3511-096-K, Trevigen, Gaithersburg, MD, USA). Briefly, approximately 3,000 B16F10 cells were suspended in DMEM with 10% spheroid formation ECM reagent and seeded into a 96-well spheroid formation plate. Subsequently, the culture plate was centrifuged at 200 xg for 3 minutes at room temperature. After incubation at 37°C for 24 hours, the spheroids were formed and treated with DMSO or PT at the concentration of 3 or 30 μM for 9 days at 37°C. Phase-contrast microscopic images were obtained every 24 hours by using an microscope (CKX41, Olympus, Tokyo, Japan) and capture device (U-TU1XC, Olympus). The area of spheroids was calculated from the images by using Image J (version 2.0.0-rc-69/1.52p, NIH, MD, USA).

Cell-cycle analysis

Cell-cycle progression was analyzed by quantification of cellular DNA content by using the propidium iodide (PI) staining method (Tali Cell Cycle Kit, Thermo Fisher Scientific, MA, USA). Briefly, B16F10, A2058, MiaPaCa-2 or K562 cells treated with PT (3 μM) or DMSO were washed twice with PBS and centrifuged at 500 xg for 5 minutes. Fixation was performed with ice-cold 70% ethanol. To avoid cell clumping during fixation, cells were slowly added to the 70% ethanol in a drop-wise manner with gentle vortexing. After overnight incubation at -20°C, the cells were centrifuged at 1,000 xg for 5 minutes at 4°C and washed with 1 mL of PBS. The cells were then stained with 200 μL of the Tali Cell Cycle Solution containing PI for 30 minutes at room temperature in the dark. Thereafter, they were resuspended by brief vortexing, and 25 μL of stained cells was applied onto Tali Cellular Analysis Slides. The slides were loaded into the Tali Image-Based Cytometer (Thermo Fisher Scientific), and 20 fields per sample were captured with the function of the Tali Cell Cycle Assay. The data obtained were analyzed with JMP (version 12.2, SAS Institute, NC, USA) and GraphPad Prism 8 (version 8.4.0, GraphPad Software, CA, USA).

Measurement for glucose, lactate, and pH in culture medium

The glucose concentration in the culture medium was quantified by using a colorimetric WST-based glucose assay kit (Glucose Assay Kit-WST, G264, Dojindo, Kumamoto, Japan). Briefly, 50 μL of the samples (culture medium diluted to 1:200) was applied to a 96-well

microplate and mixed with 50 μL of the working solution containing glucose dehydrogenase (GDH) and dye mixture. After incubation at 37 °C for 15 min, absorbance at 450 nm was measured with a microplate reader (iMark Microplate Absorbance Reader, Cat. #168-1130, Bio-Rad) and used for calculating the relative concentration.

The lactate concentration in the culture medium was quantified by using a colorimetric-based assay kit (Lactate Colorimetric/Fluorometric Assay Kit, K607-100, BioVision, CA, USA). Briefly, culture medium was filtered through a 10-kDa molecular weight spin filter (#1997, BioVision) and deproteinized to remove FBS-derived lactate dehydrogenase (LDH). The deproteinized samples were then diluted to 1:5000 with the provided assay buffer, and 50 μL of the diluted samples was applied to each well of a 96-well microplate and mixed with 50 μL of the working solution containing lactate enzyme mix and probe. After incubation at room temperature for 30 min, absorbance at 570 nm was measured with a microplate reader (iMark Microplate Absorbance Reader, Cat. #168-1130, Bio-Rad) and used for calculating the relative concentration.

pH in the culture medium was directly measured by using an LAQUAtwin pH meter (pH-11B, HORIBA, Kyoto, Japan).

Transmission Electron Microscopy (TEM)

B16F10 cells were treated with PT (3 μM), metformin (5 mM) or DMSO at 72 h. After having been washed with ice-cold PBS, the cells were fixed for 2 hours with 2% paraformaldehyde and 2.5% glutaraldehyde in 0.2 M PBS (pH 7.4). They were next washed with PBS and post-fixed in 2% osmium tetraoxide for 2 hours. After PBS washes, the cells were progressively dehydrated by passage through a 10% graded series of 30–100% ethanol and cleared with QY-1 (Nissin EM, Tokyo, Japan). The cells were then embedded in Epon 812 resin (TAAB Laboratories Equipment, Reading, UK) and sectioned at a 70-nm thickness. After the sections had been stained with uranyl acetate and lead citrate, transmission electron microscopic images were obtained with a Hitachi-7650 operating at 80 kV (Hitachi, Tokyo, Japan).

ATP/ADP ratio

The ATP/ADP ratio was measured by using a luciferase-based assay kit (ADP/ATP Ratio Assay Kit, MAK135, Sigma-Aldrich). In brief, cells (1750 cells/well) were cultured and treated with compounds in a 96-well microplate. The culture medium was removed at the time of measurement, and 40 μL of the ATP reagent containing enzyme and substrate was added to each well and mixed. After a 1-min incubation, luminescence for ATP (RLUA) was measured with a luminometer (GLOMAX 20/20 Luminometer, E5311, Promega). The samples were further

incubated for 10 min, and then luminescence was measured for the residual ATP signals (RLUB). Immediately thereafter, 2 μL of ADP reagent containing the enzyme converting ADP to ATP was added to each well and mixed by pipetting. After incubation for 1 min, total signal (ADP and the residual ATP signals) was measured with the luminometer (RLUC). The ATP/ADP ratio was calculated by using the following formula: ATP/ADP ratio = RLUA/(RLUC-RLUB).

Senescence-associated-β-galactosidase staining

A Cellular Senescence Detection Kit (SPiDER-βGal, SG03, DOJINDO, Kumamoto, Japan) was used for the detection of senescence-associated-β-galactosidase activity. In brief, B16F10 cells were seed into 6-well plates at the concentration of 0.5×10^5 cells/mL and treated for 48 hours with PT (3 μM), positive controls (doxorubicin, DOX, 0.2 μM; etoposide, ETP, 10 μM) or negative control (DMSO). After 2 washes with DMEM containing 10% FBS, the cells were pre-treated with the provided Bafilomycin A1 solution for 1 hour in a 5% CO_2 incubator. After removal of the Bafilomycin A1 solution, the cells were subsequently treated for 1 hour with DMEM containing the provided SPiDER-βGal and Bafilomycin A1 solutions along with 10% FBS in a 5% CO_2 incubator. After 2 washes with PBS, the cells were trypsinized and resuspended in DMEM with 10% FBS. Thereafter, the data for fluorescence (Excitation: 488 nm, Emission: 500-600 nm) and cell counts were obtained by using the Tali Image-Based Cytometer (Thermo Fisher Scientific). The data were analyzed with GraphPad Prism 8 (version 8.4.0, GraphPad Software).

MitoTracker Orange/Green staining

B16F10 cells treated with PT (3 μM) or DMSO for 72 hours were incubated with 100 nM MitoTracker Orange and Green (Molecular Probes) for 30 min. After 2 washes with PBS, the cells were trypsinized; and 25 μL of the resulting single-cell suspension was applied onto Tali Cellular Analysis Slides. The slides were loaded into the Tali Image-Based Cytometer, and 20 fields per sample were captured with the function of the Green/Red analysis. The data obtained were analyzed with the JMP (version 12.2) and GraphPad Prism 8 (version 8.4.0) software.

Mitochondrial electron transport chain complex activity assays

The electron transport chain complex (ETC) activities were assessed by using MitoCheck Activity Assay Kits (complex I, Cat. #700930; complex II, Cat. #700940; complex II/III, Cat. #700950; complex IV, Cat. #700990; complex V, Cat. #701000; Cayman Chemical Company, MI, USA).

For assessment of the ETC complex I activity, test compounds (PT, metformin, phenformin), and positive control (Rotenone, 1 μM) were incubated in a 96-well plate with buffer containing bovine heart muscle-derived uncoupled mitochondria and KCN (1 mM). The solution containing mitochondria and test compounds were incubated for 15 minutes at 25°C. Subsequently, buffer containing NADH and ubiquinone was added to the solution. Immediately, the 96-well plate was placed in a plate reader (GloMax Discover Microplate Reader, Promega, WI, USA), and the absorbance at 340 nm was measured at 30-second intervals for 15 minutes at 25°C. The rate of NADH oxidation, which represents the activity of the ETC complex I, was calculated from the decrease in absorbance at 340 nm. The reaction rate was determined by calculating the slope for the linear portion of the curve and used for determining the IC_{50} value for each compound.

For the assessment of the ETC complex II activity, the test compound (PT) and positive control (2-Thenoyltrifluoroacetone, TTFA; 1 mM) were incubated in a 96-well plate with buffer containing bovine heart muscle-derived uncoupled mitochondria, rotenone (2 μM), antimycin A (20 μM), and KCN (1 mM). The solution containing mitochondria and test compound or positive control was incubated for 15 minutes at 25°C. Subsequently, buffer containing succinate, ubiquinone, and DCPIP was added to the solution. After incubation for 5 minutes at 25°C, the 96-well plate was placed in a plate reader (GloMax Discover Microplate Reader, Promega), and absorbance at 600 nm was measured at 30-second intervals for 15 minutes at 25°C. The rate of DCPIP oxidation, which represents the activity of the ETC complex II, was calculated from the decrease in absorbance at 600 nm. The reaction rate was determined by calculating the slope for the linear portion of the curve and used for determining the IC_{50} value for each compound.

For the assessment of the ETC complex II/III activity, the test compound (PT) and positive control (Antimycin A, 10 μM) were incubated in a 96-well plate with buffer containing bovine heart muscle-derived uncoupled mitochondria, rotenone (2 μM), and KCN (1 mM). The solution containing mitochondria and test compound or positive control was incubated for 15 minutes at 25°C. Subsequently, buffer containing succinate and cytochrome c was added to the solution. Immediately, the 96-well plate was placed in a plate reader (GloMax Discover Microplate Reader, Promega), and the absorbance at 550 nm was measured at 30-second intervals for 15 minutes at 25°C. The rate of cytochrome c reduction, which represents the activity of the ETC complex II/III, was calculated from the decrease in absorbance at 550 nm. The reaction rate was determined by calculating the slope for the linear portion of the curve and used for determining the IC_{50} value for each compound.

For the assessment of the ETC complex IV activity, the test compound (PT) and positive

control (KCN, 1 mM) were incubated in a 96-well plate with buffer containing bovine heart muscle-derived uncoupled mitochondria. The solution containing mitochondria and test compound or positive control was incubated for 15 minutes at 25°C. Subsequently, buffer containing reduced cytochrome c was added to the solution. Immediately, the 96-well plate was placed in a plate reader (GloMax Discover Microplate Reader, Promega), and the absorbance at 550 nm was measured at 30-second intervals for 15 minutes at 25°C. The rate of cytochrome c oxidation, which represents the activity of the ETC complex IV, was calculated from the decrease in absorbance at 550 nm. The reaction rate was determined by calculating the slope for the linear portion of the curve and used for determining the IC_{50} value for each compound.

For the assessment of the ETC complex V (ATPase) activity, the test compound (PT) and positive control (Oligomycin, 4 μM) were incubated in a 96-well plate with buffer containing bovine heart muscle-derived uncoupled mitochondria and rotenone (2 μM). The solution containing mitochondria and test compound or positive control was incubated for 15 minutes at 25°C. Subsequently, buffer containing enzymes (pyruvate kinase and lactate dehydrogenase), NADH, and ATP was added to the solution. Immediately, the 96-well plate was placed in a plate reader (GloMax Discover Microplate Reader, Promega), and the absorbance at 340 nm was measured at 30-second intervals for 30 minutes at 25°C. The conversion rate of ATP to ADP, which represents the activity of the ETC complex V as an ATPase, was indirectly calculated by a decrease in the absorbance of NADH at 340 nm. The reaction rate was determined by calculating the slope for the linear portion of the curve and used for determining the IC_{50} value for each compound.

NDI1 overexpression

A plasmid vector for *NDI1* overexpression in mammalian cells was generated by inserting *NDI1* sequences from cDNA of budding yeast (*Saccharomyces cerevisiae*) into the pF5A CMV-neo Flexi Vector (C9401, Promega). A2058 cells seeded at the concentration of 1×10^5 cells/mL onto a 6-cm dish were transfected with 8 μg of the vector and the Lipofectamine 2000 transfection reagent (Cat. #11668019, Thermo Fisher Scientific) in DMEM containing 10% FBS. After transfection, the cells underwent selection for 2 weeks through growth in 500 μg/mL of Neomycin (N1142, Sigma-Aldrich). The overexpression of *NDI1* was confirmed by the amplification of *NDI1* mRNA by the RT-qPCR technique.

Generation of Rho-0 cells

A2058 cells devoid of mitochondrial DNA (mtDNA), designated as A2058 Rho-0 cells, were generated according to the procedure previously reported (1). Briefly, A2058 cells were

cultured for 2 months in αMEM (Wako) supplemented with 10% FBS, 1.5 g/500 mL of glucose, and 500 ng/mL of ethidium bromide (Cat. # 315-90051, Nippon Gene, Tokyo, Japan). After confirming the up-regulation of glucose consumption and lactate production due to dysfunction of the ETC complex, the mitochondrial DNA was extracted and amplified with qPCR to check for the lack of *ND6*, one of mtDNA genes.

NAD/NADH ratios

An NAD/NADH Assay Kit (Cat. # N509, DOJINDO) was used for the measurement of NAD/NADH ratios. B16F10 cells were treated with PT (3 μM) for 24 hours. After 3 washes with ice-cold PBS, the cells were lysed in 300 μL of NAD/NADH extraction buffer. After centrifugation at 12,000 xg for 5 minutes, 250 μL of the supernatant was transferred to an MWCO 10K filtration tube. After subsequent centrifugation at 12,000 xg for 10 minutes, 100 μL of the filtrate was separated and transferred into two 1.5-mL microtubes for measurement of NADH or total NAD/NADH. The sample for NADH measurement was incubated at 60°C for 60 minutes, whereas the sample for measuring total NAD/NADH was kept on ice. After cooling the sample solution down to room temperature, 100 μL of NAD/NADH control buffer was added to each sample solution. The sample solutions (50 μL) and a mixture of the WST enzymes and dye (50 μL) were added to each well of a 96-well plate. After incubation at 37°C for 60 minutes, the absorbance of WST formazan at 450 nm was measured by using a microplate reader (iMark Microplate Absorbance Reader, Cat. #168-1130, Bio-Rad) to determine the amount of total NAD/NADH and NADH.

Metabolome analysis

B16F10 cells were seeded at a concentration of 0.5 × 10^5 cells/mL into 60-mm dishes. The cells were treated with PT (3 μM), metformin (5 mM), phenformin (50 μM) or DMSO for 9 or 48 hours. The samples for metabolome analysis were collected by using the methanol-chloroform extraction method. In brief, after 3 washes with ice-cold 5% mannitol, the cells on culture dishes were treated with 800 μL of ice-cold methanol extract solution containing 25 mM concentrations of internal standards (methionine sulfone, 2-(N-morpholino)ethanesulfonic acid, and D-camphor-10-sμLfonic acid; Wako). After incubation for 10 minutes, 400 μL of the extract solution was collected and mixed with 400 μL of chloroform and 200 μL of distilled water. After centrifugation at 10,000 xg for 3 min at 4°C, 400 mL of the aqueous layer was collected and subjected to filtration with ultrafreeMC-PLHCC for Metabolome Analysis (Human Metabolome Technologies, Tsuruoka, Yamagata, Japan) to remove proteins. The filtrates were dried-out and stored until analysis. The samples were analyzed by using capillary

electrophoresis (CE) and time-of-flight mass spectrometry (TOFMS) with the Agilent CE Capillary Electrophoresis System (Agilent Technologies, CA, USA). CE was performed under the following conditions: Capillary, COSMO(+), i.d. 50 μm × 105 cm; Buffer, 50 mM Ammonium acetate, pH 8.5; Voltage, negative, 30 kV; Temperature, 20°C; Injection, Pressure injection 50 mbar, 30 sec (approximately 30 nL); Preconditioning, 2 min at 50 mM Ammonium acetate, pH 3.4 and 5 min at run buffer). TOFMS was performed under the following conditions: Ion Source, ESI; Polarity, negative; Capillary voltage, 3,500 V; Fragmentor, 100 V; Skimmer, 50 V; OCT RFV, 500 V; Drying gas, N2, 10 L/min; Drying gas temperature, 300°C; Nebulizer gas pressure, 7 psig; Sheath liquid, 5 mM Ammonium acetate in 50% MeOH / Water containing 0.01 μM Hexakis(2,2-difluoroethoxy) phosphazene; Flow rate, 10 μL/min; Lock mass, 2CH3COOH13Cisotope m/z 120.038339; Hexakis (2,2-difluoroethoxy)phosphazene+CH3COOHm/z 680.035541; ESI needle, Platinum). Data were acquired and analyzed with Master Hands software (version 2.17.2.15, Keio University, Yamagata, Japan), JMP (version 12.2, SAS Institute), and GraphPad Prism 8 (version 8.4.0, GraphPad Software).

cDNA microarray analysis

Total RNA extraction was performed with a Maxwell RSC simplyRNA Cells Kit (AS1390, Promega) and Promega Maxwell RSC (AS4500, Promega). A2058 cells were treated with PT (3 μM), metformin (5 mM), phenformin (50 μM) or DMSO. After 2 washes with PBS, the cells were lysed with 400 μL of homogenization solution containing 4 μL of 1-thioglycerol. Subsequently, 200μl of lysis buffer was added to 200 μL of the 1-thioglycerol/homogenization solution containing lysed cells. After vigorous vortexing for 15 seconds, all 400 μL of the lysate was transferred to a Maxwell RSC Cartridge. After 5 μL of the DNase I Solution had been added to the cartridge, the cartridge was applied to a Promega Maxwell RSC instrument, and total RNA was purified. The RNA integrities of the samples were checked by using an Agilent 2100 Bioanalyzer (Agilent, CA, USA). After labeling with Agilent Low input Amp Labeling Kit (Cat. #5190-2305, Agilent), the samples were hybridized on the SurePrint G3 Human GE microarray 8x60K Version 3.0 (Agilent) by using an Agilent G2545A Hybridization Oven (Agilent) overnight at 65°C. After 3 washes, the slides were dried and scanned with an Agilent Microarray Scanner System (G2565CA, Agilent). The raw signal data obtained were globally normalized at 75 percentiles by using the Subio platform (version 1.2.2, Subio inc., Kagoshima, Japan). Fold-changes were calculated based on $\log_2$ transformed signal intensity ratios between treatment (PT, metformin or phenformin) and DMSO samples. P-values were calculated by using the Student's t-test (paired, two-tailed) from 3 biological replicates. Genes that were 1.5-

fold or greater up- or down-regulated and showed P-values of less than 0.05 were considered as differentially expressed genes (DEGs). The DEGs were analyzed by using the metascape (2) with the database from the Kyoto Encyclopedia of Genes and Genomes (KEGG) and Reactome pathways. JMP (version 12.2, SAS Institute) was utilized for illustrating heatmaps. The circos plot was illustrated by using the metascape function.

Proteome analysis

B16F10 cells cultured in high-glucose DMEM with 10% FBS were treated for 72 hours with PT (3 µM) or DMSO. The cells were washed with ice-cold PBS twice on ice and lysed with 1 mL of 4% SDS-100mM Tris-HCI, pH8.5. Ice-cold acetone (4 mL) was then added to the samples, and incubation was carried out for 2 hours at -20°C. After centrifugation at 13,000 xg for 5 min, pellets from the samples were dissolved in 1 mL of 100 mM Tris pH 8.5-0.5% sodium dodecanoate (SDoD). Protein concentrations were assessed by using the BCA assay (Protein Assay BCA Kit, 297-73101, Wako), and the samples were diluted to 1 ug/uL with 100 mM Tris pH 8.5-0.5% SDoD. For cleavage of disulfide bonds, the samples were mixed with 20 µL of 10 mM DTT and incubated for 30 min at 50°C. For alkylation of the cysteine residues, the samples were mixed with iodoacetamide (IAA) to be 30 mM at the final concentration. After incubation for 30 min at room temperature, the IAA reaction was stopped by cysteine supplementation (incubation for 10 min at room temperature). After supplementation with 150 uL of 50 mM ammonium bicarbonate to increase the efficiency of enzymatic digestion, the samples were digested to peptide fragments by overnight incubation at 37°C with Lys-C (400 ng) and trypsin (400 ng).After removal of SDoD with 5%TFA, the samples were desalted by using C18 spin columns (MonoSpin C18, GL sciences, Tokyo, Japan). The samples were then evaporated and dissolved in 1 mL of 3% ACN-0.1% formic acid. The concentration of peptide were measured by the BCA assay (Protein Assay BCA Kit, 297-73101, Wako) and diluted to 100 ng/uL with 3% ACN-0.1% formic acid.

The samples were then analyzed by nanoLC-MS (nanoLC, UltiMate 3000 RSLCnano LC System, Thermo Fisher Scientific; MS, Q Exactive HF-X, Thermo Fisher Scientific) with a column (75 µm × 120 mm, Nikkyo Technos, Tokyo, Japan). The analysis conditions were the following: applied peptide amount: 100 ng; column temperature, 40°C; eluent, solution A (0.1% formic acid water solution), solution B (0.1% formic acid-80% acetonitrile); elution (solution A/B = 50/50), measurement duration, 40 min; flow rate, 150 nL/min. The data were analyzed by using Scaffold DIA (Proteome Software, OR, USA) under the following conditions: Protein Sequence Database, Mouse UniProtKB/Swiss-Prot database (UP000000589); Spectral Library, Prosit; Fragmentation, HCD; Precursor Tolerance, 8 ppm; Fragment Tolerance, 8 ppm; Data

Acquisition Type, Overlapping DIA; Digestion Enzyme, Trypsin; Peptide Length, 7-45; Peptide Charge, 2-4; Max Missed Cleavages, 1; Fixed Modification, Carbamidomethylation [C]; Peptide FDR, less than 1%; Protein FDR, less than 1%.

Animal experiments

All mouse experiments were approved by the Institutional Animal Care and Use Committee of Gifu University. All mice were maintained with *ad libitum* food and water under the standard light-dark cycle. Tumor volume was measured by using the following formula: $V = \pi/6(L \times W^2)$, where L, length; W, width of the tumor. No animal had a tumor that exceeded the maximal size permitted up to 20 mm in any direction.

Due to the limited availability of PT, the dose for the in vivo experiment was determined by performing a preliminary dose-increasing experiment using a small number of C57BL6/J mice (n=5, female, 6-week-old, SLC, Shizuoka, Japan). Firstly, 5 mice intraperitoneally received PT at the concentration of 25 mg/kg; and then the dosage was increased concentration up to 100 mg/kg. At the concentration of 100 mg/kg, 1 of the 5 mice experienced acute toxicities as seen in the case of other ETCC1 inhibitors, i.e., severe hypothermia and respiratory inhibition resulting in death. Thus, the maximum tolerated dose was estimated as approximately 100 mg/kg. Mice well-tolerated the concentration of 50 mg/kg; therefore, this concentration was employed for the in vivo experiments.

For the orthotopic B16F10 syngeneic model, B16F10 cells at the concentration of 0.5×10^6 cells/100 μL in PBS were subcutaneously injected into the right flank of C57BL6/J mice (female, 6-week-old, SLC; 8 mice per condition). After the mean tumor volume had reached approximately 100 mm^3, the mice were randomized and separated into 3 groups. Subsequently, the mice received intraperitoneal administration (once a day for a total of 4 injections) of PT (50 mg/kg), phenformin (50 mg/kg) or vehicle; i.e., PBS containing 1% v/v DMSO and 10% v/v high-purity oleic acid (HX2, NOF, Tokyo, Japan). The day after the final administration, the mice were sacrificed for the assessment of tumor tissues and normal organs.

For the time-course measurement of ATF4 expression in the subcutaneous B16F10 tumors in vivo, B16F10 cells at the concentration of 0.5×10^6 cells/100 μL in PBS were subcutaneously injected into the right flank of C57BL6/J mice (female, 6-week-old, SLC; 3 mice per condition, total 24 mice). After the mean tumor volume had reached approximately 100 mm^3, the mice were randomized and separated into 8 groups. Subsequently, the mice received a single intraperitoneal administration of PT (50 mg/kg, dissolved in PBS containing 1% v/v DMSO and 10% v/v high-purity oleic acid). The mice were sacrificed at 0.5, 1, 3, 6, 24, 48, 72 hours, and their tumor tissues were collected and fixed with neutral buffered 4% paraformaldehyde

for immunohistochemical analysis.

For the A2058 xenograft model, A2058 cells at the concentration of 5×10^6 cells/100 μL in matrigel (#356234, Corning, NY, USA) were subcutaneously injected into the right flank of BALB/c *nu/nu* mice (female, 4-week-old, SLC; 8 mice per condition). After the mean tumor volume had reached approximately 100 mm^3, the mice were randomized and separated into 2 groups. Subsequently, the mice received intraperitoneal administration (once a day for a total of 10 injections) of PT (50 mg/kg) or vehicle (PBS containing 1% v/v DMSO and 10% v/v high-purity oleic acid). The day after the final administration, the mice were sacrificed for the assessment of tumor tissues and normal organs.

For the NB-1 xenograft model, NB-1 cells at the concentration of 5×10^6 cells/100 μL in matrigel (#356234, Corning, NY, USA) were subcutaneously injected into the right flank of NOG mice (NOD/Shi-scid,IL-2RγKO Jic, female, 6-week-old, CLEA Japan, Tokyo, Japan; 8 mice per condition). After the mean tumor volume had reached approximately 100 mm^3, the mice were randomized and separated into 2 groups. Subsequently, the mice received intraperitoneal administration (once a day for a total of 14 injections) of PT (50 mg/kg) or vehicle (PBS containing 1% v/v DMSO and 10% v/v high-purity oleic acid). The day after the final administration, the mice were sacrificed for the assessment of tumor tissues and normal organs.

For the intravenous injection-based lung colonization model, B16F10 cells suspended in PBS at the concentration of 0.25×10^6 cells/100 μL were intravenously injected into C57BL6/J mice (female, 8-week-old, SLC; 5 mice per condition) via a tail vein. The mice were assigned to the treatment or control group through random cage selection and received intraperitoneal administration (every other day for a total of 7 injections) of PT (50 mg/kg) or vehicle (PBS containing 1% v/v DMSO and 10% v/v high-purity oleic acid). Two days after the final administration, the mice were sacrificed for counting lung colonies. The lung colonies were counted in a blinded manner by using a stereoscopic microscope (DSZ-70IFL, Carton, Tokyo, Japan).

For the spontaneous metastasis model, Jyg-MCB cells suspended in PBS at the concentration of 5×10^6 cells/100 μL were intravenously injected into the right flank of BALB/c *nu/nu* mice (female, 4-week-old, SLC; 8 mice per condition). After incubation for 24 days, the mice were randomized based on the subcutaneous tumor volume and separated into 2 groups. Subsequently, the mice received intraperitoneal administration (total of 6 injections for 16 days) of PT (50 mg/kg) or vehicle (PBS containing 1% v/v DMSO and 10% v/v high-purity oleic acid). Two days after the final administration, the mice were sacrificed for counting lung metastatic colonies and measuring weights of axillary lymph nodes. The lung metastatic spots

were visualized by infusing the lungs with neutral buffered 4% paraformaldehyde via the cannulated trachea. The metastatic spots were counted in a blinded manner by using a stereoscopic microscope (DSZ-70IFL, Carton, Tokyo, Japan).

Scratch wound healing assay

B16F10 and ASF 4-1 cells were seeded at the concentration of 2 × 10^5 cells/well in 6-well plates. After overnight incubation, the “wound” scratch was made on the monolayer cells by using a 200 µL plastic pipette tip. The cells were then treated with PT (3 mM) or DMSO, and images of the wounds were captured by using a phase-contrast microscope (CKX41 and DP72, Olympus, Tokyo, Japan) at 0, 7, 24, 31, and 48 hours after wounding. Wound closure percent was calculated by using ImageJ (NIH, MD, USA). The experiment was performed in high-glucose DMEM supplemented with 10% FBS.

Matrigel invasion assay

Invasion assays were performed by using 24-well Corning BioCoat Matrigel Invasion Chambers (Cat. #354480, Corning). After rehydration with DMEM (Wako) for 2 hours at 37°C, 5% CO_2 atmosphere, 250,000 B16F10 cells in DMEM containing PT (3 µM) or DMSO without FBS were plated on the top of the chambers. The cells were then allowed to invade for 24 hours through the matrigel-coated PET membrane onto its underside in contact with DMEM containing 10% FBS. The cells that did not invade were removed from the top of the matrigel-coated PET membrane surface with a cotton-tipped swab. The cells that had invaded were fixed with 100% methanol and stained with Giemsa stain solution (Wako). After 3 washes with distilled water, the chambers were air-dried, and the PET membranes were removed from the chambers and mounted on glass slides with MOUNT-QUICK (DAIDO SANGYO). The cells that had invaded through the matrigel and passed through the pores of the PET membrane onto its underside were counted by using a microscope (CKX41, Olympus).

Cell attachment assay

B16F10 cells were seeded at the concentration of 0.5 × 10^5 cells/mL in 10-cm dishes and treated for 24 hours with culture medium containing PT (3 µM) or DMSO. The cells were then trypsinized and suspended in compound-free EMEM 10% FBS at the concentration of 0.5 × 10^5 cells/mL in 6-well plates. After incubation for 3 hours in a humidified 5% CO_2 incubator, images for the cells were captured by using a phase-contrast microscope (CKX41 and DP72, Olympus). Adhesive/non-adhesive cell ratio was calculated by counting attached and non-attached cells. The experiment was performed in high-glucose DMEM supplemented with 10%

FBS.

Rac1 pulldown assay

The levels of Rac1-GTP in cell lysates were measured using by the GST-PAK-CRIB pulldown assay. GST-PAK-CRIB expressed in *Escherichia coli* was affinity purified with glutathione-Sepharose beads. B16F10 cells (1.0×10^6 cells/ dish) were seeded onto 6-cm culture dishes and treated for 48 hours with PT (0.3 μM) or DMSO. After 1 wash with ice-cold PBS, the cells were lysed with 400 μL of buffer containing Tris-HCl (20 mM, pH 8.0), 100 mM NaCl, 2 mM $MgCl_2$, 0.5% PT-40, 10% glycerol, and protease inhibitor solution (Roche, Basel, Switzerland). The cells were then harvested and centrifuged at 16,000 xg, 4°C for 10 minutes. The supernatant obtained was incubated for 1 hour with GST-PAK-CRIB-conjugated beads at 4°C. After 3 washes with the lysis buffer, proteins were eluted with Laemmli sample buffer and boiled for 5 minutes at 98°C. The supernatant, along with a non-pulldowned sample, was subjected to immunoblotting that was performed as described above. Anti-Rac1 antibody (Clone 102, Cat. No. 610650, BD Transduction Laboratories, NJ, USA) was used as a primary antibody for the detection of Rac1.

Immunoblotting

Cell and tissue lysates were prepared with 1% SDS buffer. In brief, cultured cells were washed with ice-cold PBS twice and fixed with 10% TCA (Wako). After incubation for 30 minutes on ice, the cells were washed with ice-cold PBS twice and then lysed in the cultured dishes with 1% SDS buffer (Wako). Tissue samples from tumors were snap-frozen by using liquid nitrogen. The frozen tissues were then homogenized in 1% SDS buffer (Wako). After sonication and centrifugation for 20 minutes at 13,000 xg, supernatants were collected; and protein concentrations were measured by using a DC Protein Assay Kit (Bio-Rad, CA, USA). Protein samples (1–5 μg/lane) were subjected to 10–12.5% sodium dodecylsulphate-polyacrylamide gel electrophoresis (SDS-PAGE) and transferred to 0.45-μm polyvinylidene fluoride membranes (Immobilon-P Membrane, EMD Millipore, MA, USA). After blocking with PVDF Blocking Reagent for Can Get Signal for 1 hour, the membranes were incubated overnight at 4°C with primary antibodies. The primary antibodies used for immunoblotting were summarized in Supplemental Table 2. The membranes were washed 3 times with TBS-T for 5 minutes each time and then incubated at room temperature with horseradish peroxidase-linked anti-mouse IgG or anti-rabbit IgG as secondary antibodies (Cell Signaling Technology) diluted 1:4000. After 3 washes with TBS-T, the immunoblots were visualized by using the Luminata Forte Western HRP substrate (EMD Millipore). The loaded amount was verified with

an anti-β-actin mouse monoclonal antibody (clone AC-74, Cat. #A5316, Sigma-Aldrich). Densitometry of the immunoblots was performed by using Image J (version 2.0.0-rc-69/1.52p, NIH). Due to a limited number of samples that can be run in the single gel at once, samples from an animal experiment (the subcutaneous orthotopic B16F10 syngeneic model) were blotted in 2 different membranes, and the raw densitometric signals were normalized with β-actin and a pooled control (PC), which was a mixture containing an equivalent amount of all samples examined. Serial dilutions of a representative sample were made to determined the optimal protein concentrations so that the blots would have signals in a linear dynamic range. The linearity of the signals was also confirmed in an *ex-post facto* manner by showing that the means of normalized values (log_2-transformed) were close to 0, based on the theory that the mean for signals of all samples would be equivalent to the signal of the pooled control.

Reference

1. Hashiguchi K, Zhang-Akiyama QM. Establishment of human cell lines lacking mitochondrial DNA. *Methods Mol Biol.* 2009;554:383-391.
2. Zhou Y, et al. Metascape provides a biologist-oriented resource for the analysis of systems-level datasets. *Nat Commun.* 2019;10(1):1523.
